# Engineering Nickel(II) Porphyrin-Conjugated Polymers with Different Aryl *meso*-Substituents for n-Type and p-Type Ammonia Sensors

**DOI:** 10.1021/acsami.4c15731

**Published:** 2024-11-25

**Authors:** Deepak Bansal, Sujithkumar Ganesh Moorthy, Marcel Bouvet, Nicolas D. Boscher

**Affiliations:** † Luxembourg Institute of Science and Technology (LIST), 28 Avenue des Hauts-Fourneaux, L-4362 Esch-sur-Alzette, Luxembourg; ‡ Institut de Chimie Moléculaire de l’Université de Bourgogne (ICMUB), UMR CNRS 6302, Université de Bourgogne, 9 Avenue Alain Savary, 21078 Dijon cedex, France

**Keywords:** conductometric gas sensor, ammonia sensing, bilayer heterojunction, polarity engineering, porphyrin, conjugated polymer, chemical vapor deposition

## Abstract

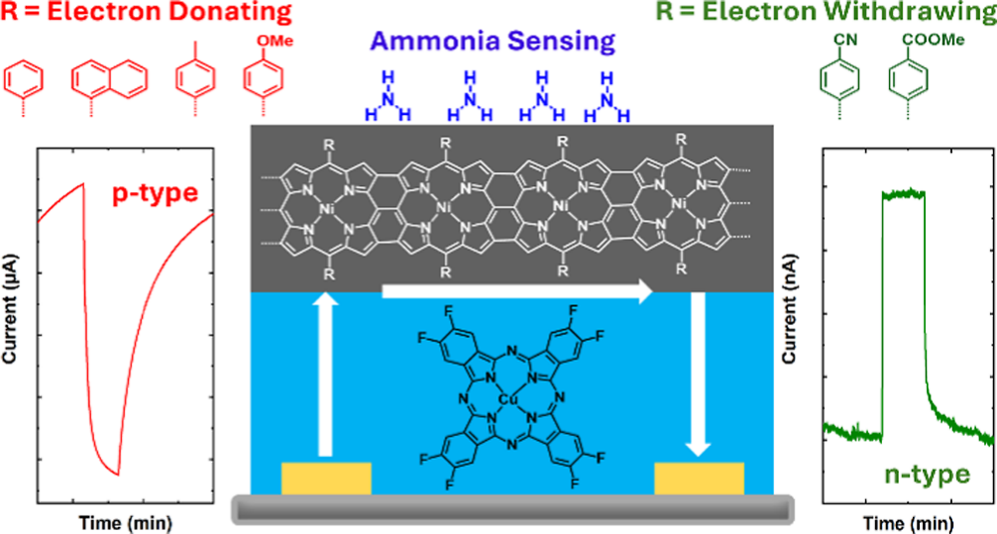

Conjugated polymers have revolutionized the field of conductometric gas sensors for sensing toxic gases arising from the fast urbanization and industrialization. In this work, we report the synthesis of a series of 5,15-diaryl Ni(II) porphyrin-conjugated polymers (**pNiD(Aryl)P**) and their integration as the top layer on an octafluorinated copper phthalocyanine (**CuF**_**8**_**Pc**) sublayer to construct bilayer heterojunction (BLH) devices for ammonia sensing. For the first time, we report the pioneering demonstration of polarity engineering within a BLH device by manipulating the *meso*-substituent of the 5,15-diaryl Ni(II) porphyrin-conjugated polymer constituting the top layer of the **CuF**_**8**_**Pc/pNiD(Aryl)P** BLH device. The BLH devices prepared from the 5,15-diaryl Ni(II) porphyrin-conjugated polymer bearing electron-donating *meso*-substituents as the top layer exhibit a p-type behavior, whereas an n-type behavior is observed for the BLH devices prepared from the 5,15-diaryl Ni(II) porphyrin-conjugated polymer bearing electron-withdrawing *meso*-substituents. Laser desorption ionization high-resolution mass spectrometry, UV/vis/NIR, and X-ray photoelectron spectroscopy studies provide evidence of a decrease in intramolecular dehydrogenative coupling in **pNiD(Aryl)P** bearing electron-withdrawing *meso*-substituents, resulting in low electrical conductivity of the thin films. Density functional theory calculations reveal noninvolvement of electron-withdrawing *meso*-substituents toward π-delocalization in the fused Ni(II) porphyrin tapes. Interestingly, all the **CuF**_**8**_**Pc**/**pNiD(Aryl)P** BLH devices exhibit remarkable sensing response toward NH_3_. Among all the devices, **CuF**_**8**_**Pc/pNiDPP** displays the highest sensitivity of −1.17% ppm^–1^ for NH_3_, whereas **CuF**_**8**_**Pc/pNiDNapP** and **CuF**_**8**_**Pc/pNiDCNPP** exhibit the best limit of detection for NH_3_, below 200 ppb. In addition, **CuF**_**8**_**Pc/pNiDCNPP** shows short response and recovery times of 13 and 255 s, respectively, making this device highly suitable for deployment in emergency services.

## Introduction

Urbanization and industrialization result in the release of large amounts of harmful and pollutant gases like CO, SO_2_, NO_2_, NH_3_, and H_2_S and volatile organic compounds (VOCs) such as benzene, toluene, ethanol, acetaldehyde, and formaldehyde into the environment with detrimental effects to humans and the surrounding atmosphere.^1−3^ Therefore, timely detection of these gases is very important for different sectors such as industries, air quality monitoring, public care, and mines.^4−7^ Numerous sensing techniques based on electrochemical and capacitive transducers, or optical fibers and quartz crystal microbalances, have been developed and used for this purpose.^8,9^ However, the widespread utilization of most of these techniques is limited due to relatively high prices, low sensitivity and selectivity, sophisticated design, and lack of portability.^10^

Conductometric gas sensors (CGSs) have been considered as a promising technology for the accurate and simple detection of harmful gases and VOCs in various applications.^11−13^ CGSs rely on a change in the conductivity of materials upon interaction with gas molecules.^14−16^ Therefore, the ability to conduct charges is the foremost requirement for CGSs. In 1960, Seyama et al. demonstrated a ZnO thin film as an effective sensing layer toward propane, showcasing the preparation of simplistic electrical devices for gas sensing.^17^ Since then, there has been an exponential growth in the number of reports on the metal oxide-based CGSs.^18−20^ Although the intensively studied metal oxide-based CGSs offer several advantages like low-power, low-cost, small-size, durability, ease of fabrication, and high sensitivity, they suffer from poor selectivity in the presence of interfering gases and require high operating temperatures, resulting in high signal-to-noise ratios.^20^

The recent developments of conjugated polymers provide an effective alternative to metal oxide dominance in CGSs by operating on the phenomenon of absorption or desorption of gas. The tunability, durability, and their capability to operate at ambient temperature make conjugated polymers such as polypyrrole, polyaniline (PANI), polythiophene, and poly(3,4-ethylenedioxythiophene) (PEDOT) attractive as CGS materials.^21,22^ In particular, PANI-based materials are largely exploited for their sensing properties toward NH_3_. Similar to metal oxide-based CGS materials, the sensing properties of conjugated polymers are influenced by their structure and morphology.^23−25^ Moreover, the possibility to modulate a polymeric structure by introducing groups equipped with interacting sites and their adaptability in various forms like nanofibers, nanoparticles, and thin films empowers scientists to tailor-made sensors for desired sensing requirements.^26,27^ However, despite several advantages, conjugated polymer-based sensors also face limitations in terms of low sensitivity, slow response and long recovery times, poor thermal stability, and limited selectivity, which seldom constrain their larger utility.

**Scheme 1:**
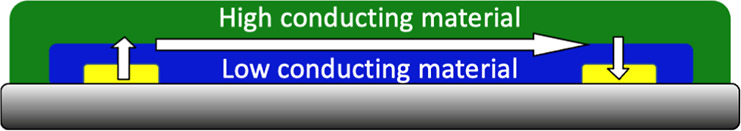
Schematic View of a BLH Device. The Arrow Indicates the Main Path for Charges

The combination of metal oxides with PANI has shown that alterations in the microstructure and the creation of p–n heterojunctions considerably improves the gas sensing capabilities of the materials.^28,29^ Such improvement was also observed in 2D-based p–n junctions that exhibit enhanced sensing performances compared to unipolar p-type or n-type 2D sensors.^30^ In a molecular material-based heterojunction, in-depth studies revealed that these p–n heterojunctions mainly benefit from the accumulation of opposite charges (e^–^ and h^+^) at the interface of a bilayer coating owing to the work function difference between the two semiconducting layers, enhancing the charge carrier mobility along the interface.^29−33^ Parra and Bouvet designed and patented bilayer heterojunction (BLH) sensing devices combining a low conducting material as a sublayer with a highly conducting material as a top layer (Scheme 1).^34^ Numerous sublayers, including monophthalocyanine complexes, sexithiophene, perylene derivatives, triphenodioxazine,^35^ and even with an inorganic semiconductor, namely, tungsten trioxide, in a low conducting state,^36^ have been studied as sublayers. On the other hand, the studied top layers almost exclusively rely on lutetium bisphthalocyanine (LuPc_2_), which is an intrinsic molecular semiconductor, with a high conductivity because of its radical nature that facilitates the electron transfer between molecules. When exposed to target gases, the nature of the response of such devices is reported to depend on the nature of the majority free charge carriers in the sublayer.^37^

During the past decade, there was a growing interest in investigating new and versatile conjugated polymers in the perspective to further improve the gas sensing properties of CGSs. In this context, metal porphyrinoids and related compounds offer rich chemistry for their application in organic heterojunction sensors.^31−33^ Different methodologies have been adopted to synthesize and fabricate conjugated polymers to achieve BLH sensors. Recently, Kumar et al. demonstrated in situ synthesis of Zn(II) polyporphine using an electropolymerization technique.^31^ The combination of a polyporphine sublayer and a highly conducting LuPc_2_ top layer induced a p-type behavior of the polyporphine/LuPc_2_ BLH device for NH_3_ sensing. They showed that the change in oxidation potential for electrodeposition leads to the formation of a singly (pZnP1) or doubly (pZnP2) fused Zn(II) polyporphine sublayer resulting in the distinctive responsive behavior of polyporphine/LuPc_2_. While a singly fused pZnP1 sublayer in pZnP2/LuPc_2_ exhibits reversible and stable sensing response with the relative response (RR) of nearly 47% in inert and 23.6% under humid conditions, pZnP2/LuPc_2_ displays unstable and poor RR (7.8%) owing to diffusion of NH_3_ within the pZnP2 volume. In 2023, Bouvet et al. replaced the pZnP sublayer by in situ electropolymerized Cu(III) polycorrole (pCuC) and developed a bilateral heterojunction device comprising LuPc_2_ as the top layer.^32^ Gratifyingly, the synthesis of the pCuC/LuPc_2_ device in the presence or absence of lutidine as a base exhibits a change in sensing nature toward ammonia from p-type in the former to n-type in the latter. It is speculated that in the absence of lutidine, copper polycorrole remains majorly protonated, exhibiting an electron-deficient nature resulting in an n-type behavior. In 2020, our group presented Ni(II) porphyrin-based multiply fused conjugated polymers (pNiP) as an alternative to the LuPc_2_ top layer, using oxidative chemical vapor deposition (oCVD), over the low conducting CuPc or CuF_16_Pc sublayer and investigated their gas sensing properties.^33^ As mentioned before, changing the sublayer from CuPc/pNiP to CuF_16_Pc/pNiP results in the reversal of sensing behavior of the device from p-type to n-type, respectively. Taking advantage of the positions of frontier orbitals of CuF_8_Pc, just in between those of CuPc and CuF_16_Pc, as depicted from photoelectron spectroscopy analyses,^38^ the CuF_8_Pc/LuPc_2_ heterojunction was reported, which exhibits ambipolar electronic properties, changing from p-type to n-type depending on an external trigger.^39^

Interestingly, metalloporphyrins (MPor) offer the advantage to tune their electronic and optoelectronic properties of a conjugated polymer by the incorporation of substituents to one or several of the eight β- and four *meso*-positions of the porphyrin ring and/or from the introduction of a cation inside the porphyrin core.^40−42^ Moreover, the electronic properties of the substituent on MPor directly affect the structural properties of MPor-conjugated polymers,^42^ which in turn can impact the sensing response of the BLH devices toward ammonia. Building on the rich chemistry of MPors and the advantages of oCVD, which overcomes many of the limitations related to solution-based approaches and enable the synthesis, deposition, and engineering of metalloporphyrin-conjugated polymer thin films on virtually any substrate,^40^ herein we report the engineering of a series of multiply fused disubstituted Ni(II) porphyrin-conjugated polymer thin films containing different aryl synthons at 5,15-position. These directly fused *meso*-substituted Ni(II) porphyrin-conjugated polymers are readily deposited onto the Cu(II) octafluoro-phthalocyanine (CuF_8_Pc) sublayer using oCVD to prepare a variety of BLH devices (Scheme S1), while a previous report demonstrated the change in sensing response on replacing the sublayer in a BLH device. For the first time, we showcase the engineering in sensing response toward NH_3_ between p-type and n-type by manipulating the top layer. The detailed characterization of multiply fused Ni(II) porphyrin-conjugated polymers, including the influence of the porphyrin’s substituent on the intermolecular and intramolecular dehydrogenative coupling reaction and the electronic properties of the resulting thin films, is supported by ultraviolet–visible–near-infrared (UV/vis/NIR) spectroscopy, laser desorption ionization high-resolution mass spectrometry (LDI-HRMS), X-ray photoelectron spectroscopy (XPS), and conductivity measurements. Density functional theory (DFT) calculations are performed to understand the influence of substitution on the distribution of frontier molecular orbitals (FMOs) in the multiply fused Ni(II) porphyrin-conjugated polymer thin films.

## Results and Discussion

### Synthesis and Characterization of the Fused Porphyrin-Conjugated Polymer Thin Films

**Scheme 2:**
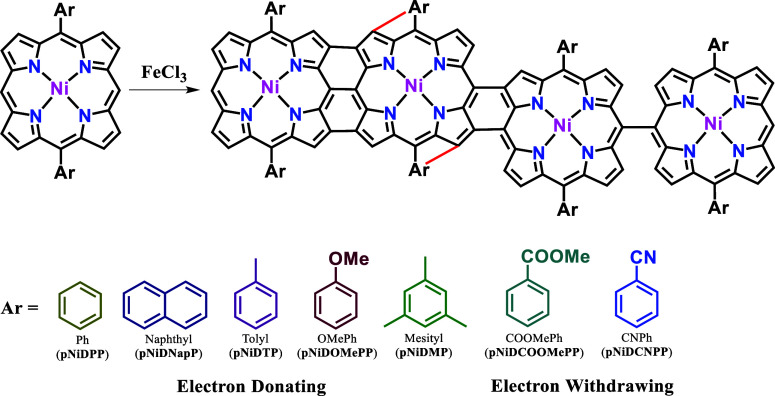
Schematic Representation of the oCVD Reaction of 5,15-Diaryl Ni(II) Porphyrins Intramolecular dehydrogenative coupling can occur between free *ortho*-positions of the aryl substituent and the adjacent β-positions of the porphyrin macrocycle (red). Molecular structures of the aryl substituents used in this work (from left to right): phenyl, naphthyl, tolyl, 4-methoxyphenyl, mesityl, 4-methoxycarbonylphenyl, and 4-cyanophenyl.

A series of fused porphyrin-conjugated polymer thin films (**pNiDPP**, **pNiDNapP**, **pNiDTP**, **pNiDOMePP**, **pNiDMP**,^43^**pNiDCOOMePP**, and **pNiDCNPP**) were prepared from the oCVD of 5,15-diaryl Ni(II) porphyrins bearing differently substituted phenyl synthons (see Experimental Section for details). When attached to the *meso*-positions, i.e. 5,15-positions, of the porphyrin macrocycle, aryl substituents with free *ortho*-positions are shown to promote intramolecular dehydrogenative coupling with the β-position of the porphyrin macrocycle.^40−42^ Such intramolecular dehydrogenative coupling induces a flatting of polymeric structure, resulting in extended π-electron conjugation in fused porphyrin-conjugated polymers and their π–π stacking. Moreover, the electronic properties of the aryl substituent considerably affect the extent of intramolecular dehydrogenative coupling, influencing the structural, electronic, and catalytic properties of the synthesized fused porphyrins-conjugated polymer thin films.^41,42^ Based on previous studies that highlighted the significance of intramolecular dehydrogenative coupling on the properties, including gas sensing properties,^33^ of fused porphyrin-conjugated polymers, a series of aryl substituents with free *ortho*-positions are investigated for the preparation of fused porphyrin-conjugated polymer thin films for gas sensing application. Phenyl synthons on Ni(II) porphyrins are categorized upon their (i) electron-donating (Ph, naphthyl, tolyl, OMePh, and mesityl) and (ii) electron-withdrawing (COOMePh and CNPh) characters. The molecular structures of investigated 5,15-substituted Ni(II) porphyrins are represented in Scheme 2.

**Figure 1:**
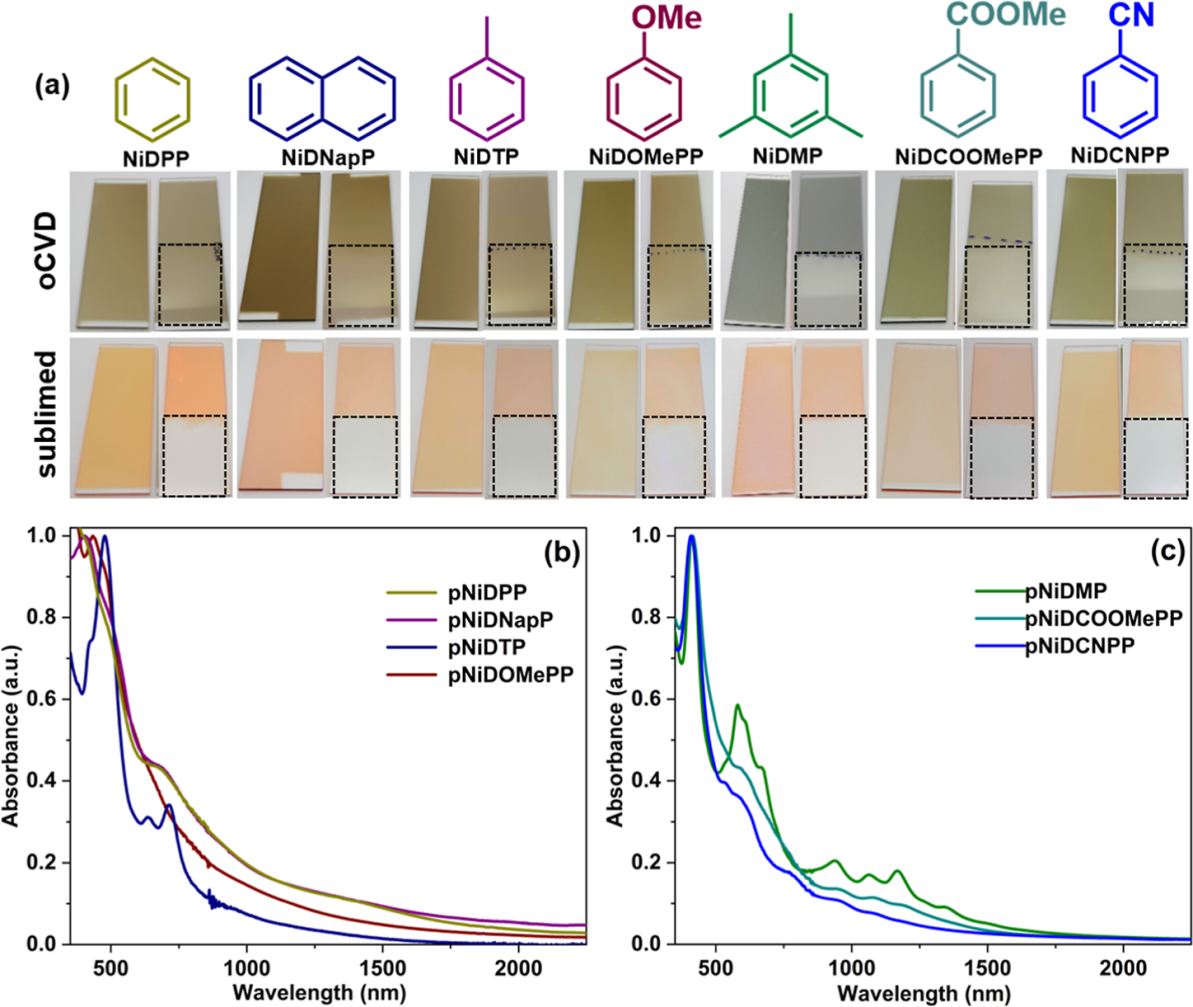
(a) Optical images of the as-deposited sublimed (orangish) and oCVD **pNiD(Aryl)P** (greenish) thin films and their respective DCM-rinsed film (dotted box) deposited on glass substrates from the 5,15-diaryl Ni(II) porphyrins studied in this work. UV/vis/NIR absorption spectra of the as-deposited oCVD **pNiD(Aryl)P** thin films prepared from 5,15-diaryl Ni(II) porphyrins bearing (b) electron-donating (**pNiDPP**, **pNiDOMePP**, **pNiDTP**, and **pNiDNapP**) and (c) electron-withdrawing (**pNiDCOOMePP** and **pNiDCNPP**) substituents. The UV/vis/NIR spectrum of **pNiDMP**, which does not exhibit any intramolecular dehydrogenative coupling, is shown for comparison.

The oCVD reactions of the 5,15-diaryl Ni(II) porphyrins were performed in a custom-built reactor using Fe(III) chloride as the oxidant. Irrespective of the aryl substituent investigated, the oCVD thin films all exhibited light to intense green coloration, which contrasts with the orangish coloration of the reference sublimed porphyrin thin films prepared from the sublimation of 5,15-diaryl Ni(II) porphyrins in the absence of Fe(III) chloride (Figure 1a). Such a change of color highly suggests the successful polymerization of all of the 5,15-diaryl Ni(II) porphyrins investigated.

The UV/vis/NIR spectra of all the oCVD thin films display a significant absorption extending to NIR regions, indicating the formation of fused porphyrin-conjugated polymers irrespective of the aryl substituent (Figure 1b,c). Interestingly, **pNiDOMePP**, **pNiDPP**, **pNiDTP**, and **pNiNapP** exhibit significantly broadened and red-shifted Soret and Q-bands in comparison to their sublimed counterparts (Figure S1), along with broad absorption feature between 1200 and 1600 nm (Figure 1b). This indicates a high extent of the intermolecular dehydrogenative coupling reaction, including intramolecular dehydrogenative coupling, whereas **pNiDCOOMePP** and **pNiDCNPP** containing di-4-methoxycarbonylphenyl (COOMePh) and 4-cyanophenyl (CNPh) synthons in Ni(II)-5,15-diarylporphyrin, respectively, show weakly shifted Soret and Q-bands along with the appearance of characteristic features between 800 and 1500 nm (Figure 1c). Such characteristic features between 800 and 1500 nm are also observed in **pNiDMP**,^33,40^ bearing mesityl substituents with occupied *ortho*-positions, suggesting a reduced extension of intramolecular dehydrogenative coupling for **pNiDCOOMePP** and **pNiDCNPP**. This observation is also in coalescence with our recent finding on **pNiDCOOMePh**, showing that porphyrin substitution with electron-withdrawing groups lowers the tendency of the aryl substituent to undergo intramolecular dehydrogenative coupling with the porphyrin macrocycle in these fused porphyrin-conjugated polymers.^42^

Interestingly, **pNiDCOOMePP** and **pNiDCNPP** are partially soluble in DCM (Figure 1a), while **pNiDOMePP**, **pNiDPP**, **pNiDTP**, and **pNiNapP** are shown to be fully insoluble in DCM. Such an observation is consistent with a reduced occurrence of intramolecular dehydrogenative coupling in **pNiDCOOMePP** and **pNiDCNPP**. The UV/vis analysis of the DCM-soluble fraction of **pNiDCOOMePP** and **pNiDCNPP** displays the absorption features identical to the ones of the oCVD thin films (Figures S2 and S3), confirming the formation of DCM-soluble oligomers (reduced intramolecular dehydrogenative coupling) from the oCVD reaction of 5,15-diaryl Ni(II) porphyrins bearing electron-withdrawing groups such as 4-methoxycarbonylphenyl (COOMePh) and 4-cyanophenyl (CNPh).

Irrespective of the studied aryl substituent, LDI-HRMS of oCVD thin films revealed the presence of different oligomers, up to tetrameric species, which correspond to the analytical limit of the instrument, i.e., 4000 *m*/*z* (Figures S4–S10). Additionally, peak distributions shifted from ca. 35 *m*/*z* are assigned to the chlorination (exchange of hydrogen atoms by chlorine atoms) of the fused porphyrin-conjugated polymers, which is clearly observed in all the oCVD thin films. Such side reaction is commonly observed when using chlorinated oxidants such as FeCl_3_ or CuCl_2_.^40−42^ No oligomeric/polymeric species are observed on the LDI-HRMS spectra of the corresponding reference sublimed porphyrin thin films (Figures S11–S17). The critical analysis of LDI-HRMS spectra in the dimeric region revealed information about the potential linkages between and within the porphyrin units, i.e., including the presence/absence of intramolecular dehydrogenative coupling. Interestingly, the dimeric region of the LDI-HRMS spectra of **pNiDPP**, **pNiDTP**, and **pNiDOMePP** exhibits peaks corresponding to M_2_-14H^+^ at *m*/*z* = 1022.09, *m*/*z* = 1078.16, and *m*/*z* = 1146.17, respectively (Figures S4–S6). The elimination of 7 hydrogen pairs implies the formation of fully unsaturated dimers, i.e., triply fused porphyrin dimers comprising 4 intramolecular dehydrogenative couplings, which correspond to the theoretical maximum. On the other hand, the dimeric region of LDI-HRMS spectra of **pNiDNapP** exhibits a maximum elimination of 10 hydrogen pairs (M_2_-20H^+^) with peaks at *m*/*z* = 1216.11, pointing toward increased intramolecular coupling as compared to other electron-donating counterparts (Figure S7). In contrast, in the fused porphyrin-conjugated polymers bearing electron-withdrawing substituents, i.e., **pNiDCOOMePP** (Figure S9) and **pNiDCNPP** (Figure S10), the signals related to the highest dehydrogenative coupling are observed at *m*/*z* = 1262.18 and *m*/*z* = 1130.14, respectively, which corresponds to M_2_-6H^+^ and points toward a lower extent of dehydrogenative coupling as compared to fused porphyrin-conjugated polymers bearing electron-donating substituents. The observed number of hydrogen pair losses in **pNiDCOOMePP** and **pNiDCNPP** is equivalent to the one observed in **pNiDMP**, i.e., three hydrogen pairs (*m*/*z* = 1198.34) (Figure S8), having a blocked *ortho*-position on the phenyl ring and therefore possessing no possibility of intramolecular dehydrogenative coupling. This confirms the absence of intramolecular dehydrogenative coupling in **pNiDCOOMePP** and **pNiDCNPP** and corroborates with the UV/vis/NIR observations of characteristic features in the case of **pNiDCOOMePP**, **pNiDCNPP**, and **pNiDMP** directing toward the formation of fused porphyrin-conjugated oligomers involving β–β/*meso*–*meso*/β–β triple bonding.

**Figure 2:**
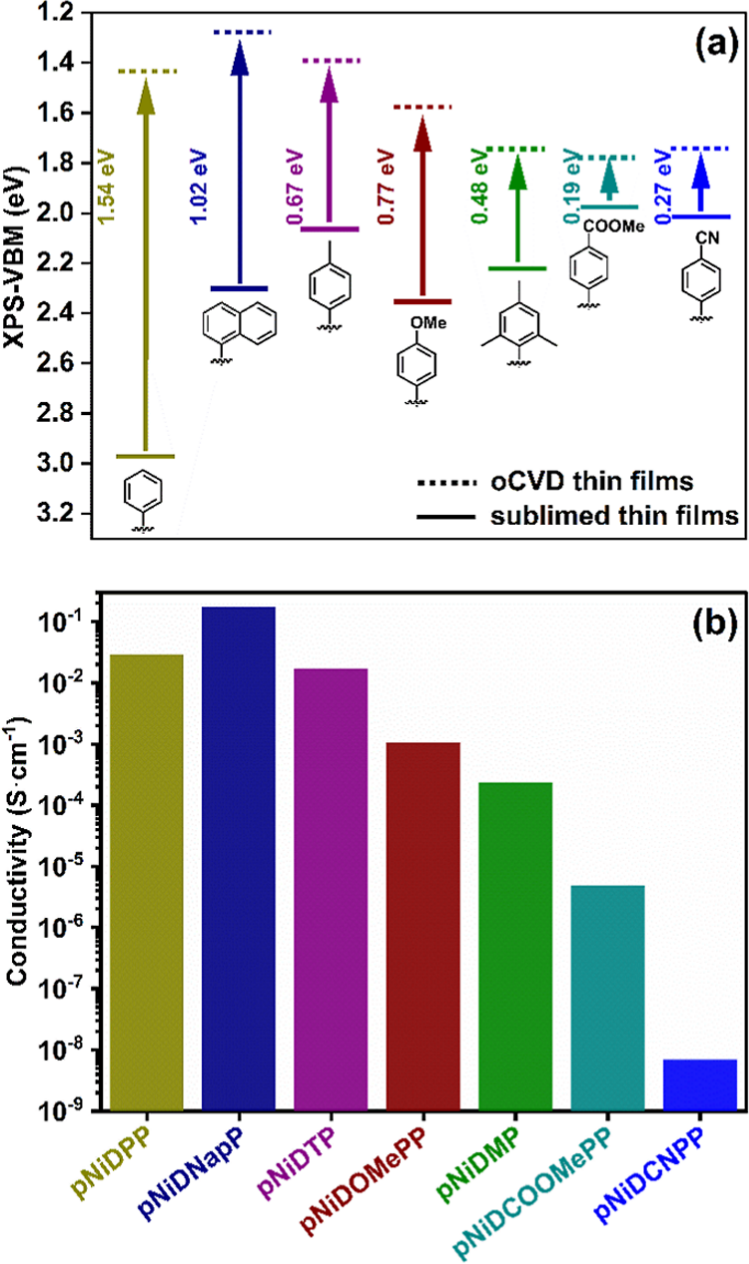
(a) Change in the VBM position of oCVD **pNiD(Aryl)P** (dotted line) thin films as compared to their reference sublimed porphyrins thin films (solid line). (b) Diagrammatic representation of the conductivity measured for the **pNiD(Aryl)P** thin films prepared from 5,15-diaryl Ni(II) porphyrins bearing different aryl substituents (from left to right): phenyl, naphthyl, tolyl, 4-methoxyphenyl, mesityl, 4-methoxycarbonylphenyl, and 4-cyanophenyl.

X-ray photoemission spectroscopy (XPS) shows that upon polymerization, all the oCVD thin films display a shift of their valence band maxima (VBM) toward lower binding energies in comparison to their respective reference sublimed porphyrin thin films (nonpolymerized) (Figures 2a and S18). This is in agreement with previous reports showing increase in the highest occupied molecular orbital (HOMO) due to increased π-electron delocalization owing to the formation of conjugated polymeric structures. Interestingly, the amplitude of the VBM shifts in the fused porphyrin-conjugated polymer thin films is greatly influenced by the nature of the substituent attached to the porphyrins. For example, the highest change in the VBM value is observed for **pNiDPP** with a shift of 1.54 eV, followed by **pNiDNapP** with a shift of 1.02 eV. However, **pNiDTP** and **pNiDOMePP** exhibit moderate shifts of their VBM, 0.67 and 0.77 eV with respect to reference sublimed porphyrin thin films, respectively. These shifts can be related to the electron-donating substituents which promote electronic delocalization in intramolecular dehydrogenative coupled fused porphyrin-conjugated polymers. Interestingly, mesityl-substituted **pNiDMP** (with no intramolecular dehydrogenative coupling) show a VBM shift of 0.48 eV. The VBM shift is further reduced to 0.19 and 0.27 eV for **pNiDCOOMePP** and **pNiDCNPP**, respectively, w.r.t. reference sublimed porphyrins thin films, suggesting that the porphyrins substituted with electron-withdrawing substituents not only afford polymers with reduced intramolecular dehydrogenative coupling but also with restricted electronic delocalization.

On the other hand, the band gap diagrams for the oCVD thin films calculated from the plots of the UV/vis/NIR absorption spectra and VBM values are shown in Figures S18–S20. **pNiDPP**, bearing unsubstituted phenyl groups, shows a band gap value of 2.32 eV, which indicates a strong electronic delocalization in this fused porphyrin-conjugated polymer. Interestingly, substitution of the porphyrins with electron-donating groups in **pNiDNapP**, **pNiDTP**, and **pNiDOMePP** results in the reduction of band gap values to 2.25, 2.25, and 2.16 eV, respectively, indicating improved electronic communication in the fused porphyrin-conjugated polymer thin films. In contrast, the band gap value increases for **pNiDMP** (2.71 eV) pointing toward reduced electron communication owing to the absence of intramolecular dehydrogenative coupling. Consistently, **pNiDCOOMePP** (2.53 eV) and **pNiDCNPP** (2.60 eV) containing electron-withdrawing substituents also exhibit larger band gap values, highlighting the reduced electronic delocalization due to the reduced or no intramolecular dehydrogenative coupling.

Furthermore, a strong influence of the aryl substituent on the conductivity of different oCVD thin films is evidenced (Figures 2b and S21). As stated above, the occurrence of the intramolecular dehydrogenative coupling induces the planarity in the molecules, thereby effecting electronic delocalization. The conductivity among different fused porphyrin-conjugated polymer thin films is as follows: **pNiDNapP** (1.76 × 10^–1^ S·cm^–1^) > **pNiDPP** (2.88 × 10^–2^ S·cm^–1^) > **pNiDTP** (1.73 × 10^–2^ S·cm^–1^) > **pNiDOMePP** (1.08 × 10^–3^ S·cm^–1^) > **pNiDMP** (2.34 × 10^–4^ S·cm^–1^) > **pNiDCOOMePP** (4.93 × 10^–6^ S·cm^–1^) > **pNiDCNPP** (6.90 × 10^–9^ S·cm^–1^). **pNiDNapP** exhibits a highest conductivity of 1.76 × 10^–1^ S·cm^–1^ among all the synthesized conjugated polymer thin films due to the greater extent of intramolecular dehydrogenative coupling, leading to flattening of molecular structure and increasing electronic delocalization. Similarly, **pNiDPP**, **pNiDTP**, and **pNiDOMePP** also exhibit considerable conductivity due to the presence of electron-donating moieties in addition to intramolecular dehydrogenative coupling. Besides, **pNiDMP** showed lower conductivity than the fused porphyrin-conjugated polymer thin films bearing electron-donating substituents due to the presence of the sterically crowded mesityl substituent responsible for both the absence of intramolecular dehydrogenative coupling reaction and reduced structural planarity resulting in limited electronic delocalization. Similarly, **pNiDCOOMePP** and **pNiCNPP** exhibit low conductivity due to the presence of electron-withdrawing groups, which reduce both intramolecular dehydrogenative coupling and the electronic delocalization in the fused porphyrin-conjugated polymer thin films. These findings are in good agreement with the UV\vis\NIR observations, as well as VBM and band gap calculations showing an increased electronic delocalization in the fused porphyrin-conjugated polymers substituted with electron-donating substituents as compared to sterically bulkier groups and electron-withdrawing groups.

**Figure 3:**
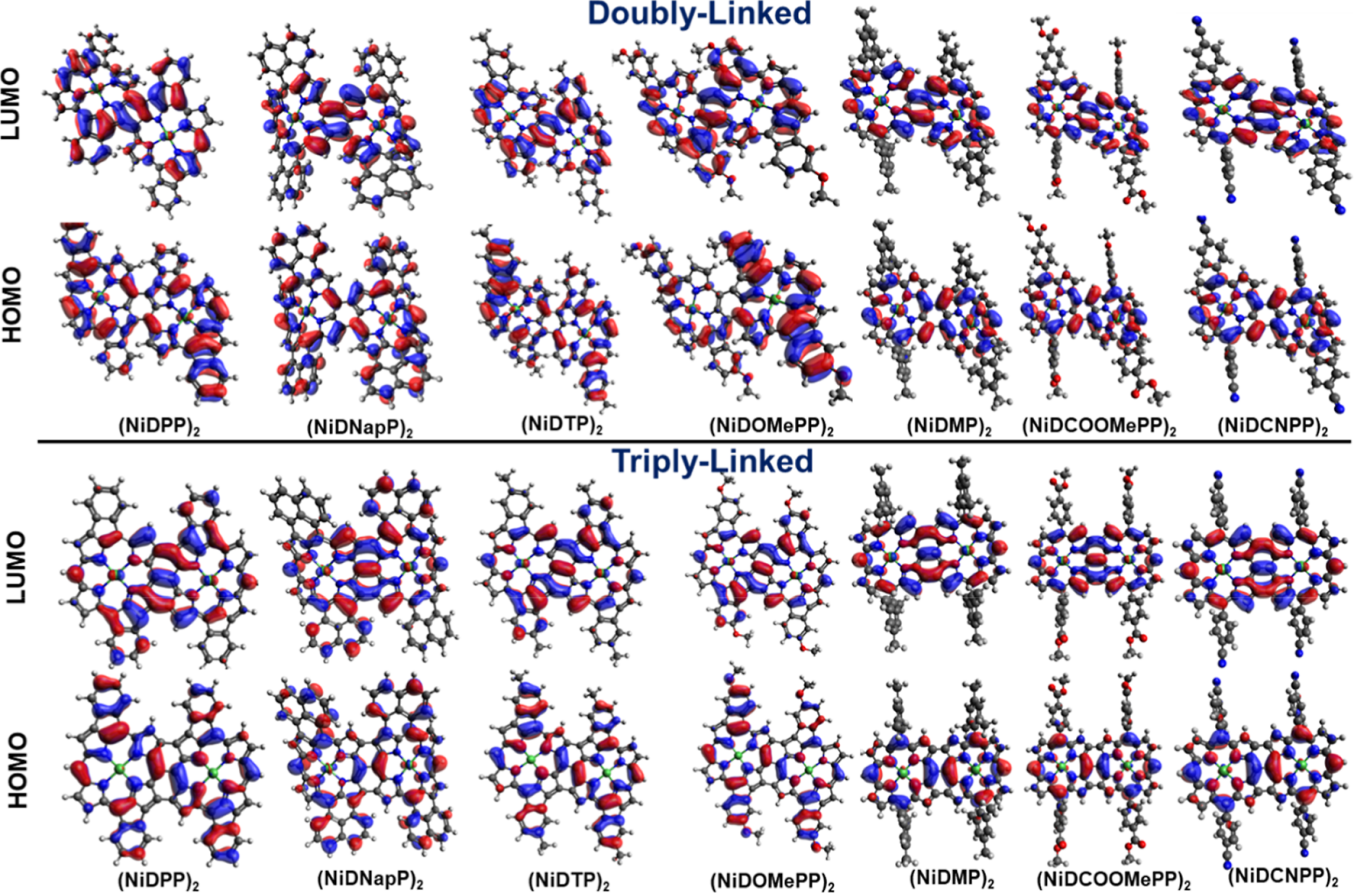
DFT-optimized structures showing the distribution of the FMOs of (top) doubly fused and (bottom) triply fused 5,15-diaryl Ni(II) porphyrin dimers bearing different aryl substituents (from left to right): phenyl, naphthyl, tolyl, 4-methoxyphenyl, mesityl, 4-methoxycarbonylphenyl, and 4-cyanophenyl.

Finally, to gain further insights into the electronic properties of the oCVD thin films, DFT calculations (BP86-RIJCOSX-D3BJ/def2-TZVP) were undertaken. As representative examples, dimeric structures having doubly and triply connected Ni(II) porphyrin units are optimized. As shown, FMOs are nicely distributed throughout the Ni(II) porphyrins via C–C linkage in both doubly and triply linked structures (Figure 3). The fused porphyrin-conjugated dimers bearing electron-donating substituents having intramolecular dehydrogenative coupling display an aryl group in plane to the Ni(II) porphyrin, whereas, in the case of (NiDCOOMePP)_2_, (NiDCNPP)_2_, and (NiDMP)_2_ where no intramolecular dehydrogenative coupling is anticipated, based on the investigation reported above, the aryl ring aligns nearly perpendicular to the plane of the Ni(II) porphyrin macrocycle. Moreover, the doubly linked dimers are observed to have a saddle-shaped molecular arrangement, whereas triply linked dimers show a more planar structure. These geometrical arrangements are in accordance with our previous reports.^42,44^ Interestingly, the occurrence of intramolecular dehydrogenative coupling affects the distribution of FMOs (HOMO and LUMO). In the case of (NiDPP)_2_, (NiDNapP)_2_, (NiDOMePP)_2_, and (NiDTP)_2_, the distribution of FMOs is extended to aryl substituents resulting in potentially increased electronic conjugation, whereas in the case of (NiDMP)_2_, (NiDCOOMePP)_2_, and (NiDCNPP)_2_, the aryl substituents do not contribute to the FMOs in the absence of intramolecular dehydrogenative coupling. Moreover, the presence of intramolecular cyclization induces flattening of molecular surface, leading to better π–π stacking, which in turn improves conductivity (Figure S22).^45^ These results further support our optoelectronic findings, showing the increased electronic communication in the fused porphyrin-conjugated polymers bearing electron-donating substituents (**pNiDNapP**, **pNiDPP**, **pNiDTP**, and **pNiDOMePP**) as compared to the fused porphyrin-conjugated polymers bearing electron-withdrawing substituents (**pNiDCOOMePP** and **pNiCNPP**).

### Electrical and Ammonia Sensing Properties of the BLH Devices

**Figure 4:**
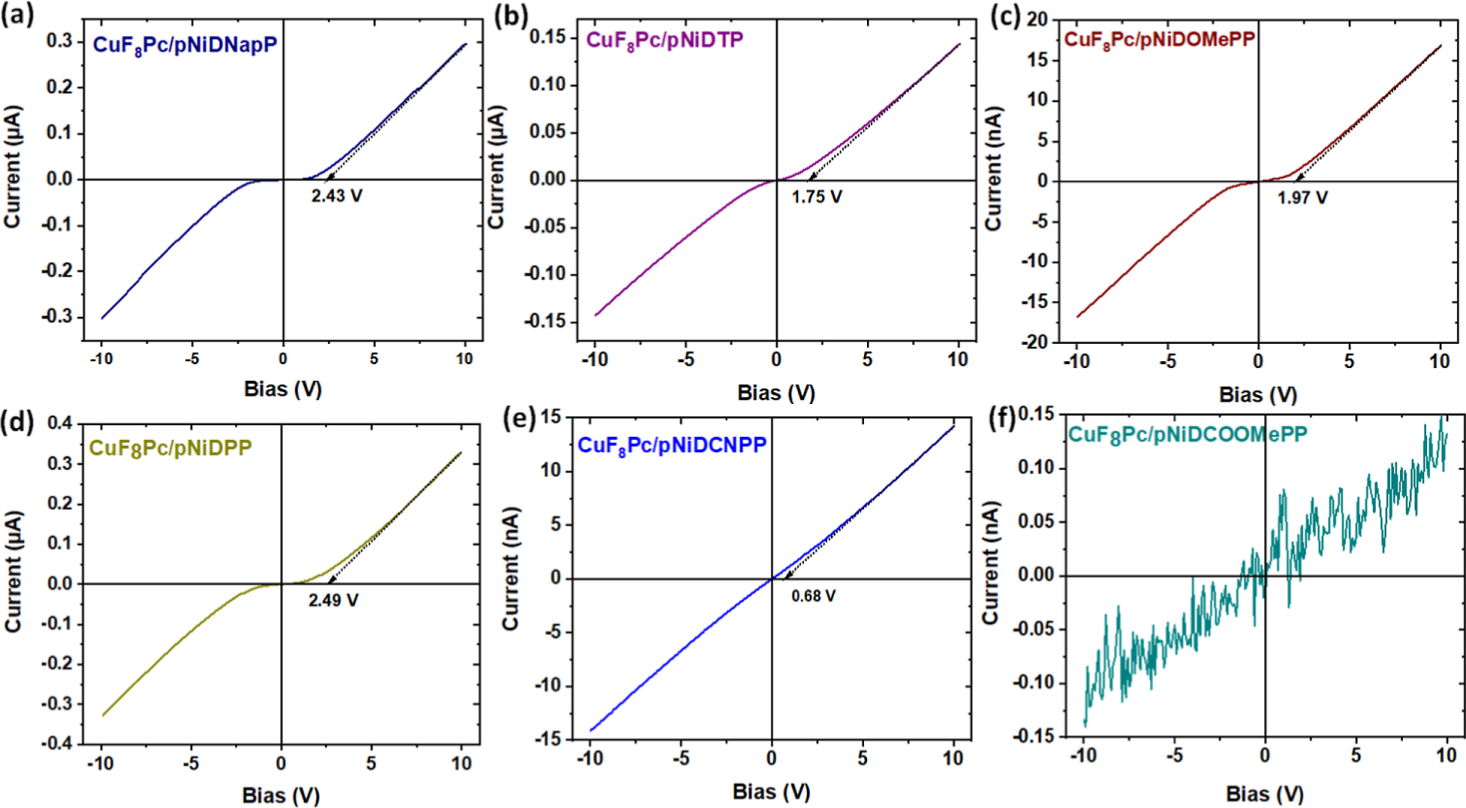
*I*–*V* characteristics, with an estimated apparent energy barrier, of the **CuF**_**8**_**Pc**/**pNiD(Aryl)P** BLH sensors prepared from 5,15-diaryl Ni(II) porphyrins bearing different aryl substituents, (a) naphthyl, (b) tolyl, (c) 4-methoxyphenyl, (d) phenyl, (e) 4-cyanophenyl, and (f) 4-methoxycarbonylphenyl.

Taking advantage of the ability of oCVD and the range of multiply fused Ni(II) porphyrin-conjugated polymers with contrasting electronic properties reported above, the polymeric films bearing electron-donating (**pNiDNapP**, **pNiDPP**, **pNiDTP**, and **pNiDOMePP**) and electron-withdrawing (**pNiDCOOMePP** and **pNiDCNPP**) aryl synthons were deposited onto a copper octafluoro-phthalocyanine **CuF**_**8**_**Pc** thin film to form BLH devices for gas sensing application. Electrical properties of the resulting BLH sensors were studied by performing *I*–*V* measurements in the applied voltage ranging from −10 V to +10 V. As expected for BLH devices, all the devices exhibit symmetric and nonlinear *I*–*V* curves, except the **CuF**_**8**_**Pc/pNiDCOOMePP** device, which shows huge noise, due to its very poor conducting nature (Figure 4). The typical nonlinear *I*–*V* curve observed in other organic BLH devices arises from the accumulation of mobile charges at the interface.^29,32^

This phenomenon is facilitated by the sublayer being highly resistive in comparison to the top layers.^39^ Consequently, charges injected from the electrode encounter significant resistance, leading to a lower current at a lower bias. However, as the bias increases, the injected charges gain sufficient energy to overcome the energy barrier, resulting in a higher current.^46^ Quantification of the nonlinearity in the *I*–*V* curve of the BLH device involves estimating the apparent energy barrier (*U*_th_) by drawing a tangent at high-voltage points on the *I*–*V* curves and extrapolating it to the *X*-axis.^33^ BLH devices based on **CuF**_**8**_**Pc/pNiDNapP** and **CuF**_**8**_**Pc/pNiDPP** exhibit higher *U*_th_ values of 2.43 and 2.49 V with similar current at +10 V (0.32 and 0.29 μA), respectively. **CuF**_**8**_**Pc/pNiDOMePP** (*U*_th_ = 1.97 V) and **CuF**_**8**_**Pc/pNiDTP** (*U*_th_ = 1.75 V) exhibit a slightly lower apparent energy barrier, while **CuF**_**8**_**Pc/pNiDCNPP** displays a significantly lower *U*_th_ value of 0.68 V.

**Figure 5:**
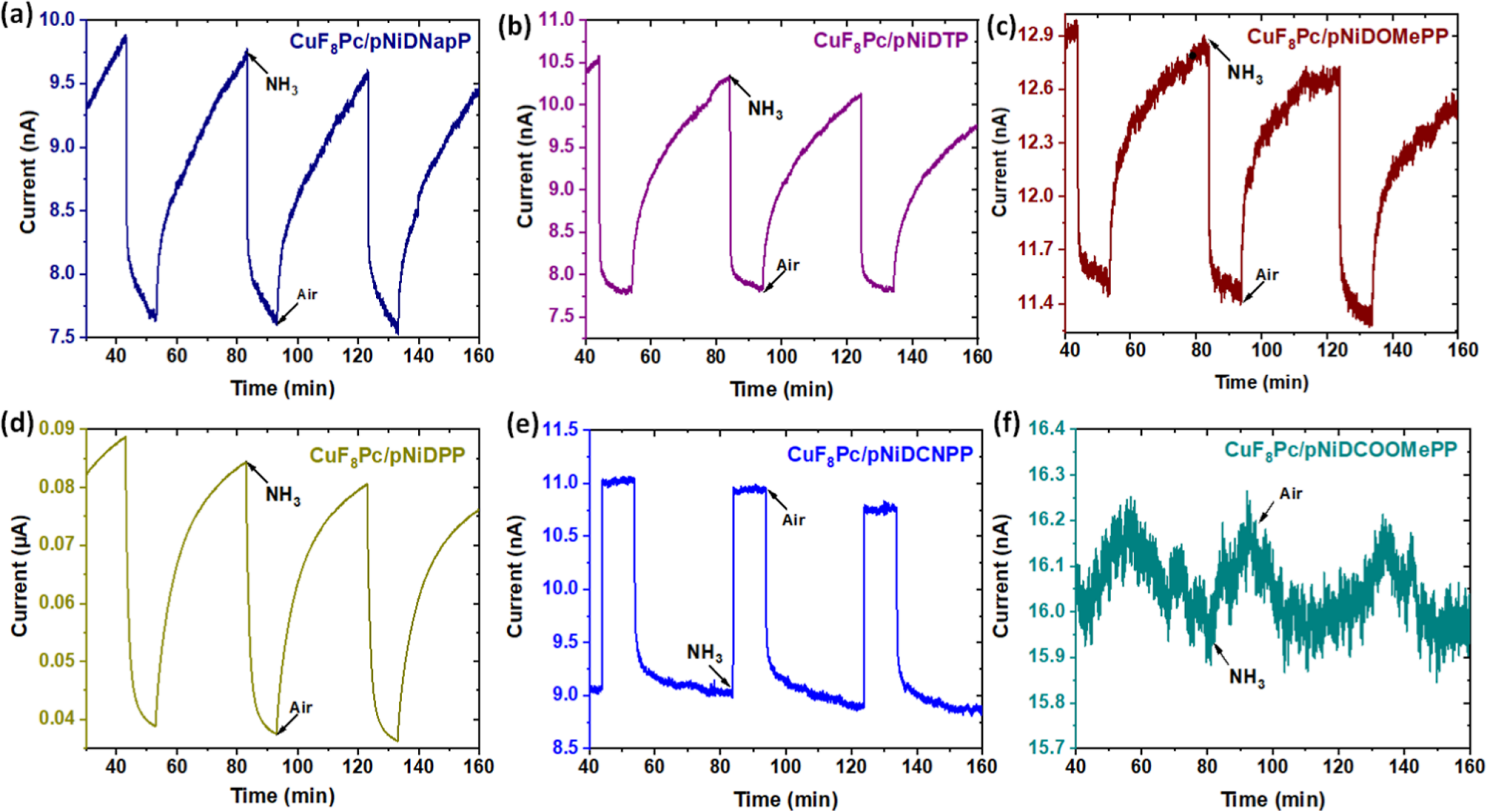
Response curves under successive exposures to 90 ppm ammonia for 10 min and recovery under air for 30 min at 45% relative humidity (RH) and room temperature (18–20 °C) for the **CuF**_**8**_**Pc**/**pNiD(Aryl)P** BLH sensors prepared from 5,15-diaryl Ni(II) porphyrins bearing different aryl substituents, (a) naphthyl, (b) tolyl, (c) 4-methoxyphenyl, (d) phenyl, (e) 4-cyanophenyl, and (f) 4-methoxycarbonylphenyl.

To investigate the sensing capabilities of the BLH devices toward ammonia, the devices were subjected to a high concentration of NH_3_ (90 ppm) for an extended exposure (10 min), followed by a recovery period of 30 min under a flow of air. Throughout the experiment, the relative humidity value is precisely maintained at 45% (ambient condition). The BLH devices engaged with the 5,15-diaryl Ni(II) porphyrin-conjugated polymer bearing electron-donating *meso*-substituents as the top layer (**pNiDNapP**, **pNiDTP**, **pNiDOMePP**, and **pNiDPP**) demonstrate a decrease in current when exposed to NH_3_, while an increase in current was observed during the recovery period (Figure 5a–d). Such behavior of BLH sensors indicates their p-type behavior, which is consistent with the electron-donating character of ammonia. Conversely, devices featuring the porphyrin polymer with electron-withdrawing substituents in the top layer (**pNiDCNPP** and **pNiDCOOMePP**) exhibit an increase in current under NH_3_ exposure, while a decrease in current during the recovery period (Figure 5e,f), showcasing their n-type nature.

In our previous studies, we extensively investigated the significant impact of the sublayer on device polarity.^47,48^ For the very first time, we have observed that the polarity of the device is influenced by the top layer. This effect primarily arises from the equilibrium between charge carriers in the sublayer. Previously, CuF_8_Pc was identified as an ambipolar molecule when combined with LuPc_2_ in the BLH device.^39,49^ In the current scenario, owing to the ambipolarity of the sublayer, the top layer grants the ability to engineer the polarity of the device according to the substituents present on the porphyrin macrocycle. Specifically, by leveraging the ambipolarity of the CuF_8_Pc sublayer, which can readily accept or donate electrons, the top layer defines the device’s polarity based on the substituents present on their macrocycle. While **pNiDCNPP** and **pNiCOOMePP** take electrons from the CuF_8_Pc sublayer displaying an n-type behavior, the opposite charge transfer occurs between the two layers with electron-donating substituted porphyrin-conjugated polymer thin films as top layers.

Notably, all the BLH sensors demonstrated a slight drift in baseline current, which can be attributed to the incomplete desorption of NH_3_ during the recovery period. However, the sensor signal was recovered by more than 95% during desorption period. The RR of the BLH sensors were calculated by using eq 1, where *I*_0_ and *I*_f_ are the initial and final current of the exposure period, respectively.
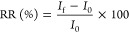
1

**CuF**_**8**_**Pc/pNiDPP** shows the highest negative RR of −56% with a response/recovery time (time taken to complete 90% of the final value) of 198/1170 s, respectively, whereas **CuF**_**8**_**Pc/pNiDCNPP** exhibits the highest positive RR of 22%, with a response/recovery time of 13/255 s, respectively. Notably, this device has the fastest response/recovery kinetics among all BLH sensors, which makes it highly suitable for application in emergency alert systems in chemical industries and laboratories (Table S4). The calculated values of RR and response/recovery time of BLH sensors are shown in Table 1.

**Table 1:** Comparison of RR, Res *t*_90_, and Rec *t*_90_ of **CuF**_**8**_**Pc**/**pNiD(Aryl)P** BLH Sensors

sensors	RR_[90ppm]_ (%)	response time (res *t*_90_) (s)	recovery time (rec *t*_90_) (s)
**CuF**_**8**_**Pc**/**pNiDNapP**	–23	293	1530
**CuF**_**8**_**Pc**/**pNiDTP**	–26	43	1383
**CuF**_**8**_**Pc**/**pNiDOMePP**	–11.5	64	1205
**CuF**_**8**_**Pc**/**pNiDPP**	–56	198	1170
**CuF**_**8**_**Pc**/**pNiCOOMePP**	2	519	1575
**CuF**_**8**_**Pc**/**pNiDCNPP**	22	13	255

**Figure 6:**
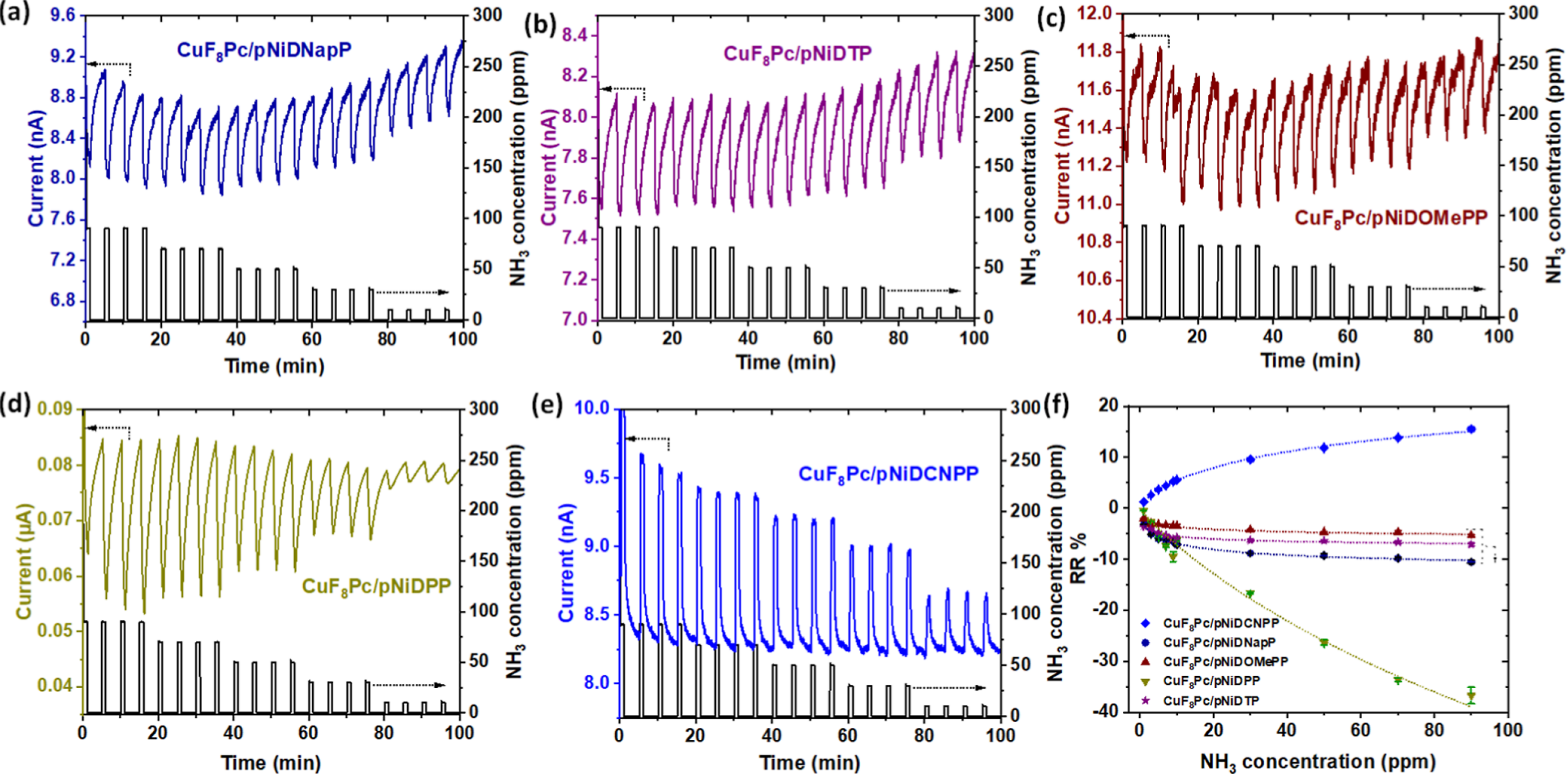
Response of under NH_3_ gas for 1 min and recovery under clean air for 4 min, in the range of NH_3_ concentration from 90 to 10 ppm, at 45% of RH and room temperature (18–20 °C) of the **CuF**_**8**_**Pc**/**pNiD(Aryl)P** BLH sensors prepared from 5,15-diaryl Ni(II) porphyrins bearing different aryl substituents: (a) naphthyl, (b) tolyl, (c) 4-methoxyphenyl, (d) phenyl, and (e) 4-cyanophenyl. (f) RR (calibration curve) of BLH sensors as a function of NH_3_ concentration.

With the exception of **CuF**_**8**_**Pc/pNiCOOMePP**, which exhibits high noise in long exposure, all other BLH sensors were further evaluated under a wide range of NH_3_ concentrations (90–10 ppm) with a dynamic cycle of 1 min exposure and 4 min recovery periods. Remarkably, the current variations observed in all sensors were highly reversible and repeatable throughout the experiments (Figure 6).

All of the BLH sensors displayed distinct gas responses down to 10 ppm. Notably, the **CuF**_**8**_**Pc/pNiDCNPP** BLH sensor demonstrated a sharp and stable response without any baseline drift. Hence, these sensors were further evaluated by exposure to low NH_3_ concentrations, ranging from 9 to 1 ppm. Most interestingly, **CuF**_**8**_**Pc/pNiDNapP**, **CuF**_**8**_**Pc/pNiDTP**, **CuF**_**8**_**Pc/pNiDOMePP**, and **CuF**_**8**_**Pc/pNiDCNPP** BLH sensors demonstrate observable responses even at 1 ppm experimentally (Figure S23a–c), and the **CuF**_**8**_**Pc/pNiDPP** BLH sensor exhibit distinct gas response down to 3 ppm (Figure S23d–e).

To understand the variation in sensor response influenced by different top layers, we calculated the RR using eq 1 and plotted them against varying NH_3_ concentration, creating calibration curves. Figure 6f illustrates these calibration curves for all five BLH sensors (a zoomed-in view is shown in Figure S24). Notably, all curves were fitted with Langmuir’s equation, indicating that the interaction between gas molecules and solid surfaces follows Langmuir-type adsorption. This suggests saturation of all available adsorption sites by NH_3_ gas molecules at higher concentrations. This effect is particularly evident in the **CuF**_**8**_**Pc/pNiDTP** BLH sensor, which almost reaches saturation between 30 and 70 ppm, displaying only a slight change in RR from −6.4 to −6.7%, respectively. Anyhow, it is important to note that this sensor still shows clear distinction even at low NH_3_ concentrations, ranging from 5 to 1 ppm, with RR values from −4.8 to −3.5%, respectively.

Determining the sensitivity (*S*) and limit of detection (LOD) of gas sensors is crucial.^50^ LOD was calculated for all five BLH devices using eq 2. Here, *N* is the noise of the sensor signal, *S* is the sensitivity given by the slope of the sensor calibration curve, and *I*_0_ is the sensor baseline current.
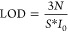
2

Among all BLH sensors, **CuF**_**8**_**Pc/pNiDPP** exhibits high sensitivity (−1.17% ppm^–1^), and **CuF**_**8**_**Pc/pNiDNapP** exhibits the lowest LOD (190 ppb). To comprehend, the sensitivity of the sensors was increased in the order of **CuF**_**8**_**Pc/pNiDTP** (−0.32% ppm^–1^) < **CuF**_**8**_**Pc/pNiDOMePP** (−0.52% ppm^–1^) < **CuF**_**8**_**Pc/pNiDCNPP** (−0.69% ppm^–1^) < **CuF**_**8**_**Pc/pNiDNapP** (−0.98% ppm^–1^) < **CuF**_**8**_**Pc/pNiDPP** (−1.17% ppm^–1^), and LOD decreased in the order of **CuF**_**8**_**Pc/pNiDPP** (2 ppm) > **pNiDOMePP** (508 ppb) > **CuF**_**8**_**Pc/pNiDTP** (492 ppb) > **CuF**_**8**_**Pc/pNiDCNPP** (199 ppb) > **CuF**_**8**_**Pc/pNiDNapP** (190 ppb). Estimated sensitivity and LOD of the BLH sensors are compiled in Table 2.

**Table 2:** Comparison of Sensitivities (*S*) and LOD of **CuF**_**8**_**Pc**/**pNiD(Aryl)P** BLH Sensors

devices	*S* (% ppm^–1^)	LOD (ppb)	[NH_3_] (ppm)
**CuF**_**8**_**Pc**/**pNiDNapP**	–0.98	190	3–1
**CuF**_**8**_**Pc**/**pNiDTP**	–0.32	492	9–1
**CuF**_**8**_**Pc**/**pNiDOMePP**	–0.52	508	3–1
**CuF**_**8**_**Pc**/**pNiDPP**	–1.17	2000	9–3
**CuF**_**8**_**Pc**/**pNiDCNPP**	0.69	199	3–1

All of these sensors displayed a LOD below 20 ppm, which is the daily exposure limit established by the European Parliament. Moreover, the **CuF**_**8**_**Pc/pNiDNapP** and **CuF**_**8**_**Pc/pNiDCNPP** BLH devices demonstrated a LOD below 200 ppb, making them exceptionally suitable for real environmental applications such as medical field, where concentrations of interest typically are in the sub ppm range.^51^

Further experiments involving other gases are currently in progress to determine the selectivity patterns of the developed BLH devices. It is well known that the response of such CGSs is related to the nature of the analyte. Specifically, for p-type devices, oxidizing gases will induce a positive response, while electron-donating gases will induce a negative response and vice versa in n-type devices. Consequently, the BLH devices developed herein are expected to be sensitive to strong oxidizing agents, such as nitrogen dioxide and ozone, followed by ammonia (as electron-donating gas). For other electron-donating species, the selectivity will also depend on the strength of the electron-donating character of the analyte.

## Conclusions

The growing need for sustainable and efficient CGSs has motivated the scientific community to exploit organic polymers as a promising alternative to metal-oxide based CGSs. Herein, we reported the direct synthesis of different 5,15-diaryl Ni(II) porphyrin-based conjugated polymers (**pNiD(Aryl)P**) bearing electron-donating (Ph, naphthyl, tolyl, OMePh, mesityl), or electron-withdrawing (COOMePh, CNPh) *meso*-substituents, and their integration, as a conductive top layer, on the **CuF**_**8**_**Pc** sublayer to construct the **CuF**_**8**_**Pc/pNiD(Aryl)P** BLH device. For the very first time, we demonstrated the change in sensing properties of BLH devices toward NH_3_ from p-type to n-type as a function of top layer. Detailed characterization of the conjugated polymer thin films revealed the influence of electronic properties of the *meso*-substituent on the dehydrogenative coupling of 5,15-diaryl Ni(II) porphyrins, resulting in the formation of conjugated polymers exhibiting different electronic properties. As per sensing studies, **CuF**_**8**_**Pc/pNiD(Aryl)P** BLH devices with electron-donating aryl groups as *meso*-substituents in a fused porphyrins display p-type behavior which changes to n-type on introduction of electron-withdrawing *meso*-substituents. All BLH devices, except **CuF**_**8**_**Pc/pNiDCOOMePP**, exhibit a nonlinear *I*/*V* curve owing to the formation of an energy barrier (*U*_th_) at the heterojunction interface as a result of work function difference between the porphyrin tape and the phthalocyanine with the highest *U*_th_ of 2.49 V in **CuF**_**8**_**Pc/pNiDPP** and the lowest of 0.68 V in **CuF**_**8**_**Pc/pNiDCNPP**. Most BLH devices exhibit highly reversible and clean responses toward NH_3_ with a slight drift in baseline owing to permanent accumulation of NH_3_ at some sites on the surface. Interestingly, **CuF**_**8**_**Pc/pNiDPP** show highest negative RR of −56% among all electron-donating materials and **CuF**_**8**_**Pc/pNiDCNPP** showing high positive RR of 22% for the materials with electron-withdrawing substituents. Importantly, among all the BLH devices, **CuF**_**8**_**Pc/pNiDPP** display a highest sensitivity of −1.17% ppm^–1^, whereas **CuF**_**8**_**Pc/pNiDCNPP** and **CuF**_**8**_**Pc/pNiDNapP** exhibit a LOD below 200 ppb. Moreover, the fast response/recovery time of 13 and 255 s for **CuF**_**8**_**Pc/pNiDCNPP** makes this device highly suitable for emergency applications in industries and laboratories. Finally, this work opens a large area of research for developing BLH devices combining different top and sublayers for the sensing of different oxidizing and reducing gases.

## Experimental Section

### Conjugated Polymer Thin-Film Preparation by oCVD

The oCVD reaction was performed in a custom-built oCVD reactor described in detail elsewhere.^40−44^ 5,15-Diphenyl Ni(II) porphyrin, 5,15-(ditolyl) Ni(II) porphyrin, 5,15-(dinaphthyl) Ni(II) porphyrin, 5,15-(di-4-methoxyphenyl) Ni(II) porphyrin, 5,15-(dimesityl) Ni(II) porphyrin, 5,15-(di-4-methoxycarbonylphenyl) Ni(II) porphyrin, and 5,15-(di-4-cyanophenyl) Ni(II) porphyrin, were obtained from PorphyChem (98%) and were used without further purification. Based on previous reports,^40−44^ iron(III) chloride (97%, Sigma-Aldrich) was chosen as the oxidant. Table S1 summarizes the deposition conditions used for each porphyrin investigated. The temperature used to sublime the oxidant was 170 °C in all cases. Glass microscope slides (Menzel-Gläser Superfrost), silicon wafers (Siegert Wafer), interdigitated chips (OFET Gen4, Fraunhofer), and indium tin oxide (ITO) (coated with CuF_8_Pc) were used as substrates for further characterizations. Prior deposition, all the substrates were cleaned with absolute ethanol (99.98%, VWR chemicals) and dried with nitrogen gas. For all the depositions, the substrate holder was initially kept at 150 °C at a pressure of 10^–3^ mbar [under an argon (99.999%, Air Liquide) atmosphere] for 30 min. After that, the temperature of the substrate and pressure inside the oCVD reactor was increased to 200 °C and 10^–4^ mbar, respectively, for another 1 h to remove unreacted FeCl_3_ (see the Supporting Information for detailed reaction mechanism, Schemes S1 and S2). Additionally, reference sublimed porphyrin thin films (in the absence of the oxidant) were obtained under the same conditions used for oCVD films for initial 30 min.

### Preparation of the CuF_8_Pc Thin Film and BLH Devices

50 nm-thick CuF_8_Pc was deposited as a common sublayer on ITO-interdigitated electrodes (IDEs) lithographically patterned on glass substrates (1 cm^2^). The IDE consisted of 16 pairs of ITO digits, having a width and spacing between two digits of 75 μm. Prior to the deposition, electrodes were properly cleaned by following the procedure reported previously.^52^ The sublayer was coated on the IDE by thermal evaporation in a UNIVEX 250 thermal evaporator under secondary vacuum (ca. 10^–7^ mbar). To fabricate the BLH devices, ∼50 nm-thick different 5,15-diaryl Ni(II) porphyrin-conjugated polymers were deposited on top of CuF_8_Pc films, using the oCVD technique (Scheme S3).

### Thin-Film Characterization

The UV/vis/NIR spectra of the reference sublimed porphyrin thin films and oCVD thin films deposited on glass slides were recorded with a PerkinElmer Lambda 1050 spectrometer, in the transmission (*T*) mode, in the 300–2500 nm wavelength interval. The absorbance (*A*) was calculated as *A* = −log(*T*). From the absorbance spectra, the direct optical band gap of the fused porphyrin-conjugated polymer thin films was estimated through the Tauc plot as (α*h*ν)^1/*n*^ = *A*(*h*ν – *E*_g_), where α is the absorbance coefficient, *n* = 1/2 for direct transitions, *h* is the Planck’s constant, and ν the wavelength number. The absorbance coefficient was calculated as α = ln(10)*A*/*l*, where *l* is the film’s thickness. The thin-films thicknesses were measured using a KLA-Tencor P-17 Stylus Profiler. Additionally, the thin films were rinsed with dichloromethane (HPLC grade >99.8%, SupraSolv) for comparison with the as-deposited films. XPS measurements were performed with a Kratos Axis Ultra DLD instrument using a monochromatic Al Kα X-ray source of energy 1486.6 eV at 105 W power. Charge calibration was accomplished by fixing the binding energy of carbon (C 1s) to 285.0 eV.

Laser desorption/ionization high-resolution mass spectrometry (LDI-HRMS) measurements were performed using an AP-MALDI UHR ion source (MassTech, Inc.) coupled to an LTQ/Orbitrap Elite (Thermo Scientific), as described in detail elsewhere.^40−44^ In-source fragmentation (*E* = 70 V) was used to prevent the formation of clusters. The measurements were performed on Si wafer substrates coated with either the reference sublimed porphyrin thin films or oCVD thin films, which were directly placed on the sample holder.

All fundamental electrical and sensing properties of the BLH were performed using a Keithley 6517B electrometer controller by homemade software. Current–voltage (*I*–*V*) characteristics were registered with voltage values ranging from −10 V to +10 V in steps of 0.1 V. Sensing measurements were conducted in ambient conditions (45% RH at 19 °C) with an applied bias of +3 V. Except for the **pNiDCOOMePP**-based device, we applied +10 V due to its very poor conducting nature.

### Sensing Experiments

Gas cylinders of dry synthetic air and dry NH_3_ containing synthetic air at concentrations of 98 and 985 ppm (mol/mol) were purchased from Air Liquide. The volume of the test chamber was 8 cm^3^, and the total flow rate was set to 550 mL·min^–1^ by mass flow controllers (Brooks Instrument) connected to homemade LabView software. 10 min/30 min and 1 min/4 min exposure/recovery cycles were controlled by switching electronic valves. Details of the test bench setup were exactly the same as in our previous work (Scheme S4).^53,54^

### DFT Calculations

All the structures were optimized using Orca version 5.0.1.^55−57^ The DFT calculations were performed using the BP86^58,59^ functional with the Karlsruhe valence triple-ζ basis set “def2-TZVP”^60−62^ and Weigend’s auxiliary basis set.^63^ Dispersion effects were considered by Grimme approximation “D3”.^64,65^ To simply speed up the iteration, RIJCOSX approximation is included.^66,67^ The optimized geometries were confirmed by attaining local minima by confirming the absence of negative frequencies after numerical frequency analysis.

## Supporting Information Available

Schematic representation of the oxidative polymerization reaction mechanism of 5,15-diaryl Ni(II) porphyrins; diagrammatic representations of BLH device fabrication and workbench for gas sensing; UV/vis/NIR absorption, LDI-HRMS, and XPS results of all the sublimed and oCVD 5,15-diaryl Ni(II) porphyrin thin films; band diagram, Tauc’s plot, conductivity measurements for the oCVD 5,15-diaryl Ni(II) porphyrin thin films; molecular simulations for [NiDPP]_2_, [NiDCNPP]_2_, and [NiDMP]_2_ dimeric aggregates; gas sensing plots for the reported BLH devices at low NH_3_ concentrations; and coordinates for the simulated structures (PDF)
