# Supplementary material for: Engineering Nickel(II) Porphyrin-Conjugated Polymers with Different Aryl meso-Substituents for n-Type and p-Type Ammonia Sensors
Source: ACS Appl Mater Interfaces. 2024 Nov 25;16(49):68091–102. doi: 10.1021/acsami.4c15731 (PMC11647756; doi:10.1021/acsami.4c15731)
Supplement: Supplementary file 1 — am4c15731_si_001.pdf [file am4c15731_si_001.pdf]

Supporting Information

Engineering Nickel(II) Porphyrin Conjugated Polymers with Different Aryl meso-Substituents for n-Type and p-Type Ammonia Sensors

Deepak Bansal[†, a], Sujithkumar Ganesh Moorthy[†, b], Marcel Bouvet[*, b], Nicolas D. Boscher[*, a]

[a]Luxembourg Institute of Science and Technology (LIST), 28 Avenue des Hauts-Fourneaux, L-4362 Esch-sur-Alzette, Luxembourg.

E-mail: nicolas.boscher@list.lu

[b]Institut de Chimie Moléculaire de l'Université de Bourgogne (ICMUB), UMR CNRS 6302, Université de Bourgogne, 9 Avenue Alain Savary, 21078 Dijon cedex, France.

E-mail: marcel.bouvet@u-bourgogne.fr

† Contributed equally

* Corresponding authors

Content	Page no.
**Scheme S1**. Schematic representation of the reaction mechanism for the formation of Ni(II)porphyrin polymer…..	S3
**Scheme S2.** Schematic drawing of the orbitals' relative energy, radical cation character and oxidation potential of the nickel (5,15 di phenyl porphyrins) during the formation of polymer……………………………………………….	S4
**Scheme S3**. Diagrammatic representation for the fabrication of bilayer heterojunction device………………………	S4
**Scheme S4**. Schematic representation of work bench used for gas sensing experiments…………………………….	S5
**Fig. S1** UV/Vis/NIR absorption spectra of the reference sublimed porphyrins thin films….………………………..	S5
**Fig. S2** Comparative UV/Vis/NIR absorption spectra of reference sublimed porphyrins thin films, and the oCVD **pNiD(Aryl)P** thin films, as-deposited and after rinsing with dichloromethane……………………………………..	S6
**Fig. S3** Comparative UV/Vis/NIR absorption spectra of DCM washings from the reference sublimed porphyrins thin films and the oCVD **pNiD(Aryl)P** thin films…………………..………………………………………………….	S7
**Fig. S4** LDI-HRMS spectra of **pNiDPP**………………………………………………………………………………	S9
**Fig. S5** LDI-HRMS spectra of **pNiDTP**………………………………………………………………………………	S10
**Fig. S6** LDI-HRMS spectra of **pNiDOMePP**………………………………………...……………………………	S10
**Fig. S7** LDI-HRMS spectra of **pNiDNapP**…………………………………………...…………………………	S11
**Fig. S8** LDI-HRMS spectra of **pNiDMP**……………………………………………………………………………..	S11
**Fig. S9** LDI-HRMS spectra of **pNiDCOOMePP**…………………………………...………………………….	S12
**Fig. S10** LDI-HRMS spectra of **pNiDCNPP**………………………………………………………………………..	S12
**Fig. S11** Comparative LDI-HRMS spectra for **pNiDPP**…………………………………………………………….	S13
**Fig. S12** Comparative LDI-HRMS spectra for **pNiDNapP**………………………………………………………….	S14
**Fig. S13** Comparative LDI-HRMS spectra for **pNiDTP**…………………………………………………………….	S15
**Fig. S14** Comparative LDI-HRMS spectra for **pNiDOMePP** ………………………………………………………	S16
**Fig. S15** Comparative LDI-HRMS spectra for **pNiDMP** …………………………………….............................	S17
**Fig. S16** Comparative LDI-HRMS spectra for **pNiDCOOMePP**…………………………………………………..	S18
**Fig. S17** Comparative LDI-HRMS spectra for **pNiDCNPP**…………………………………………………………	S19
**Fig. S18** Valence band minimum energy (VBM) measurements..…………………………………………………..	S20
**Fig. S19** Band gap diagram of the oCVD **pNiD(Aryl)P** thin films…………….………….…..........................	S21
**Fig. S20** Energy band gap value estimation of the oCVD **pNiD(Aryl)P** thin films using Tauc's plot………………	S22
**Fig. S21** 2-point conductivity plots for the oCVD **pNiD(Aryl)P** thin films………………………………………….	S23
**Fig. S22** DFT optimization of dimeric aggregates……………………………………………………………………	S24
**Fig. S23** Response of **CuF8Pc/pNiD(Aryl)P** bilayer heterojunction sensors under NH_3 gas……………………….	S24
**Fig. S24** Relative response (calibration curve) of **CuF8Pc/pNiD(Aryl)P** bilayer heterojunction sensors as a function of NH_3.…………………………………...……………………….………………………………………………	S25
**Table S1.** Details of oCVD deposition conditions…………………………..……………………………………..	S8
**Table S2**. Thickness of the oCVD **pNiD(Aryl)P** thin films …………………………………………………………	S9
**Table S3**. Behaviour (n-type or p-type) of **CuF8Pc/pNiD(Aryl)P** BLH sensors upon NH_3 exposure………………	S25
**Table S4**. Comparative analysis of previously reported bilayer heterojunction devices for NH_3 sensing……………	S26
**Table S5-S17**. Coordinates for triply and doubly fused porphyrins dimers…………………………………………..	S27-76

Proposed mechanism for polymerization of Ni(DAryl)porphyrin:

The details of reaction mechanism for dehydrogenative coupling in metalloporphyrin is still debated. The pioneer work by Osuka *et* al.[1] reported that porphyrin is oxidized to form a π-radical cation (electron-deficient/electrophile) in presence of an oxidant which can readily react with a neutral porphyrin (electron-rich/nucleophile) yielding intermediate adducts (figure 1).[1,2,3] A second oxidation and the elimination of two H^+ from the intermediate yields the products of the reaction i.e. meso-meso, β-meso, or β–β coupled porphyrins. Further oxidation and deprotonation of the dimer consecutively yields doubly and triply linked porphyrins. The reaction mechanism highlights the importance of the first C-C formation, which dictates the regioselectivity of the overall coupling reaction.

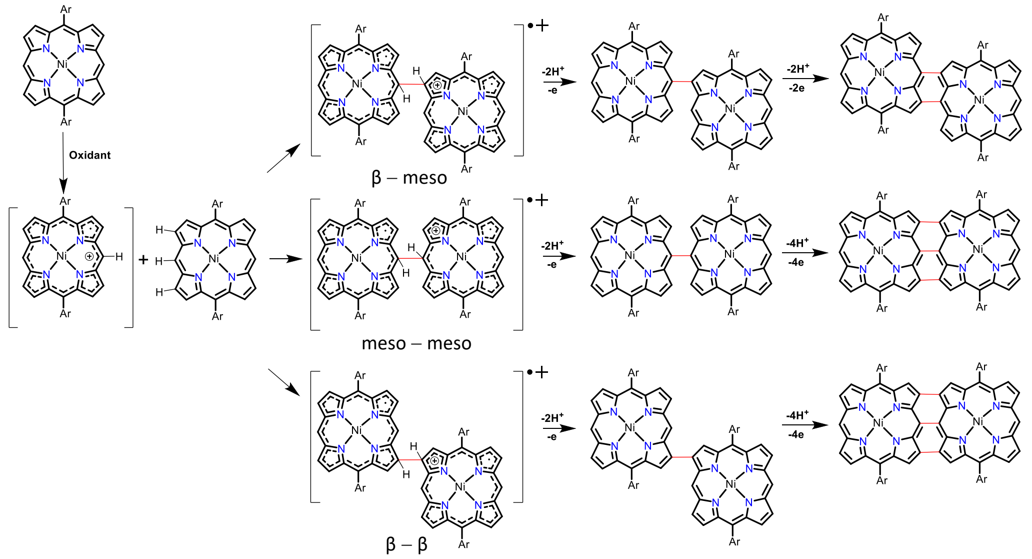

Scheme S1. Schematic representation of the reaction mechanism for the formation of Ni(II)porphyrin polymer.

Importantly, the regioselectivity between the doubly or triply linked porphyrins highly depends on the central metal ion and on the substituent pattern.[4–7] Osuka reported the high regioselectivity of nickel(II) di-phenyl porphyrins towards the formation of doubly linked porphyrin tapes.[2] Similarly, tri-phenyl palladium(II) porphyrins, and porphyrins bearing strong electron-with drawing groups on the meso position form mainly doubly linked porphyrin tapes.[5] In almost all cases, the meso-position is involved in the oxidative coupling. This is attributed to the high electron density at the meso-position of the neutral porphyrin, which acts as nucleophile in the coupling reaction. [1,8-10] The significant electron density is related to the phenyl rings at the meso positions acting as electron donors and thereby raising the a_{2u} orbital energy level compared to the a_{1u} orbital (Figure 2). The a_{2u} orbital possesses significant orbital coefficient at the meso-positions and a node at the β−positions. As a result, in porphyrins with a_{2u} orbital higher than a1u, the meso-positions possess nucleophilic behaviour. Since higher energy a_{2u} orbitals are typical for neutral 5,15-di-(aryl) porphyrins, regioselectivity in oxidative coupling reactions is associated with the electrophilic site after the formation of the radical cation. [1, 8-10]

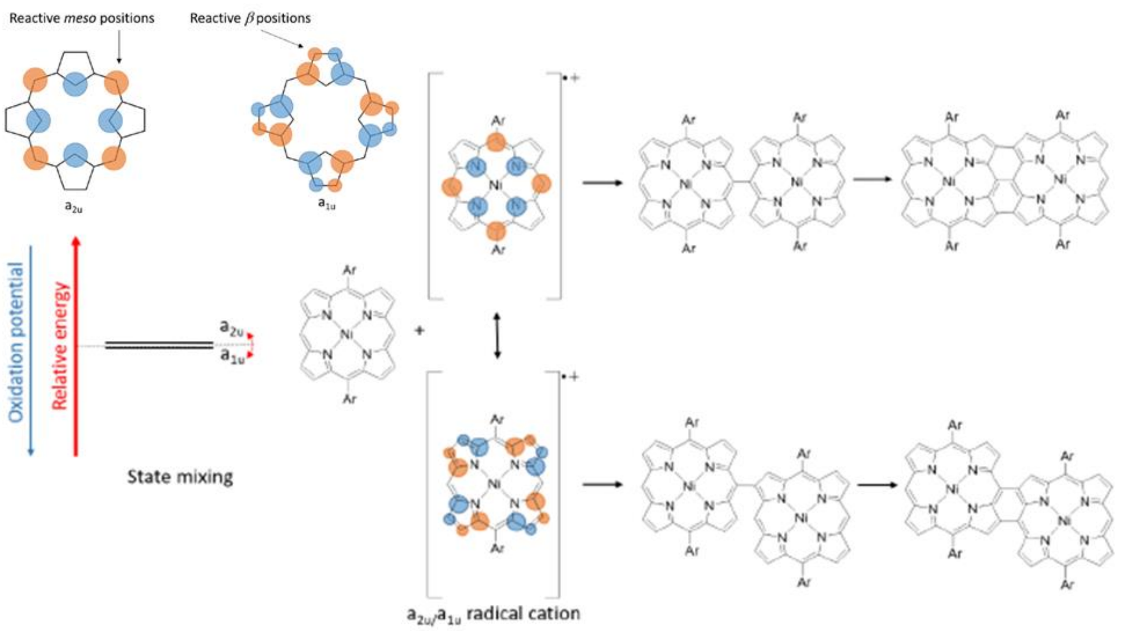

Scheme S2. Schematic drawing of the orbitals' relative energy, radical cation character and oxidation potential of Ni(II) *5,15*-di-phenyl porphyrin during its oxidative polymerization.

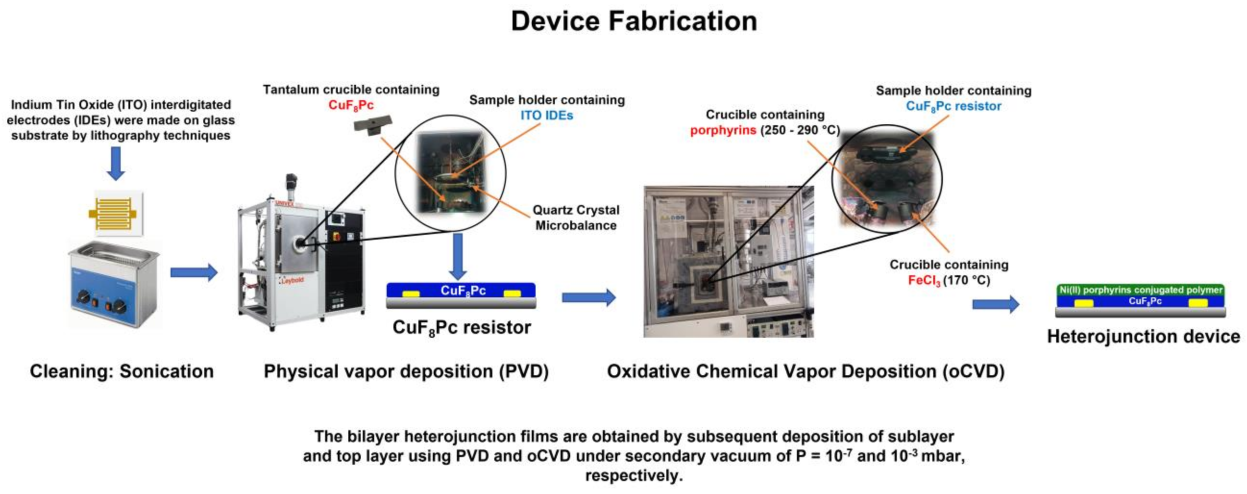

Scheme S3. Diagrammatic representation for the fabrication of bilayer heterojunction devices.

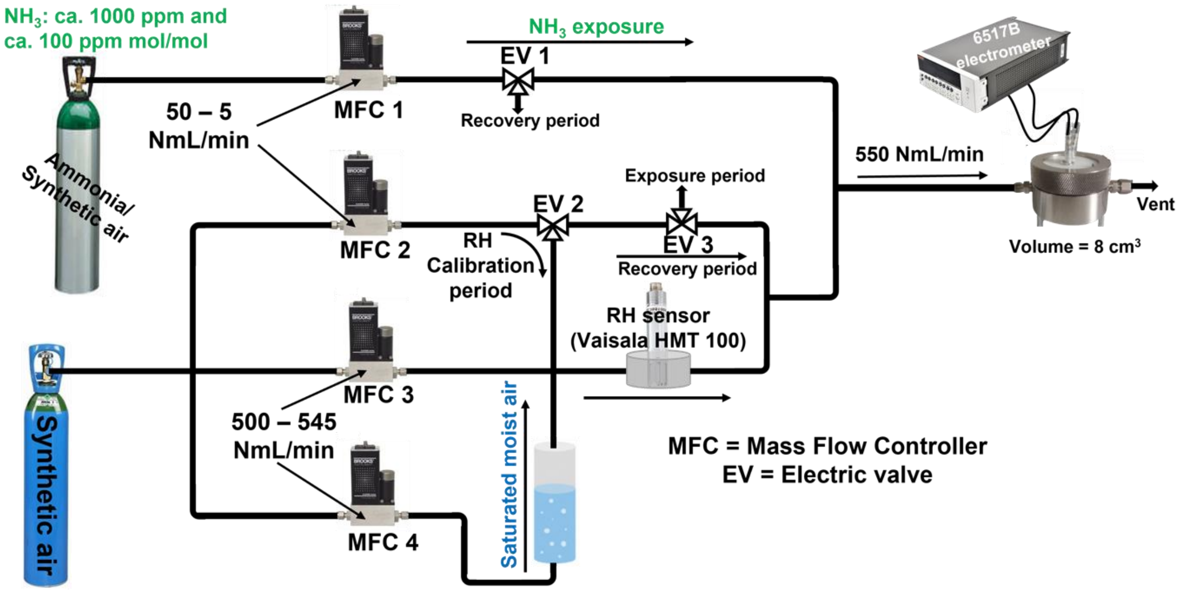

Scheme S4. Schematic representation of work bench used for gas sensing experiments.

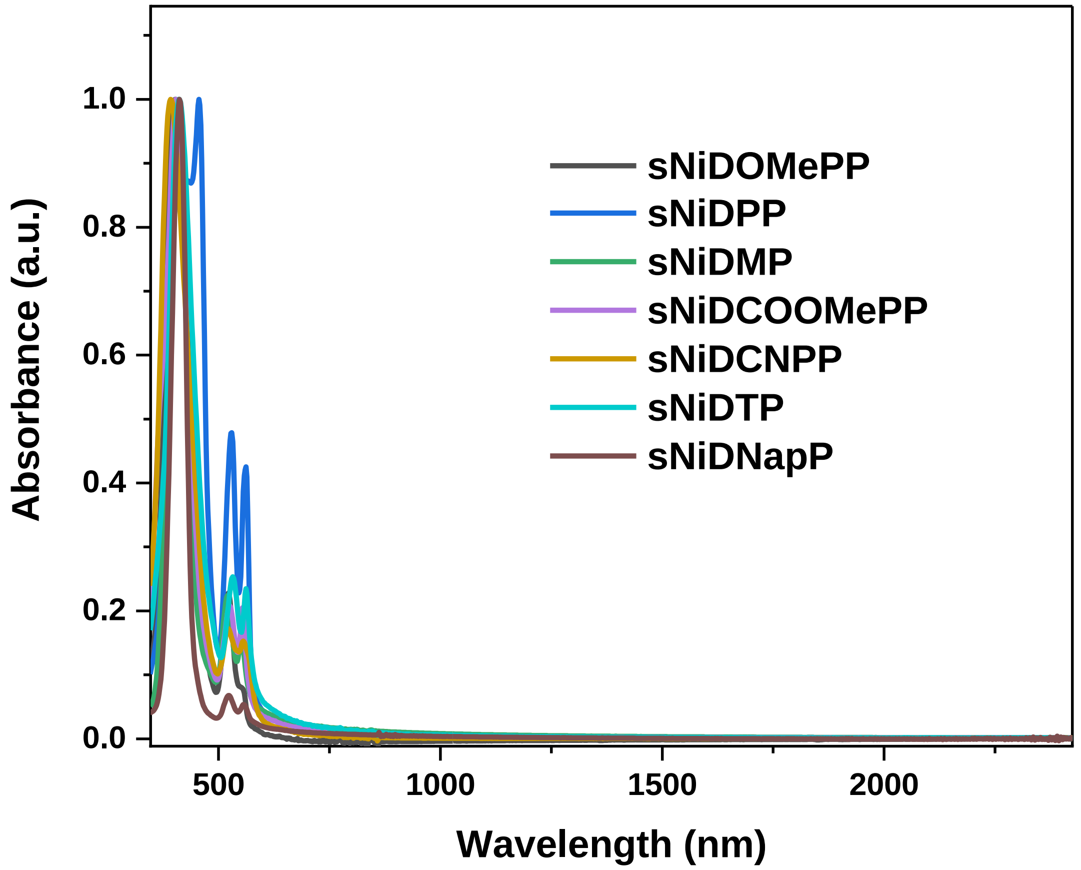

Figure S1. The UV/Vis/NIR absorption spectra of the reference sublimed porphyrins thin films (prepared in the absence of oxidant).

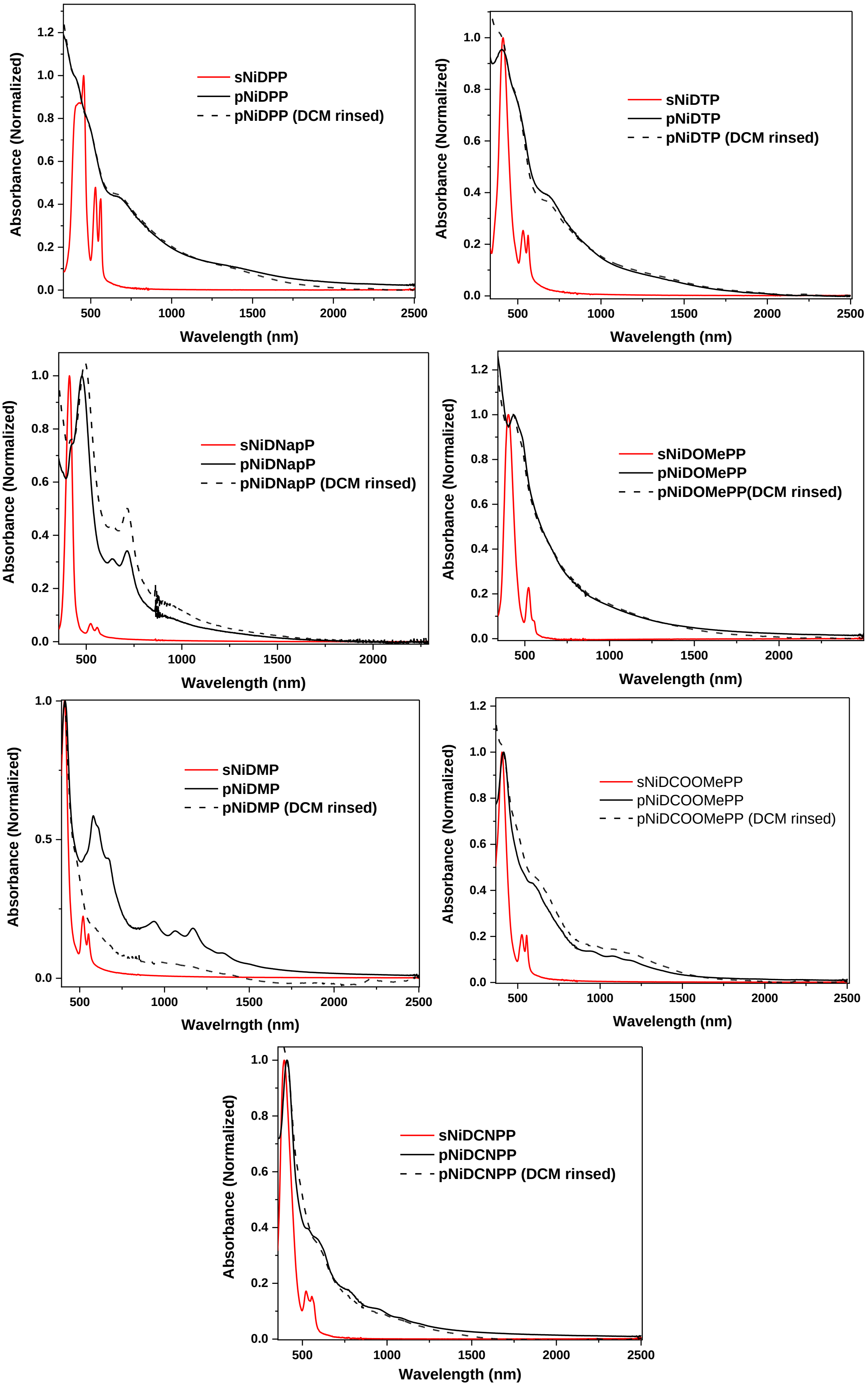

Figure S2. Comparative UV/Vis/NIR absorption spectra of the reference sublimed porphyrins thin films (red solid line) and oCVD **pNiD(Aryl)P** thin films before (black solid line) and after rinsing with DCM (black dashed line).

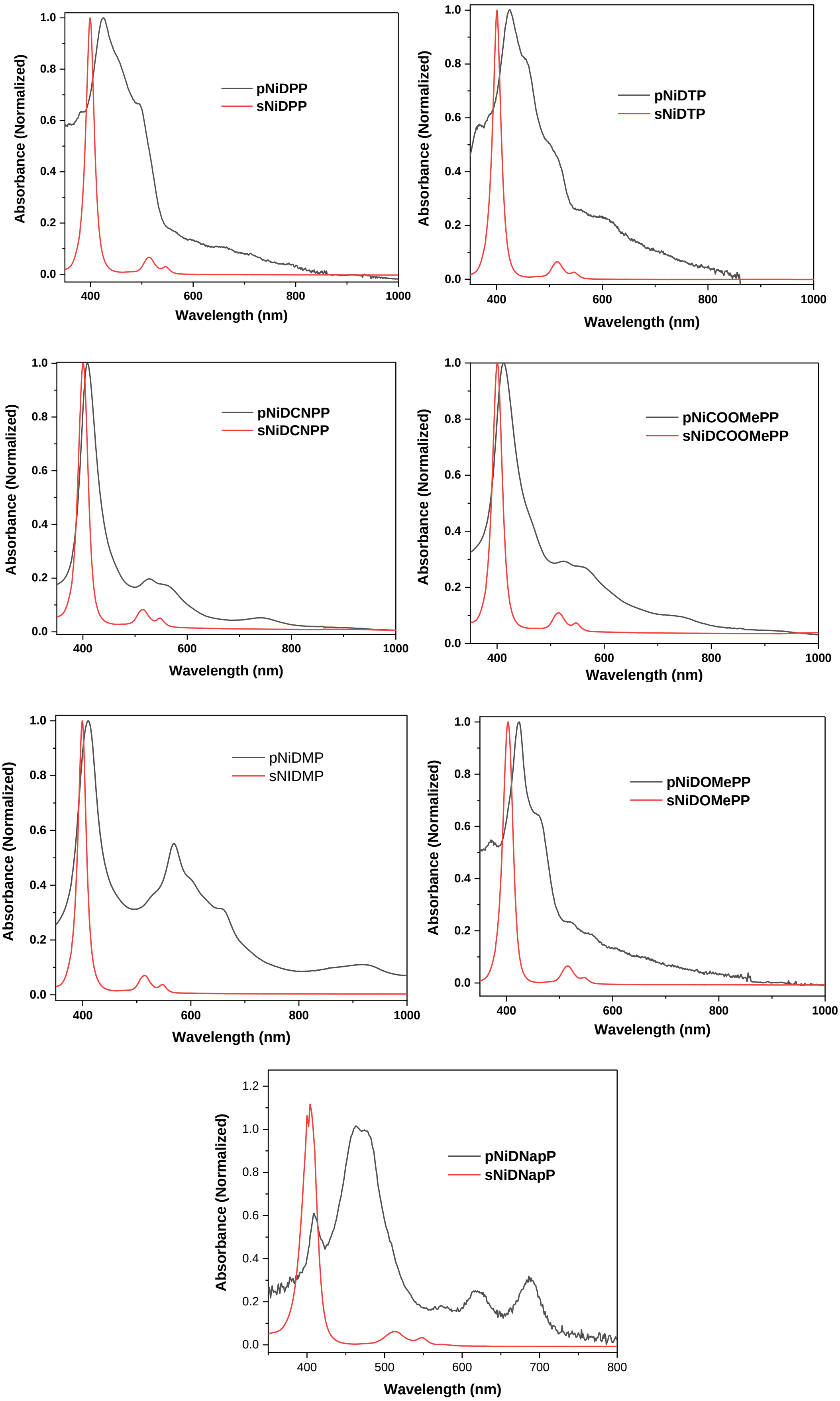

Figure S3. Comparative UV/Vis/NIR absorption spectra of DCM washings obtained from reference sublimed porphyrins thin films (red solid line) and oCVD **pNiD(Aryl)P** thin films (black solid line).

Table S1. Porphyrin name, chemical formula, molecular weight, sublimation temperature and sublimed amount used for the preparation of all the reported oCVD **pNiD(Aryl)P** thin films. Substrate temperature was 150°C and working pressure was 10^{-3} mbar.

**pNiDPP** from Nickel(II) 5,15-(diphenyl)porphyrin	
Chemical Formula	$C_{32}H_{20}N_4Ni$
Molecular Weight	519.23 g·mol^{-1}
Sublimed Temperature	235°C
Sublimed Amount	12.8 mg
**pNiDNapP** from Nickel(II) 5,15-(di-naphthyl)porphyrin	
Chemical Formula	$C_{40}H_{24}N_4Ni$
Molecular Weight	618.13 g·mol^{-1}
Sublimed Temperature	310°C
Sublimed Amount	11.0 mg
**pNiDTP** from Nickel(II) *5,15*-(di-tolyl)porphyrin	
Chemical Formula	$C_{34}H_{24}N_4Ni$
Molecular Weight	546.13 g·mol^{-1}
Sublimed Temperature	255°C
Sublimed Amount	11.3
**pNiDOMePP** from Nickel(II) 5,15-(di-4-methoxyphenyl)porphyrin	
Chemical Formula	$C_{34}H_{24}N_4NiO_2$
Molecular Weight	578.12 g·mol^{-1}
Sublimed Temperature	285°C
Sublimed Amount	11.5 mg
**pNiDMP** from Nickel(II) 5,15-(di-mesityl)porphyrin	
Chemical Formula	$C_{38}H_{32}N_4Ni$
Molecular Weight	602.19 g·mol^{-1}
Sublimed Temperature	240°C
Sublimed Amount	12.0 mg
**pNiDCOOMePP** from Nickel(II) 5,15-(di-4-methoxycarbonylphenyl)porphyrin	
Chemical Formula	$C_{36}H_{24}N_4NiO_4$
Molecular Weight	635.305 g·mol^{-1}
Sublimed Temperature	290°C
Sublimed Amount	13.7 mg
**pNiDCNPP** from Nickel(II) 5,15-(di-4-cyanophenyl)porphyrin	
Chemical Formula	$C_{34}H_{18}N_6Ni$
Molecular Weight	568.09 g·mol^{-1}
Sublimed Temperature	310°C
Sublimed Amount	12.0 mg
Iron(III) chloride ($FeCl_3$)	
Chemical Formula	Cl_3Fe
Molecular Weight	162.20 g·mol^{-1}
Sublimed Temperature	170°C
Sublimed Amount	133.5 mg for **pNiDPP**<br>111.5 mg for **pNiDNapP**<br>123.9 mg for **pNiDTP**<br>128.1 mg for **pNiDOMePP**<br>133.6 mg for **pNiDMP**<br>134.6 mg for **pNiDCOOMePP**<br>125.2 mg for **pNiDCNPP**

Table S2. Thickness of oCVD **pNiD(Aryl)P** thin films.

**Porphyrin**	Thickness (nm)
**pNiDPP**	51
**pNiNapP**	19
**pNiDTP**	40
**pNiDOMePP**	39
**pNiDMP**	100
**pNiDCOOMePP**	39
**pNiDCNPP**	45

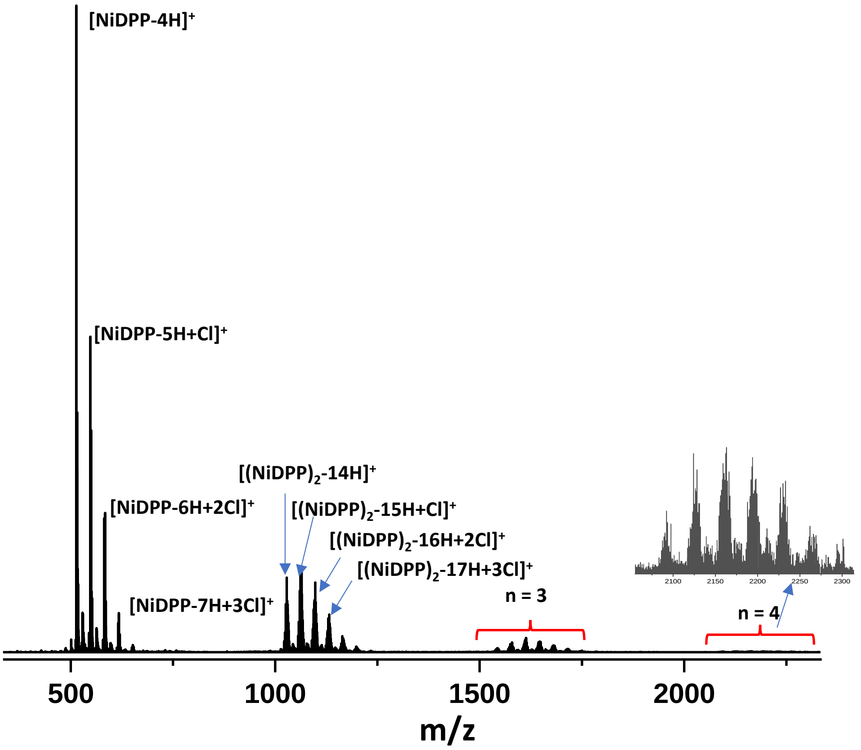

Figure S4. LDI-HRMS spectrum of **pNiDPP** showing the presence of different oligomers along with potential their potential chlorination.

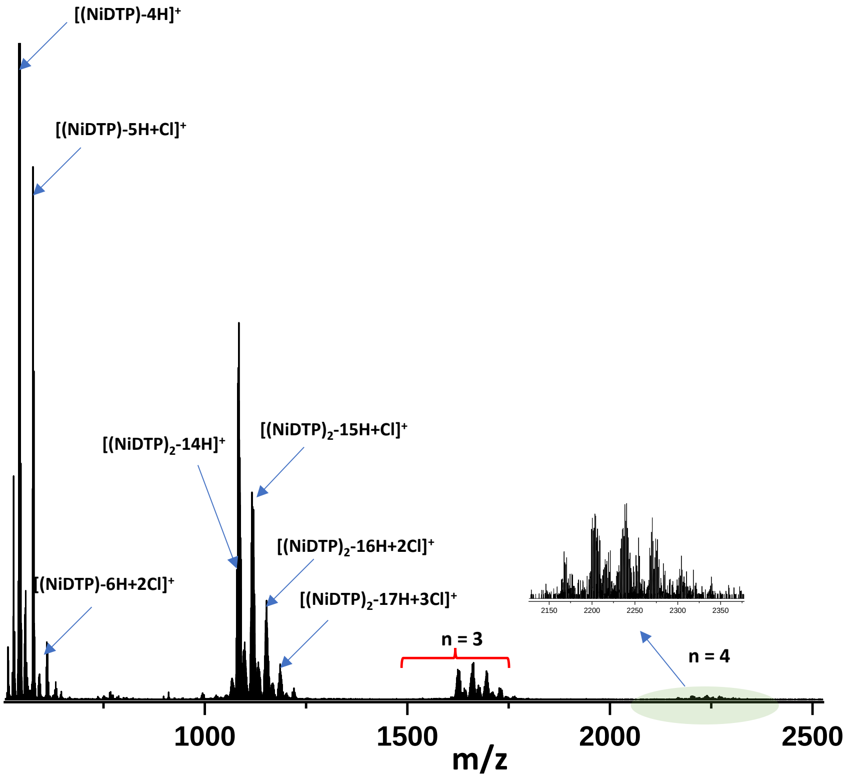

Figure S5. LDI-HRMS spectrum of **pNiDTP** showing the presence of different oligomers along with potential their potential chlorination.

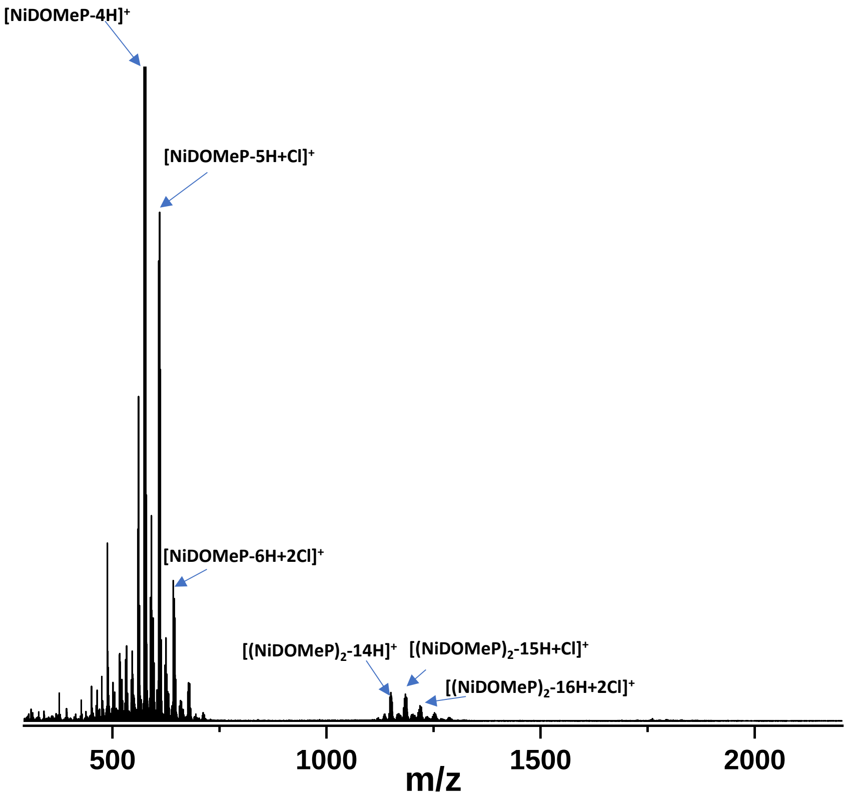

Figure S6. LDI-HRMS spectrum **pNiDOMePP** showing the presence of different oligomers along with potential their potential chlorination.

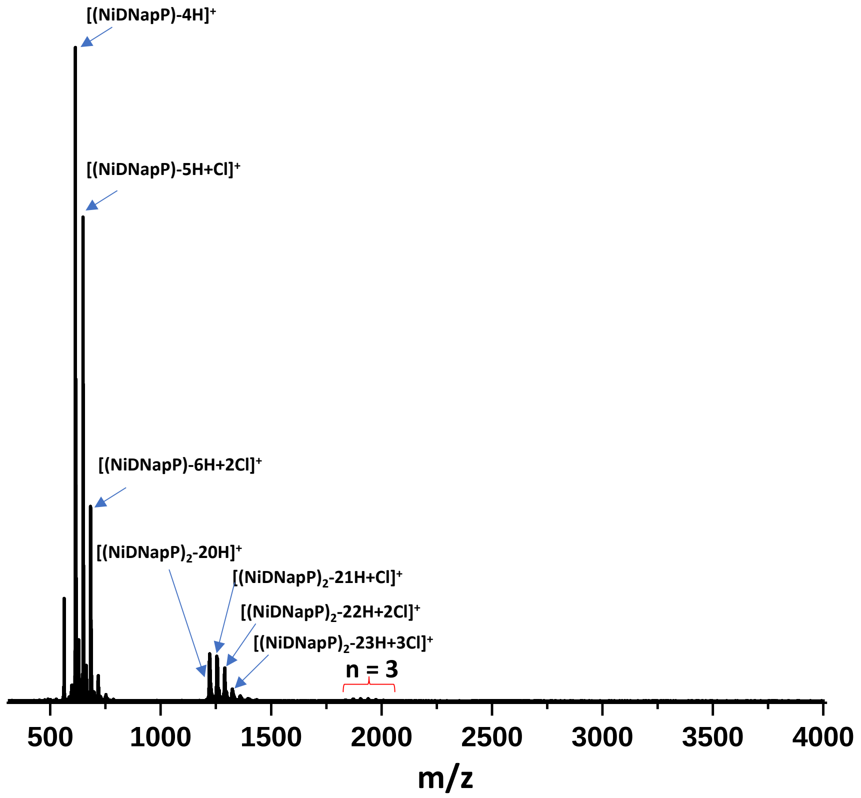

Figure S7. LDI-HRMS spectrum of **pNiDNapP** showing the presence of different oligomers along with potential their potential chlorination.

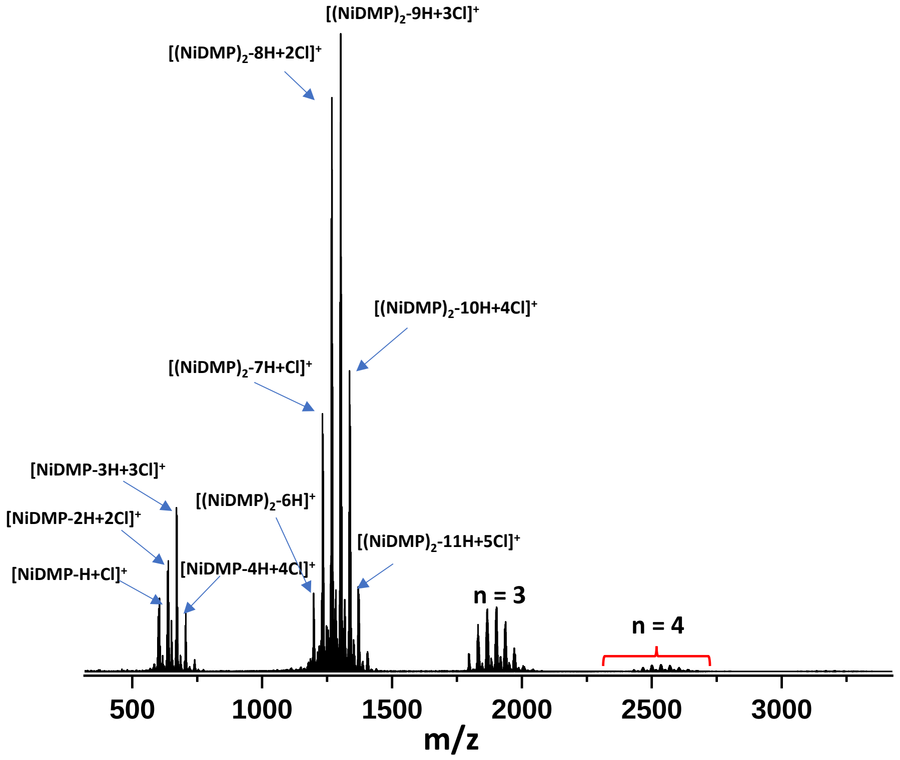

Figure S8. LDI-HRMS spectrum of **pNiDMP** showing the presence of different oligomers along with potential their potential chlorination.

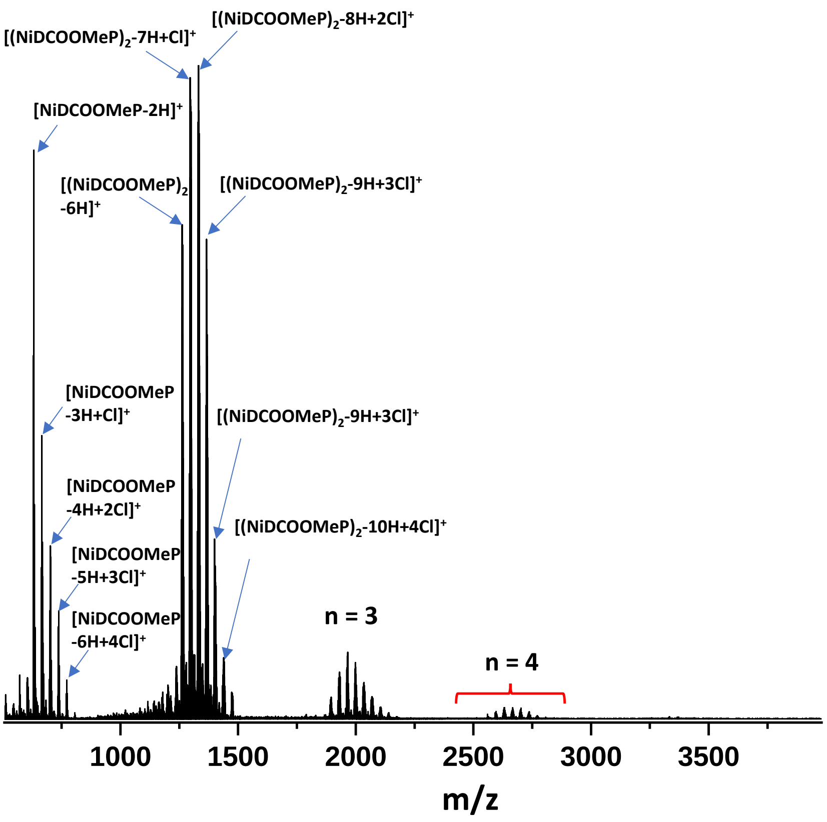

Figure S9. LDI-HRMS spectrum of **pNiDCOOMePP** showing the presence of different oligomers along with potential their potential chlorination.

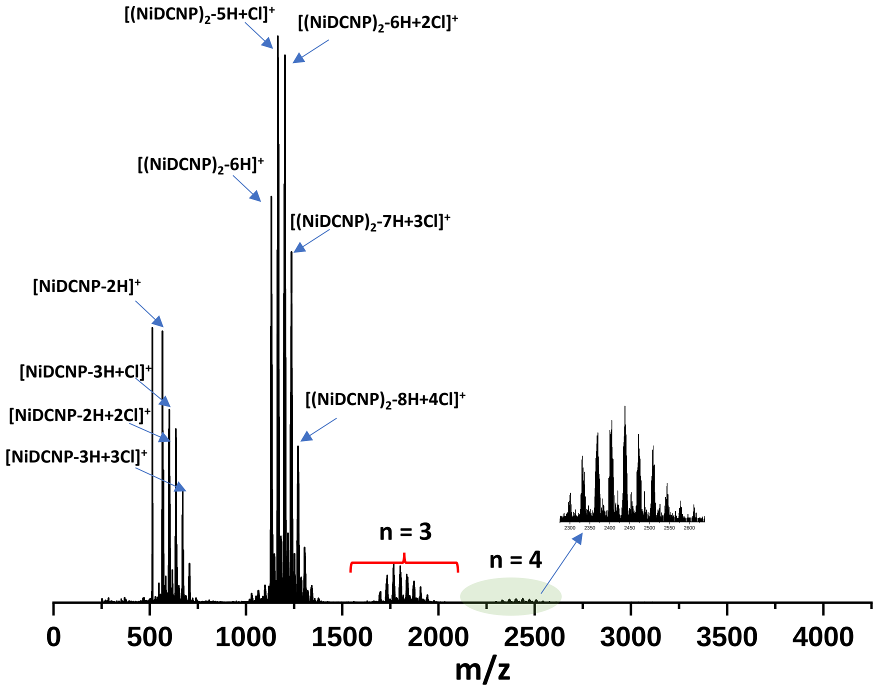

Figure S10. LDI-HRMS spectrum of **pNiDCNP** showing the presence of different oligomers along with potential their potential chlorination.

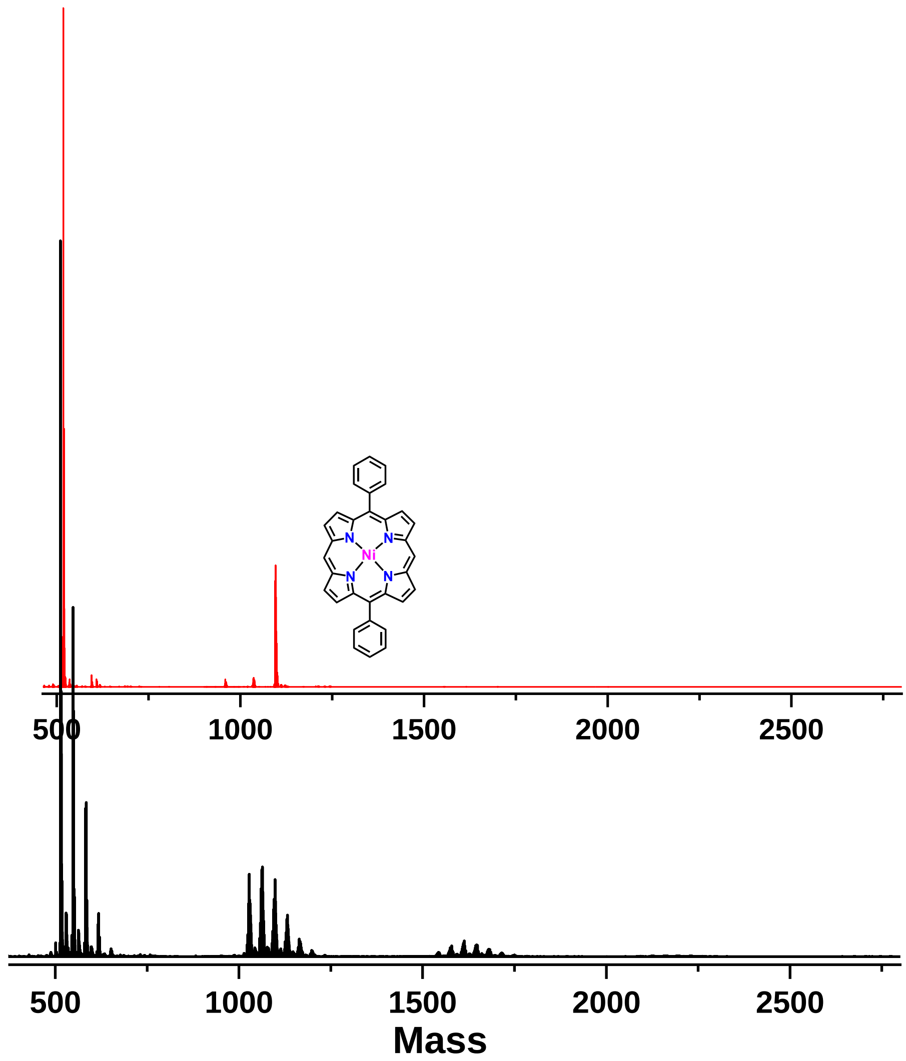

Figure S11. Comparative LDI-HRMS spectra of **pNiDPP** (black) with the reference sublimed porphyrins thin film (red) showing the absence of oligomeric features in sublimed materials in absence of $FeCl_3$.

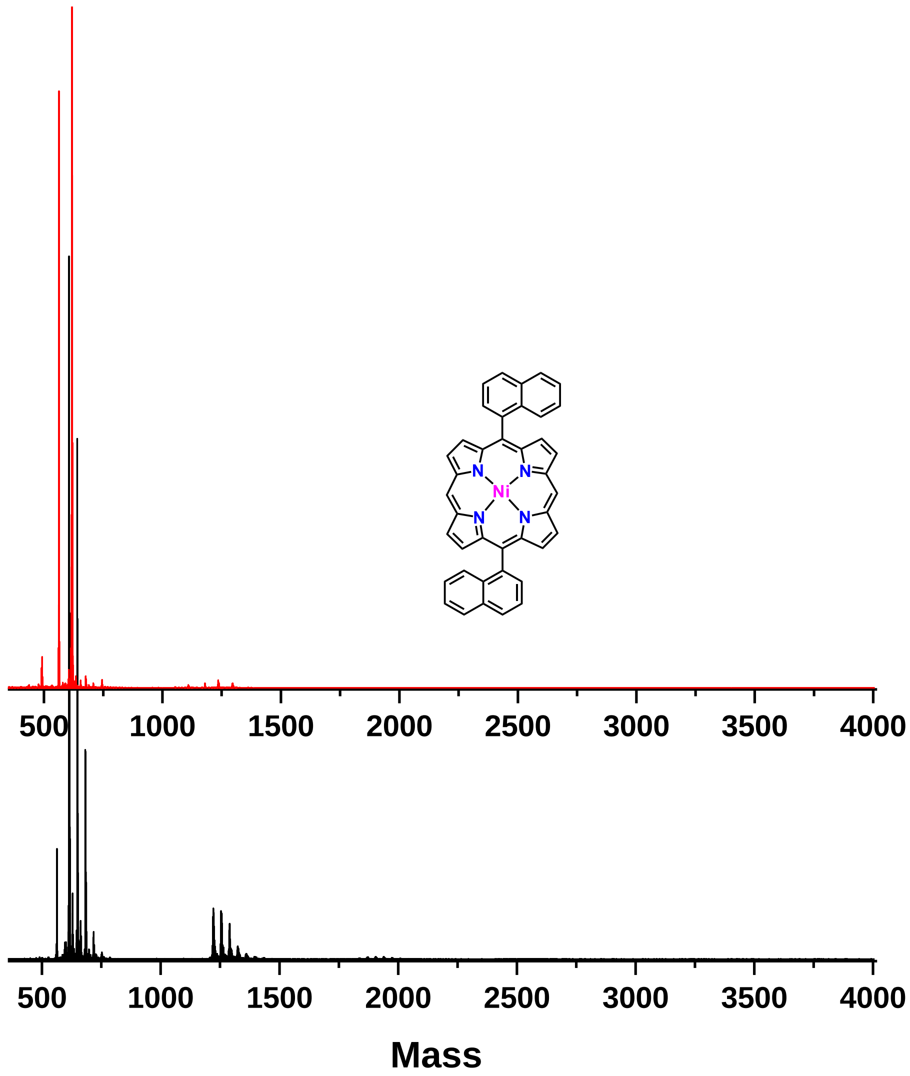

Figure S12. Comparative LDI-HRMS spectra of **pNiDNapP** (black) with the reference sublimed porphyrins thin film (red) showing the absence of oligomeric features in sublimed materials in absence of $FeCl_3$.

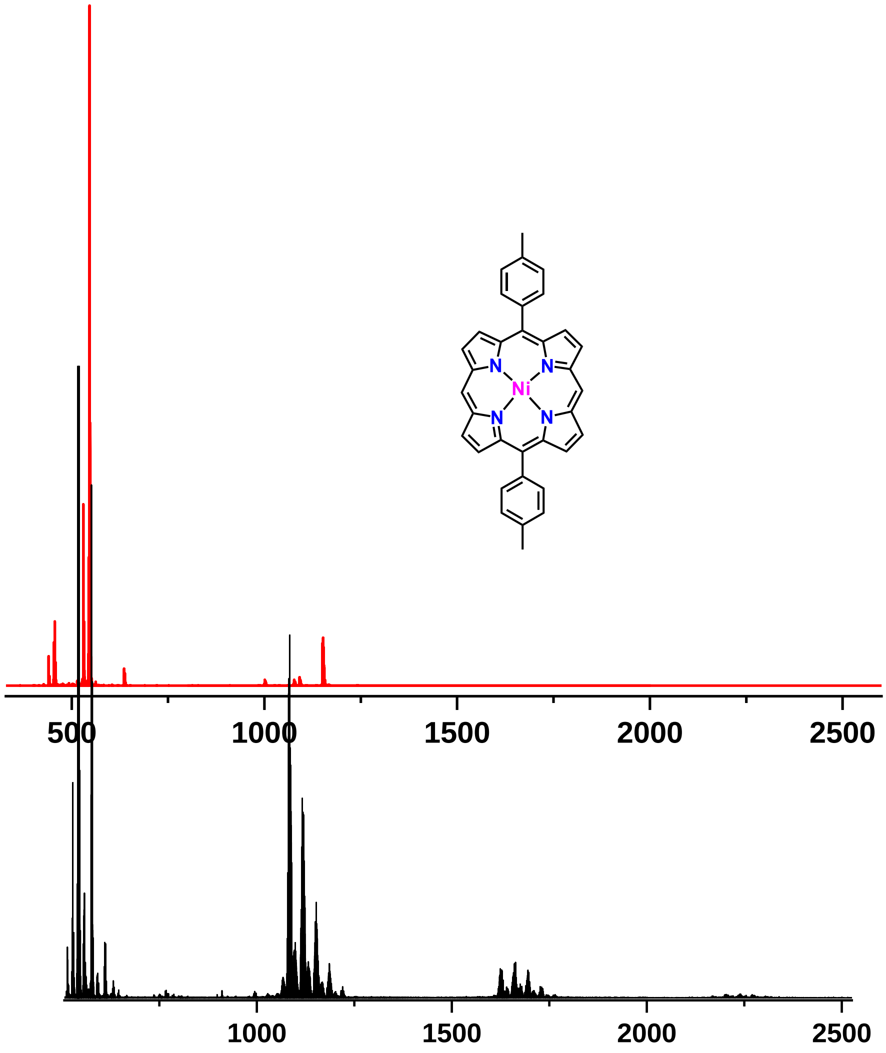

Figure S13. Comparative LDI-HRMS spectra of **pNiDTP** (black) with the reference sublimed porphyrins thin film (red) showing the absence of oligomeric features in sublimed materials in absence of $FeCl_3$.

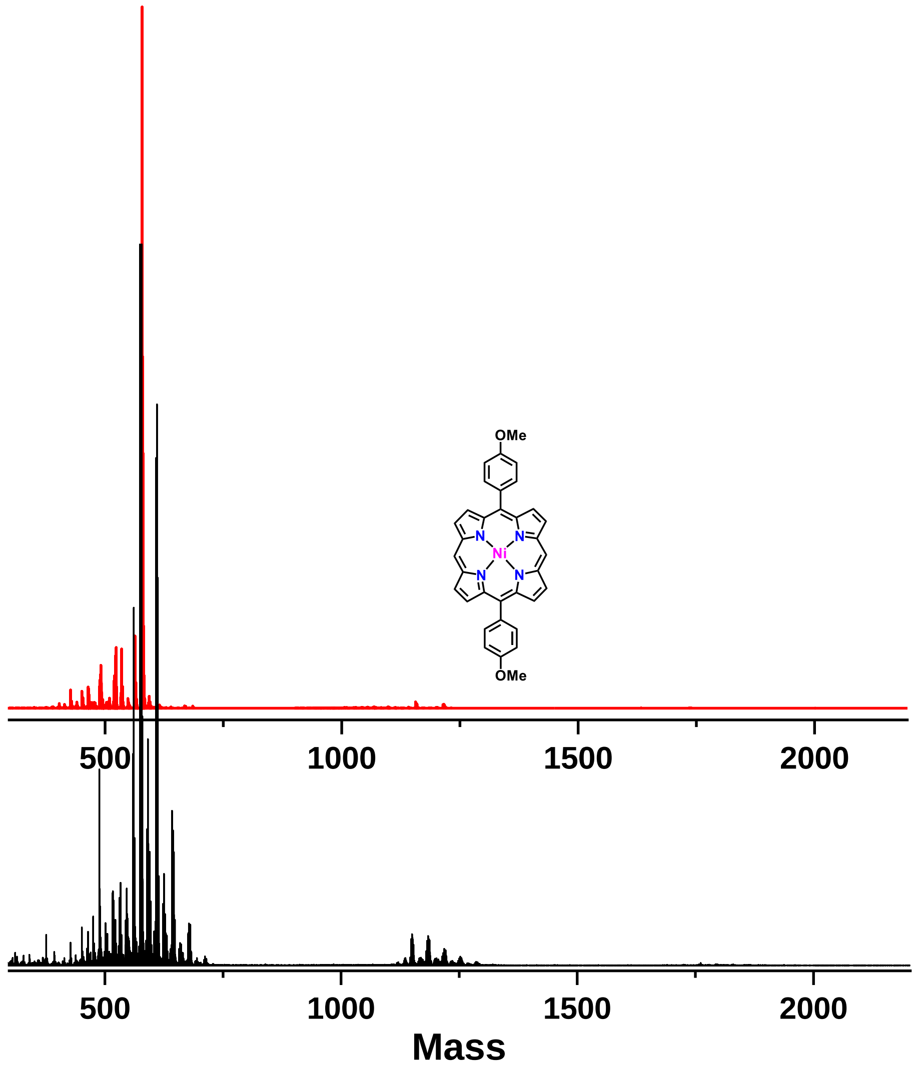

Figure S14. Comparative LDI-HRMS spectra of **pNiDOMePP** (black) with the reference sublimed porphyrins thin film (red) showing the absence of oligomeric features in sublimed materials in absence of $FeCl_3$.

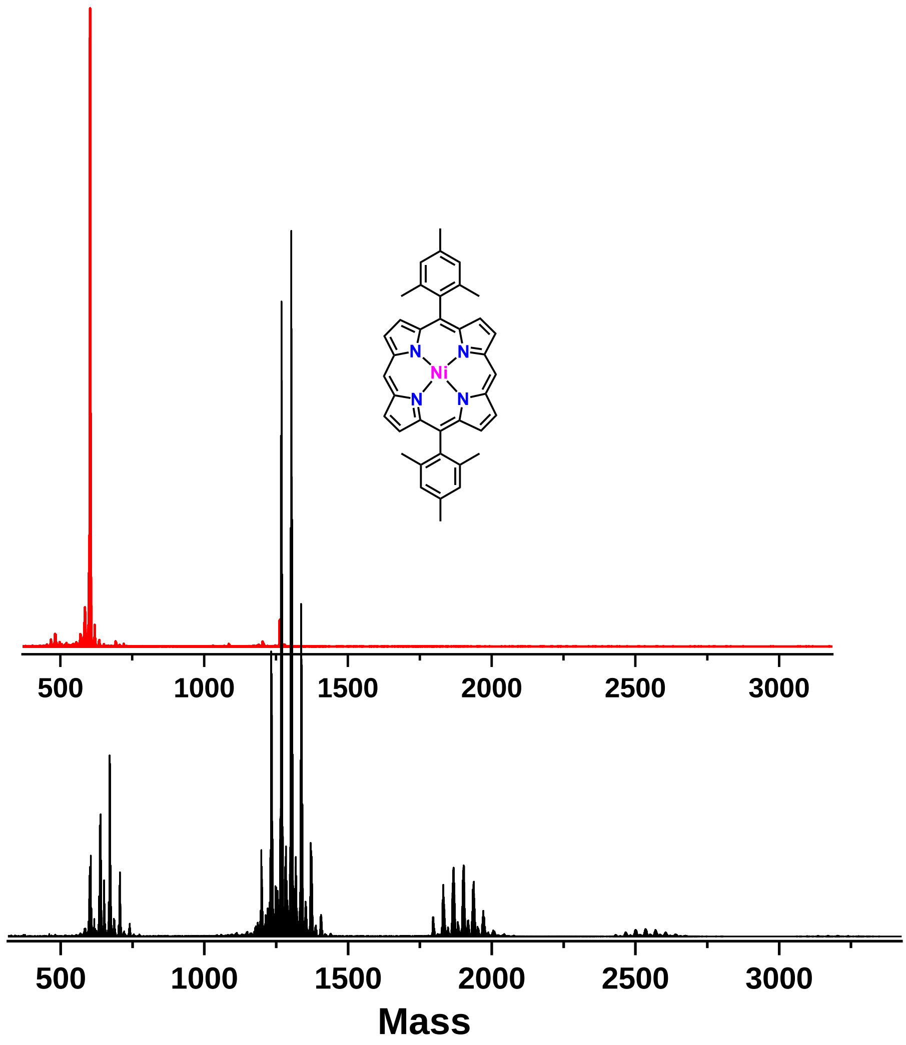

Figure S15. Comparative LDI-HRMS spectra **pNiDMP** (black) with reference sublimed porphyrins thin film (red) showing the absence of oligomeric features in sublimed materials in absence of $FeCl_3$.

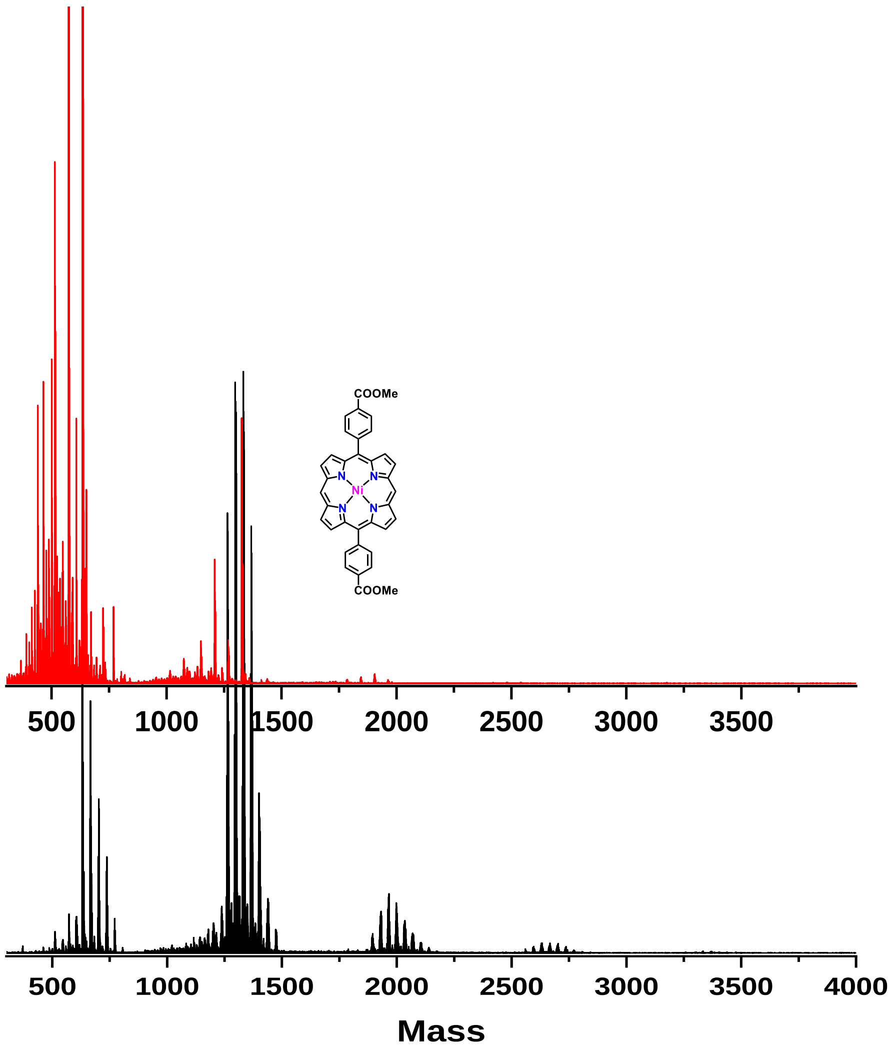

Figure S16. Comparative LDI-HRMS spectra **pNiDCOOMeP** (black) with the reference sublimed porphyrins thin film (red) showing the absence of oligomeric features in sublimed materials in absence of $FeCl_3$.

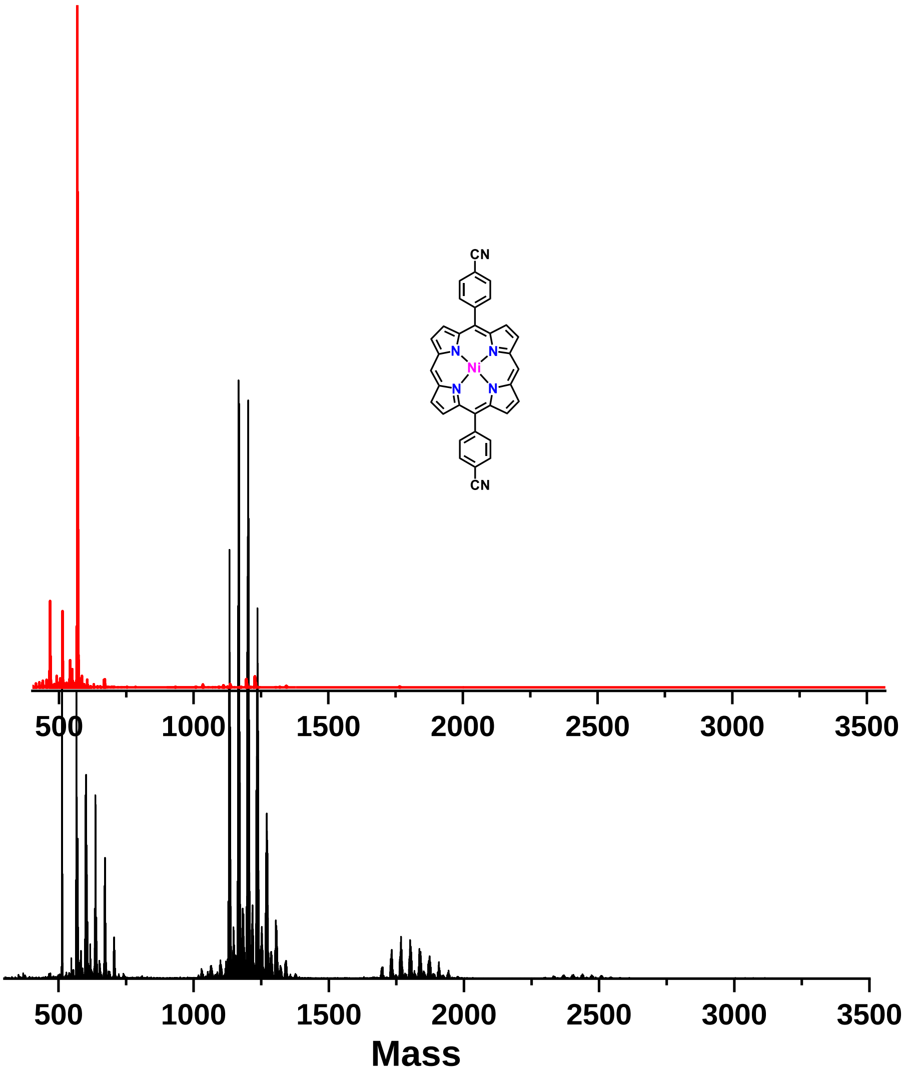

Figure S17. Comparative LDI-HRMS spectra **pNiDCNPP** (black) with the reference sublimed porphyrins thin film (red) showing the absence of oligomeric features in sublimed materials in absence of $FeCl_3$.

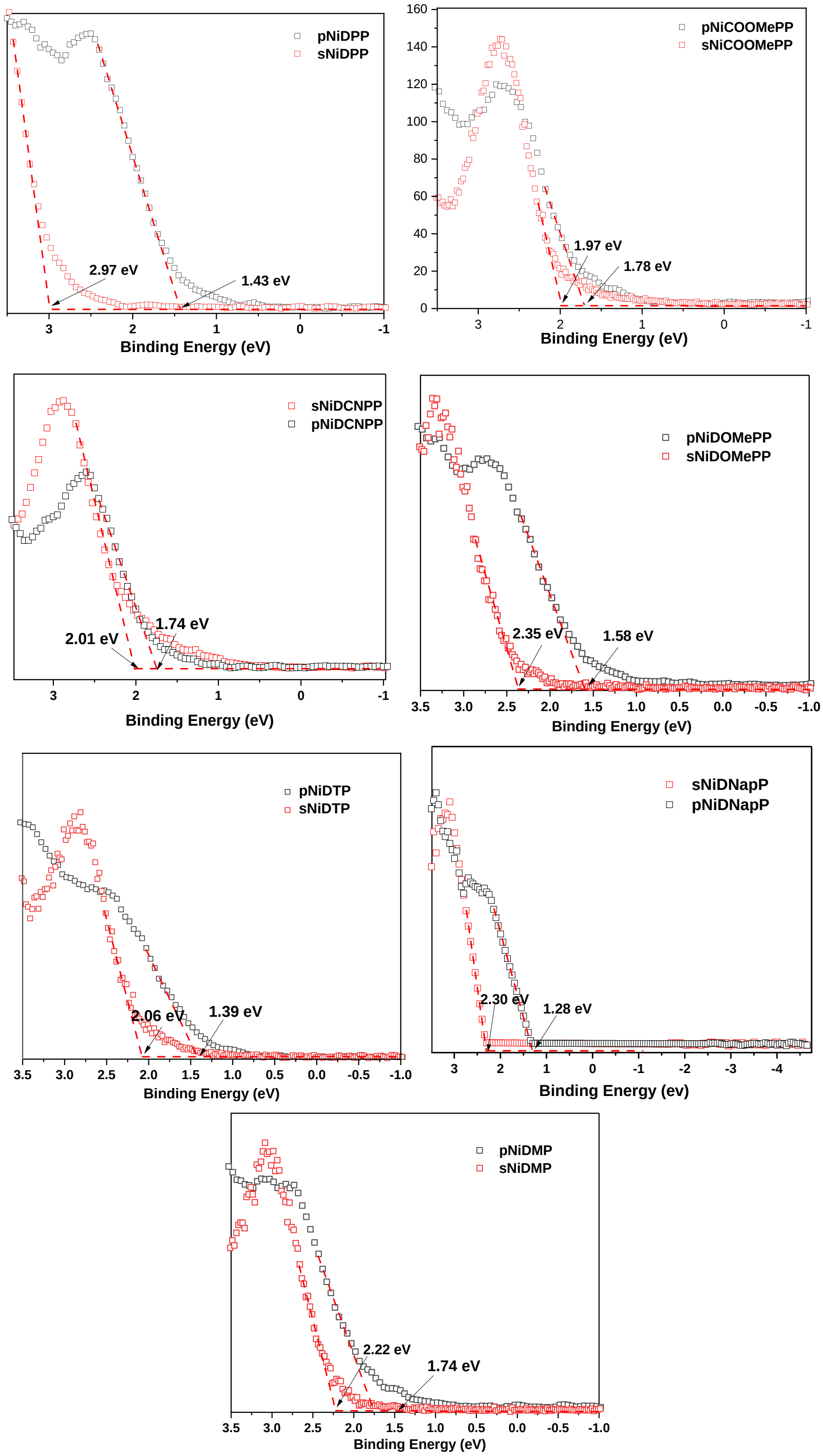

Figure S18. Valence band minimum energy (VBM) determination in the valence band region of the XPS spectra of the reference sublimed porphyrins thin films (red squares) and oCVD **pNiD(Aryl)P** thin films (black squares).

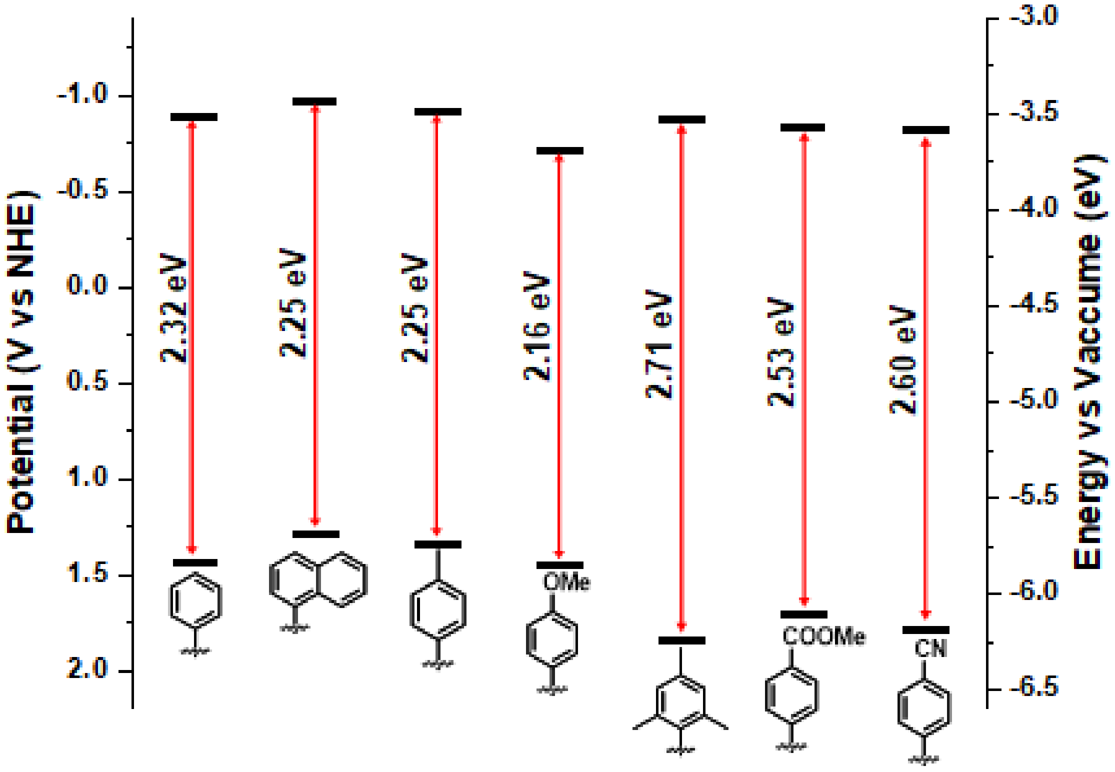

Figure S19. Band gap diagrams of the oCVD **pNiD(Aryl)P** thin films.

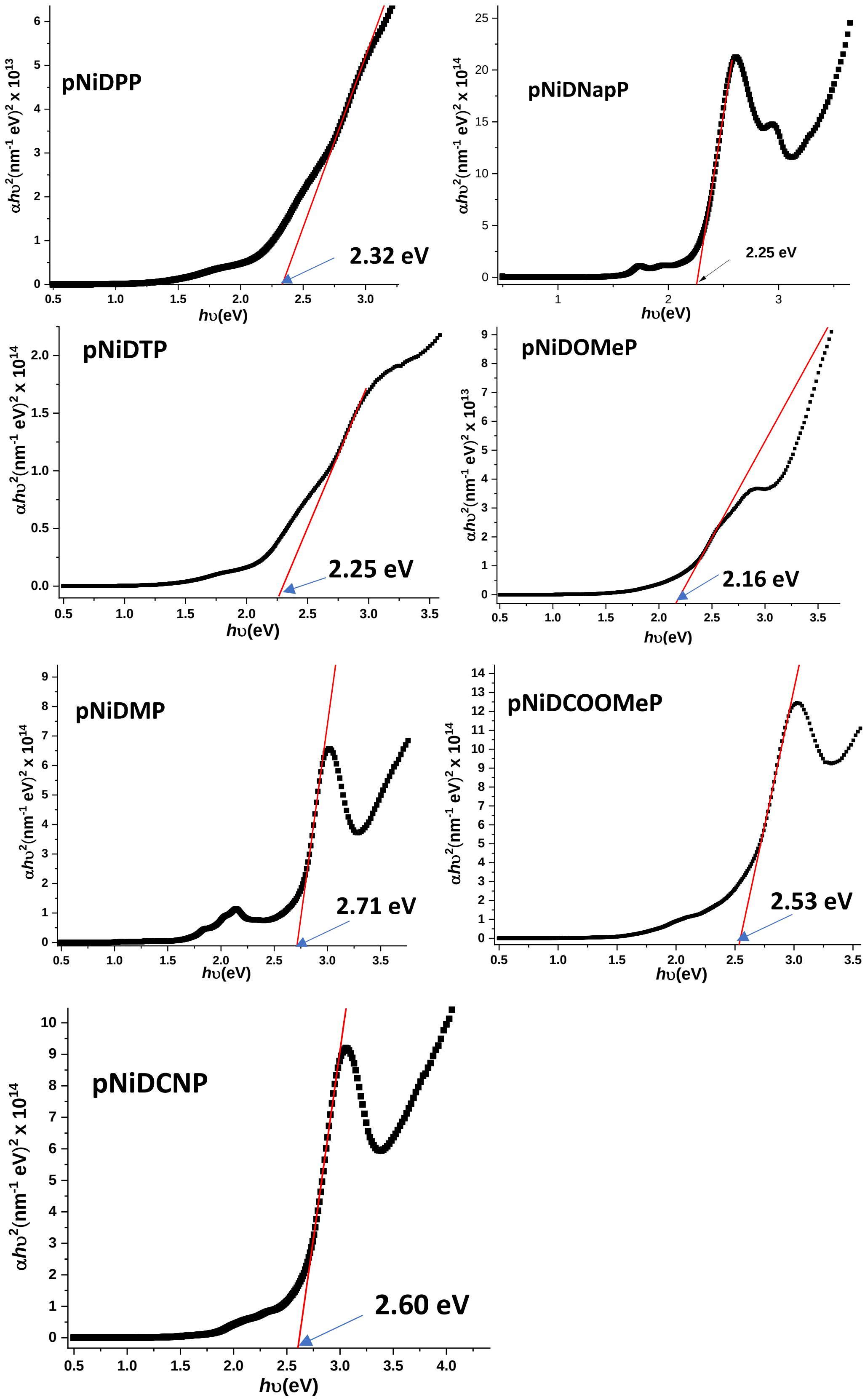

Figure S20. Energy band gap value estimation of the oCVD **pNiD(Aryl)P** thin films using Tauc's plot from the UV/Vis/NIR absorbance measurements.

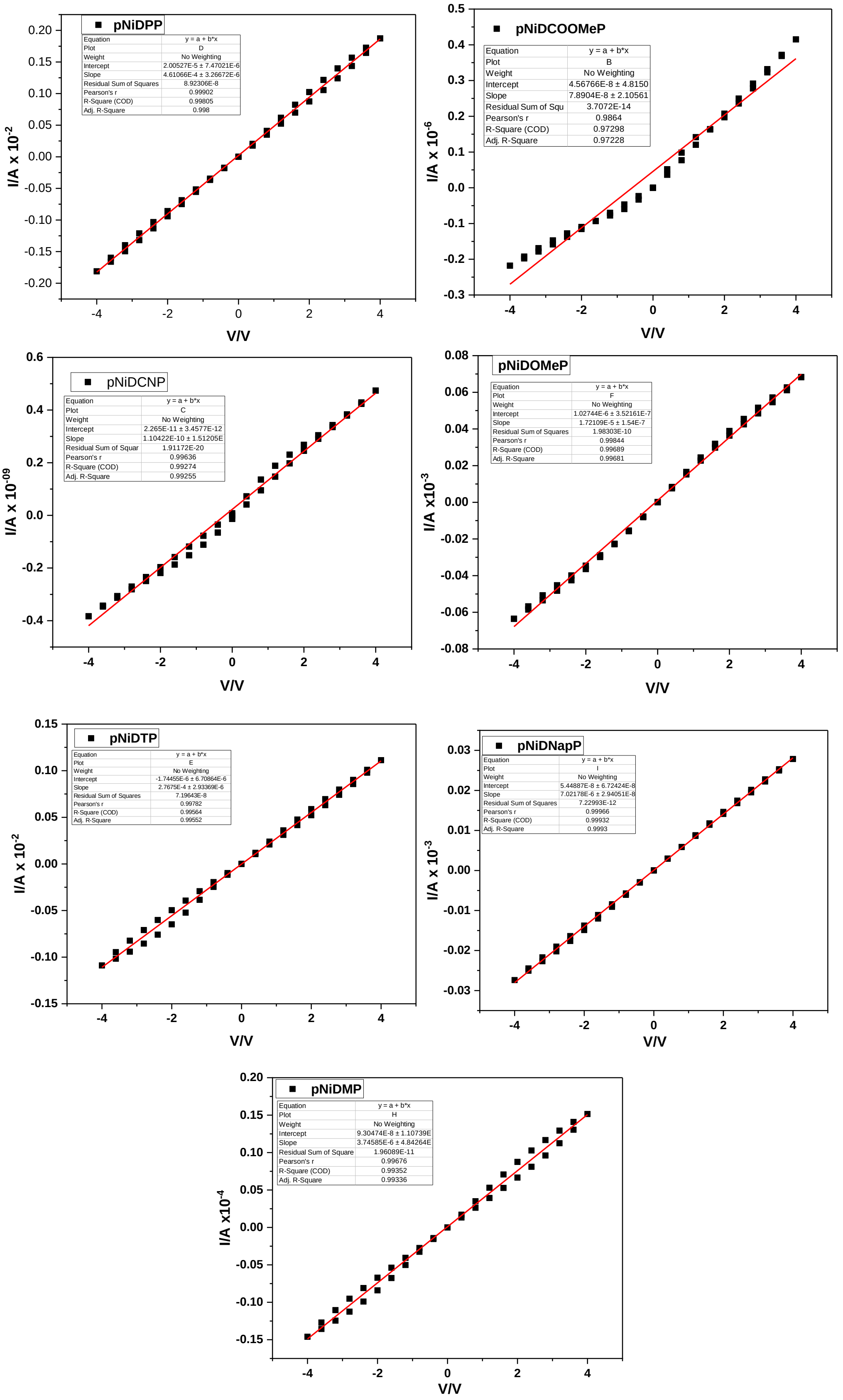

Figure S21. 2-point conductivity plot of as prepared oCVD **pNiD(Aryl)P** thin films.

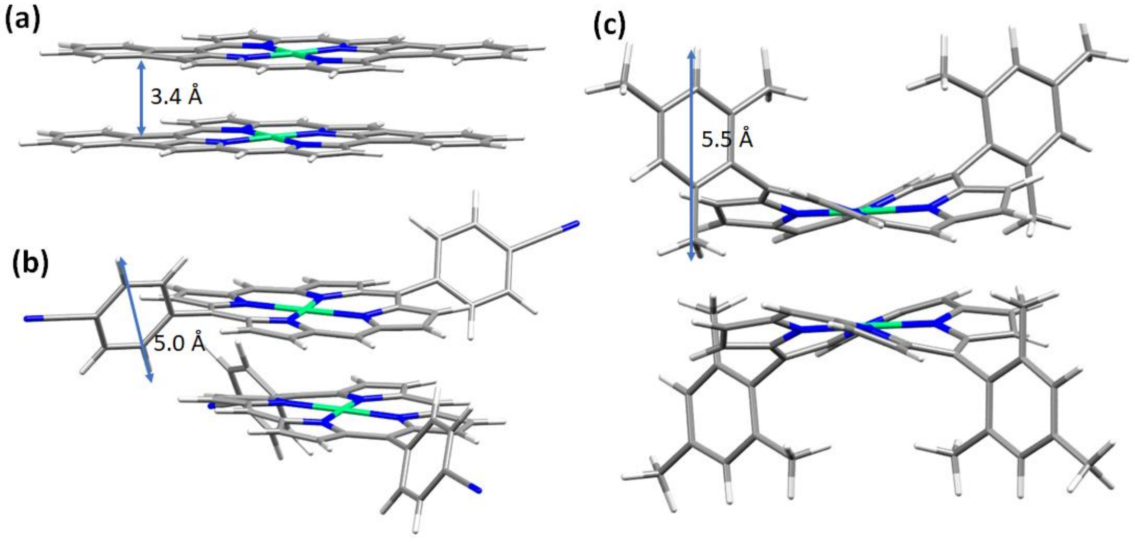

Figure S22. Representative optimized structure of $[NiDPP]_2$ (a), $[NiDCNPP]_2$ (b) and $[NiDMP]_2$ (c) dimeric aggregates showing the approximate spacing distance between the two monomer units. The structural planarity plays crucial role in conductivity of a polymer. Osuka and coworkers demonstrated that triply linked metalloporphyrin array presented highly stacked material with high electronic delocalization.[14,15] In 2020, we reported the effect of molecular flattening towards conductivity of fused porphyrin polymer.[16] It was shown that highly fused NiDPP aggregate ($[NiDPP\text{-}4H]_2$) exhibit highly planar and π-stacked arrangement. However, in case of NiDMP, with no possibility to form intramolecular dehydrogenation, aryl substituents are aligned out of the porphyrin plane resulting in the highly constrained π- π stacking with distance of $[NiDMP]_2$ aggregate to ~5.5 Å (assuming the presence of Mesityl/cyanophenyl group between the polymer chain(s)) as compared to 3.4 Å in $[NiDPP\text{-}4H]_2$ aggregate. Whereas, the presence of sterically lesser crowded cyano group in NiDCNPP afforded π- π stacked with $[NiDCNPP]_2$ aggregate distance of value between $[NiDMP]_2$ and $[NiDPP]_2$. Although, these results are mere approximations because these small models don't take into account that advancing polymerization and overlap between the growing chain and the substituents on the surrounding porphyrin chains. But it can be speculated that the increase structural planarity effect π- π stacking influencing the conductivity.

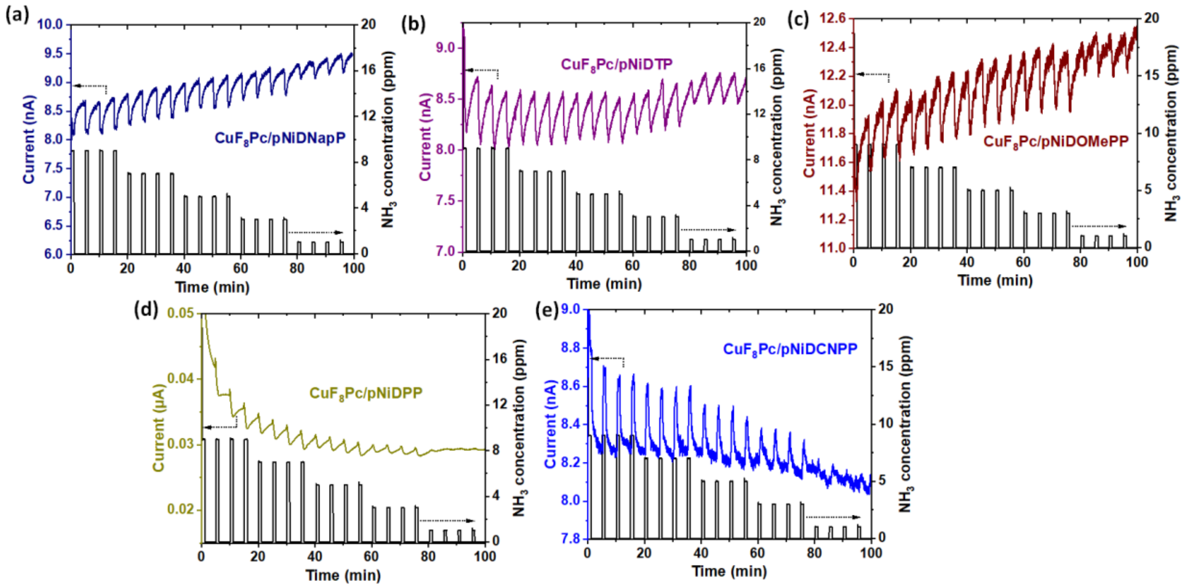

Figure S23. Response curves under successive NH_3 gas exposure (1 min) and recovery under clean air (4 min) in the range of NH_3 concentration from 9 to 1ppm at 45% of RH and room temperature (18–20 ˚C) for the **CuF_8Pc/pNiD(Aryl)P** bilayer heterojunction sensors prepared from *5,15*-diaryl Ni(II) porphyrins bearing different aryl substituents, (a) naphthyl, (b) tolyl, (c) 4-methoxyphenyl, (d) phenyl and (e) 4-cyanophenyl.

Table S3. Behaviour (n-type or p-type) of **CuF8Pc/pNiD(Aryl)P** bilayer heterojunction sensors upon NH_3 exposure.

Devices	Current (Under NH_3)	Polarity (Under NH_3)
CuF_8Pc/pNiDNapP	▼	p-type
CuF_8Pc/pNiDTP	▼	p-type
CuF_8Pc/pNiDOMePP	▼	p-type
CuF_8Pc/pNiDPP	▼	p-type
CuF_8Pc/pNiCOOMePP	▲	n-type
CuF_8Pc/pNiDCNPP	▲	n-type

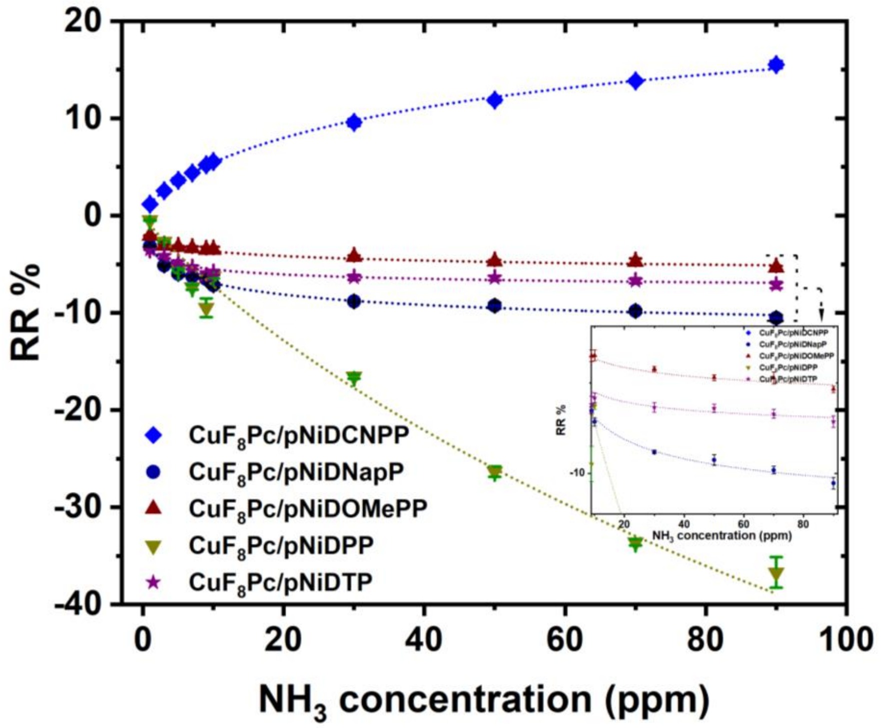

Figure S24. Relative response (calibration curve) of the **CuF8Pc/pNiD(Aryl)P** bilayer heterojunction sensors as a function of NH_3 concentration.

Table S4. Comparative analysis of previously reported bilayer heterojunction (BLH) devices with BLH devices reported in this work for NH_3 sensing.

**Device** (Type of charge carriers)	**$RR_{[90\ ppm]}$**	**Response time (t_{90}) (s)**	**S (% ppm^{-1})**	**LOD (ppb)**	**[NH_3] (ppm)**	**Reference**
**$Cl_2SiPc/LuPc_2$** (p)	-2.5	420	-0.13	100	1-9	[17]
**$(345F)_2SiPc/LuPc_2$** (p)	-10.5	60	-0.6	310	5	[17]
**$PTFANI/LuPc_2$** (p)[a]	-14	-	-1.05	450	1-10	[18]
**$CuF_{16}Pc/LuPc_2$** (n)	120	-	1.5	280	1-10	[19]
**CuPc/pNiDPP** (p)	-24.5	234	-	-	-	[20]
**$Cu(F_{16}Pc)/pNiDPP$** (n)	40.5	162	-0.4	228	1-9	[20]
**$CuF_8Pc/pNiDNapP$** (p)	-22.84	293	-0.98	190	3 - 1	This work
**$CuF_8Pc/pNiDTP$** **(p)**	- 26.02	43	-0.32	492	9 - 1	This work
**$CuF_8Pc/pNiDOMePP$** **(p)**	-11.46	64	-0.52	508	3 - 1	This work
**$CuF_8Pc/pNiDPP$** **(p)**	-56.22	198	-1.17	2000	9 - 3	This work
**$CuF_8Pc/pNiCOOMePP$** **(n)**	2.10	519	-	-	-	This work
**$CuF_8Pc/pNiDCNPP$** **(n)**	22.11	13	0.69	199	3 - 1	This work

[a] Double lateral heterojunction.

Table S5. Coordinates for triply fused NiDPP dimer with intramolecular cyclization.

	X	Y	Z
C	-2.06120832220257	-4.51326952335146	2.16063187832075
C	-1.06833809134202	-5.45994080747624	2.21338709082902
C	-0.08215385728185	-5.04243080633935	1.25559870127964
N	-0.40861698110820	-3.89600352625629	0.61688721847371
C	-1.64149201150642	-3.54116800411975	1.16180994443789
C	-1.85407156708855	0.28328382585220	-1.53857617455271
C	-2.59699102945073	-0.34925244182008	-0.58185483361216
C	-1.86892195876838	-1.52413028794115	-0.18853162830470
N	-0.68232743195074	-1.62185596498076	-0.89574926809724
C	-0.66934237898242	-0.50143370350036	-1.73484235631178
C	-2.33119326308154	-2.41298341603128	0.77515818544769
C	3.43745771629895	-1.68895594942991	-3.56687365446959
C	2.44218294682895	-0.72010686701942	-3.62845098532164
C	1.45192289987461	-1.12531590886692	-2.67134197125072
N	1.75884055553011	-2.27458910378150	-2.01875208898329
C	2.97454913490950	-2.63749819947959	-2.55317415984372
C	0.38002468055602	-0.24928821077980	-2.61246198760969
C	3.22827999874425	-6.45597450729727	0.12840882422901
C	3.97924052350086	-5.81983984580647	-0.84267750303637
C	3.22317756126044	-4.64249947996650	-1.22022693610475
N	2.04931317753655	-4.54808140684737	-0.51509889868391
C	2.04825233236764	-5.67578885945522	0.32628693603752
C	0.99607669982562	-5.91894590285737	1.20062288954864
C	3.69848880178413	-3.75898398371146	-2.19462755386926
Ni	0.67600532512240	-3.08418365133059	-0.70192929751767
C	0.68393792613053	0.81712780166488	-3.60323714582451
C	0.70017051651939	-6.98550605820970	2.18951471966846
C	1.95362191295685	0.51839432231775	-4.22212245597326
C	2.47830884512429	1.36357258004624	-5.19262996003879
C	1.75883694264383	2.51217703020577	-5.56531475203687
C	0.53276877067059	2.80261139420708	-4.96951789054375
C	-0.01173221227573	1.95652182252851	-3.98455301322040
C	-0.56792263717698	-6.69956403077222	2.80855299084604

C	-1.08640884222256	-7.54621501352075	3.77933084416765
C	-0.35620945800487	-8.68812644498929	4.15147229803429
C	0.87264894306220	-8.96880676675706	3.55492768054832
C	1.40837988093012	-8.11975674676464	2.57066047392660
H	-2.99124552129192	-4.44293167933422	2.71861244378200
H	-2.08863911650865	1.20144094409747	-2.06553620355466
H	-3.55802029352097	-0.05698368669557	-0.16762837659974
H	-3.29039964201575	-2.19816906376260	1.24517655083514
H	3.46664160908136	-7.37425155161245	0.65263732588173
H	3.43784227761032	1.14022873873865	-5.65973108136210
H	-0.01518031195724	3.69709853544239	-5.26765696605404
H	-0.97106262153918	2.20849892253950	-3.53461618118020
H	-2.04744021273756	-7.32893590130740	4.24739576273848
H	1.42812011839225	-9.85839764102890	3.85392235408892
H	2.37029453394802	-8.36245347949218	2.12130237476886
C	5.23539864241847	-6.08172009956227	-1.47988807216465
C	6.23107030448216	-7.05014529643030	-1.41786052649010
C	7.22191056469440	-6.64412961174201	-2.37391871109820
N	6.91505757216681	-5.49481605069757	-3.02640388310958
C	5.69862897909225	-5.13275274149220	-2.49314298358405
C	5.44287038566236	-1.31643291661596	-5.17774533308220
C	4.69332172407817	-1.95112785684340	-4.20462007026544
C	5.45006056875482	-3.12775239755360	-3.82619350833835
N	6.62303104776073	-3.22307223403724	-4.53267937358341
C	6.62276533228807	-2.09676269992644	-5.37596236376211
C	4.97499167527388	-4.01097834308927	-2.85139156402698
C	10.72972839410489	-3.26205616943309	-7.21431228270501
C	9.73581029321530	-2.31653696695521	-7.26836079072500
C	8.75150724710008	-2.73197580288587	-6.30784316703098
N	9.07991595309151	-3.87626019445283	-5.66639694363094
C	10.31243171857784	-4.23152681351195	-6.21187862586751
C	7.67363200234502	-1.85501842234128	-6.25227774593829
C	10.53477316930064	-8.04481375732124	-3.49642100762731
C	11.27632157967322	-7.41380175203535	-4.45519324555416
C	10.54498875448972	-6.24261885452262	-4.85348963332339

N	9.35769075467539	-6.14571761599107	-4.14732554574003
C	9.34726438384084	-7.26325206325659	-3.30435363647147
C	8.29684261116806	-7.51668032165032	-2.42833116071512
C	11.00465160321054	-5.35676581043270	-5.82118674820235
Ni	7.99718533628055	-4.68618402862866	-4.34466310373724
C	7.96812458185610	-0.79008466126862	-7.24334342344203
C	7.99423382075628	-8.58193024559572	-1.43598960372216
C	9.23477898423842	-1.07781098156256	-7.86474602019518
C	9.75191509817328	-0.23235322179114	-8.83729017181592
C	9.02211180820313	0.91008542257870	-9.20866871955003
C	7.79501178988620	1.19272490766737	-8.60945465069722
C	7.26051800747901	0.34489253098006	-7.62337819986528
C	6.72229703984258	-8.28579882976104	-0.82033214972413
C	6.19797330869909	-9.13022095895296	0.15088941539545
C	6.92035308243979	-10.27573860591105	0.52787965016329
C	8.14848281134114	-10.56363639982325	-0.06457485165500
C	8.69254809161606	-9.71825660287635	-1.05037891763910
H	5.20250705366086	-0.40012347556742	-5.70450036890818
H	11.65882120547253	-3.33341888641772	-7.77371961665734
H	10.77158079175397	-8.96031182428536	-2.96585116757336
H	12.23846300917226	-7.70474971832480	-4.86776824357801
H	11.96410634198361	-5.57136466218668	-6.29080498242507
H	10.71164056532663	-0.45109033068457	-9.30737639415046
H	7.24003648333399	2.08289469339314	-8.90764595360249
H	5.23683168111078	-8.90877392207479	0.61556863253323
H	8.69868421296473	-11.45563024070688	0.23690282715541
H	9.65376591643227	-9.96808986839694	-1.49748014381136
H	9.41987805933391	1.58016509423523	-9.97158611234952
H	2.16428647552182	3.17994281495786	-6.32611747055479
H	6.51534561530501	-10.94298518429929	1.28936880895805
H	-0.75514811175583	-9.35903841169484	4.91303692986476
H	6.30021480891051	0.58956289483234	-7.17168323931190

Table S6. Coordinates for doubly fused NiDPP dimer with intramolecular cyclization.

	X	Y	Z
C	-3.32696033410742	-3.88178270288835	-0.80885086886319
C	-2.47020946926771	-4.89420416400502	-0.47726203695656
C	-1.16078226394359	-4.49266693523108	-0.90697188570390
N	-1.21531210072590	-3.23070104236750	-1.50449671567295
C	-2.54290008986979	-2.84844393909552	-1.42831181285188
C	-1.59014842704573	1.47106325445472	-2.93399570590261
C	-2.71957698637498	0.78420783713571	-2.56000534254896
C	-2.29218290695927	-0.58193503934789	-2.28656079733762
N	-0.92485642506968	-0.72018150043295	-2.51825819823130
C	-0.52454713458247	0.50823584882652	-2.90472563941433
C	-3.06793646418028	-1.61570381454449	-1.80824507589306
C	3.78260183406001	-0.59577252882029	-4.16729396764450
C	3.06877594118800	0.56571590253225	-4.05773011402561
C	1.78855753194489	0.22142504350733	-3.51161813523625
N	1.73555776892669	-1.15315737879617	-3.24172846189215
C	2.94851667619396	-1.65518119078755	-3.67238620269794
C	0.68510294890734	1.04502588975190	-3.33445521642130
C	2.26781082433127	-5.66512407401439	-1.35997166173876
C	3.28978206139925	-5.08335785383754	-2.11374315336778
C	2.72769755895560	-3.82321046407980	-2.61199045272069
N	1.46598777347890	-3.60195843058262	-2.11156206767841
C	1.19001431979252	-4.71935046614384	-1.39158936944309
C	0.02659109276713	-5.21255653156971	-0.81644851892943
C	3.39175819464238	-2.98312037074616	-3.50688757146439
Ni	0.25596390299430	-2.18331349418816	-2.35429839680755
C	0.40728371879352	2.47764750010103	-3.61440145205576
C	0.36910230492727	-6.57332450117306	-0.33294839660171
C	1.22962767717635	3.51594647369225	-4.03722357405894
C	0.68121981960181	4.79704612304957	-4.22446936633921
C	-0.67295770462675	5.03805577018084	-3.99339849275239
C	-1.51914589623745	4.00169646041442	-3.56350232532304
C	-0.98970755312315	2.73210873182216	-3.37246825625499
C	-0.39192746163376	-7.53731076880106	0.31533649623130

C	0.17144732292580	-8.79740452094138	0.58687435224850
C	1.47669103674078	-9.08985349972406	0.19775274673174
C	2.26816693806097	-8.12160779197929	-0.44372870501077
C	1.73717765139239	-6.85925603718572	-0.69117687355768
H	-4.39850537795387	-3.82088331222179	-0.63931054505024
H	-2.70721661395340	-5.83394328424055	0.00828942894572
H	-3.74504217429543	1.12958618247587	-2.45781485542786
H	-4.13543635903783	-1.45122190520502	-1.66538962555858
H	4.80578901295995	-0.72358727958714	-4.50174618763492
H	3.38986517151861	1.56733871189128	-4.32056480946008
H	2.29126920290987	3.35574287943329	-4.22008187663151
H	1.32689965092777	5.61148238392440	-4.55488070735551
H	-2.57759388427609	4.19267721785034	-3.38164424174892
H	-1.41979731715970	-7.33540743012617	0.61309312476335
H	-0.42765308023249	-9.55397072452291	1.09480553141103
H	3.28615440483302	-8.36225976493260	-0.74629199895825
C	5.18898034126087	-6.79384556968273	-0.44776453507475
C	6.23815085814421	-7.58425306308462	-0.06666646329600
C	7.12529001579900	-7.67386676473572	-1.18946273949706
N	6.63905446261105	-6.89561125582913	-2.24900001453657
C	5.43709089526548	-6.38159809756305	-1.80111606069319
C	5.32776921506507	-3.24527789532887	-5.27166353200942
C	4.56144550770445	-3.54419329955351	-4.14262304755976
C	5.17004588152787	-4.75292934363413	-3.57591126936497
N	6.30209329715128	-5.11736886007525	-4.26634333840884
C	6.36969502222977	-4.23047569889821	-5.29185706153606
C	4.63499962239365	-5.45039036128530	-2.49202187845163
C	9.62808227116682	-6.45825970236590	-7.71335341784311
C	8.87527715717393	-5.33145819589627	-7.89431983377446
C	8.08283654303419	-5.16219813325145	-6.70946824257687
N	8.34996251371749	-6.18873946191422	-5.80010457002389
C	9.31216835219197	-6.97440250021205	-6.40948344324882
C	7.13718107941107	-4.17559073636591	-6.44749496039891
C	9.97762698882846	-9.50600516348622	-2.53864923667323
C	10.49135286359203	-9.49951410446140	-3.81280232269576

C	9.75120924988421	-8.47901589208349	-4.54396157722164
N	8.78614732746959	-7.89629977216520	-3.72457864658041
C	8.93296549612933	-8.52003975749474	-2.53803141839984
C	8.26036210001713	-8.46249239219720	-1.32128621168255
C	9.96663776621300	-8.06417325857880	-5.84039243125728
Ni	7.51428788707835	-6.53353710738974	-4.02071243674671
C	6.58846838982929	-3.04333882441228	-7.23464549170815
C	8.94829517138539	-9.44189560624431	-0.44057033115752
C	6.95095808306895	-2.52307159734187	-8.47016773611570
C	6.19161303225223	-1.48092681920745	-9.03241943878463
C	5.07385723853475	-0.98016030819751	-8.36859193716051
C	4.70164534837448	-1.48212428528508	-7.10958977120455
C	5.46095144398693	-2.49080348406056	-6.52402539837708
C	8.74503985671374	-9.79530564819455	0.88860236212304
C	9.56466509488972	-10.77616365461617	1.47430110354350
C	10.57506817661148	-11.39749630167700	0.74060857480103
C	10.79744836057637	-11.05126401890302	-0.60311991956559
C	9.99619618569973	-10.08250445250799	-1.19315264603690
H	4.34455650082498	-6.46883189466262	0.14930523673662
H	6.39456752138683	-8.05778467954130	0.89601654672949
H	10.35123550427350	-6.90368451832485	-8.39106399350621
H	8.85181432566034	-4.67874887853711	-8.75939386088345
H	11.28904385388372	-10.09630999823877	-4.24732834310235
H	10.72278502395964	-8.57226764925789	-6.43796433092108
H	7.81108169426357	-2.91279919123521	-9.01244209763536
H	6.47614771035818	-1.07349174459360	-10.00315314762185
H	3.82356720941054	-1.08584736502634	-6.60197082175874
H	7.96712287619400	-9.32132677310603	1.48554419572447
H	9.40547372461559	-11.05231015564002	2.51726335473443
H	11.58987775819342	-11.53672003120220	-1.17432131406194
H	-1.07904295813098	6.03864706889858	-4.14574845776397
H	4.47895531058003	-0.18886083388994	-8.82613498559676
H	1.89347690935075	-10.07869973146306	0.39229983194858
H	11.19918162049795	-12.15697170492137	1.21282948506672

Table S7. Coordinates for triply fused NiDNapP dimer.

	X	Y	Z
C	-4.19654781464180	-4.77306104497387	-0.40449895532373
C	-3.13384771884557	-5.46185212097619	0.16511590671033
C	-1.92156723570116	-4.86367194547039	-0.30461006253820
N	-2.21427133223254	-3.82070060612769	-1.17140070020918
C	-3.58504454535019	-3.71983797272239	-1.19048219738162
C	-3.42301492777864	0.57678755497962	-3.04760420163668
C	-4.40415667856996	-0.31546788206990	-2.57266303826283
C	-3.70225643858799	-1.54300977674159	-2.28549249871729
N	-2.35829039316541	-1.43519879264915	-2.55894247954395
C	-2.17345782747415	-0.14878517613538	-3.00552437337449
C	-4.32513373504437	-2.65761067267029	-1.71351228429115
C	1.84710382161855	-1.29999947514353	-4.97650913947036
C	1.10968386246357	-0.15948357946457	-4.81037227935133
C	0.08866793402255	-0.45030747531919	-3.83344336169065
N	0.20392602831667	-1.75999801621694	-3.41937800636297
C	1.30920331939696	-2.28272501074266	-4.07899661978031
C	-0.97810616617364	0.40384299485517	-3.47377140657517
C	1.57357428490041	-5.87055802220419	-1.10203513113335
C	2.23715013057218	-5.43859327724430	-2.23685613417648
C	1.49265243268776	-4.33648866496980	-2.76211159754232
N	0.34840830618538	-4.12326581589319	-2.00286685962911
C	0.38903837097036	-5.05107079630551	-0.99696587754956
C	-0.61470042567650	-5.33787720367769	-0.06528292740616
C	1.89645609988599	-3.50615624099018	-3.80030713432258
Ni	-0.99664364631422	-2.77170949883531	-2.26738234056848
C	-0.94998309751861	1.84758858980741	-3.68878587211599
C	-0.27232391924881	-6.24362932306503	1.03150851742408
C	0.24952463421483	2.56580003388174	-3.75237866016917
C	0.26721986721856	3.94219069535846	-4.03501744392068
C	-0.91270177658208	4.62141789417594	-4.26803584000235
C	-2.15946501535229	3.95382063296842	-4.15358384184743
C	-2.18398189856336	2.56637759658780	-3.79977764003400
C	-1.03462104542558	-6.30482528728951	2.20201151096797

C	-0.74318227040890	-7.22446430918949	3.22532382365503
C	0.32925763081268	-8.08554037158819	3.10694706515252
C	1.18868714751358	-8.00827690703222	1.97990040878615
C	0.90848453210677	-7.05707396352159	0.94615770486480
H	-3.16320185094223	-6.31782613042278	0.82304438944509
H	2.68912122252522	-1.46539720300486	-5.64285055989478
H	1.20176443736358	0.77404650661187	-5.35327133333519
H	1.18820086014534	2.05060987654206	-3.55535948746895
H	1.22181210675133	4.46816964767689	-4.07628772719017
H	-1.85868897244032	-5.60649915215825	2.33716482283676
H	-1.37094463042281	-7.24684617172982	4.11694658380791
H	-0.90609669652261	5.68265749283647	-4.52309977552643
H	0.55180679121584	-8.81265745476919	3.89002648265625
H	3.16321927157878	-5.81190161226730	-2.66489621438401
H	2.79396270072034	-3.77825407689446	-4.35541218027890
C	2.33128331259860	-8.84132383781041	1.86545103224692
C	3.18618321346248	-8.72074518669161	0.78546128904116
C	2.94393553977220	-7.75523105363049	-0.20613353157865
C	1.82833215676072	-6.91985208251431	-0.14195185009647
H	2.52815444851875	-9.57287988926886	2.65120712557523
H	4.06365021524793	-9.36390992977391	0.70729869601933
H	3.64682097479521	-7.64557747032891	-1.03320862560599
C	-3.38244398895749	4.63336304999172	-4.38951918015636
C	-4.58626055706558	3.96157509974700	-4.28698950496979
C	-4.61729346145125	2.62066210216735	-3.87295828406100
C	-3.44507413286603	1.91906355880775	-3.57315279685445
H	-3.35274727759766	5.68768910621993	-4.67014811809772
H	-5.57091393989379	2.10577853381527	-3.81065105160617
H	-5.52283956945854	4.47409509781320	-4.51030326123136
C	-12.34483592829617	-5.28044253880536	-1.02017790766028
C	-11.46631552702697	-5.98533431166210	-0.24334640695819
C	-10.27939486949118	-5.17753861600830	-0.10188473888825
N	-10.43709516374846	-3.99700317665869	-0.79573251578698
C	-11.72002168218746	-4.02528669396779	-1.32792845511124
C	-11.65931822006273	0.57871827060802	-2.09704544570644

C	-12.52430571401258	-0.47838687921248	-2.31669942991736
C	-11.80857700824191	-1.67534592251732	-2.00182462407590
N	-10.49797979971044	-1.37427365270995	-1.65120148968746
C	-10.39863381641650	-0.00991450968194	-1.71063695398482
C	-12.35560285486375	-2.95021248637216	-1.92703356402417
C	-5.80410055855148	-0.25804762935020	-2.24728826584241
C	-6.76846051706384	0.73707958641341	-2.15485558048104
C	-7.98348895575001	0.13011325863116	-1.70392489594466
N	-7.79381022718017	-1.23321108284644	-1.52887316526577
C	-6.46605648289935	-1.46664483687141	-1.79693766837607
C	-9.23900124817227	0.76480810984815	-1.59363197126030
C	-6.59039764367503	-5.65328435452636	0.30078025043619
C	-5.63060943284712	-4.82250446698985	-0.30943669777773
C	-6.37531154347076	-3.72511846951354	-0.87755048278359
N	-7.72450601919293	-3.85078542136378	-0.63992276560775
C	-7.86593088994150	-5.00722274709845	0.08891562144965
C	-9.06895700783670	-5.58703277939632	0.50114175352983
C	-5.76135845504469	-2.63670857840824	-1.50696981823089
Ni	-9.11565332393806	-2.60666080896314	-1.12830416879527
C	-9.40812285668715	2.21612639372594	-1.50778627114979
C	-8.98600970262640	-6.69211251368156	1.45189478939206
C	-8.39714796750077	3.04884604848326	-1.01836615742179
C	-8.53867411840585	4.44802994865275	-0.99709135680944
C	-9.70256674174363	5.04046018820955	-1.44430894606803
C	-10.79446463568536	4.24069197323828	-1.87175495808965
C	-10.66190944846808	2.81432109258160	-1.87465337623704
C	-10.07669210757453	-7.07815176249550	2.23919168107550
C	-10.00736187749543	-8.19102282066179	3.09446531840086
C	-8.85021819142438	-8.94149468753680	3.16963599125296
C	-7.69599310498545	-8.55592605279894	2.43999822848172
C	-7.74275556611118	-7.38341257946701	1.61912907749450
H	-13.33539606217198	-5.57663965366487	-1.35395724668267
H	-11.58052695036912	-6.99301568686958	0.13894163511221
H	-13.56288855980321	-0.44987011387260	-2.63403491251491
H	-13.38538294250638	-3.08712665607362	-2.25630717825101

H	-6.67836590770924	1.78566483981409	-2.39759445378760
H	-7.48786120328695	2.60333496070663	-0.61863873747108
H	-7.71989310505370	5.06061338503212	-0.61770414320552
H	-10.99138293868086	-6.48868085325410	2.20470115785809
H	-10.87815948812608	-8.46152592294198	3.69290019651220
H	-9.81260096373530	6.12643296483028	-1.44495238841912
H	-8.79921642154622	-9.82810648676707	3.80425462474007
C	-6.49804348025182	-9.31358959490082	2.50305781028573
C	-5.38858878238601	-8.92905992641425	1.77355443083452
C	-5.40931398395330	-7.74297044861722	1.02303811513226
C	-6.54544980174781	-6.92901886163852	0.97068602415473
H	-6.47585089070309	-10.21256225990398	3.12166236866642
H	-4.48193263772737	-9.53519325616018	1.79005087303639
H	-4.53244229504159	-7.46298222572392	0.44761932211776
C	-12.02195791362833	4.82475222084075	-2.27820259818921
C	-13.09276033967904	4.03127595401993	-2.64640069708464
C	-12.98442311371352	2.63121061911123	-2.60444575054393
C	-11.79517586770411	2.01135159906005	-2.21988075257052
H	-12.10788860643177	5.91282421828522	-2.28520513134666
H	-13.84781461802468	2.01607324677880	-2.86145037260474
H	-14.03320658575520	4.48973786314899	-2.95475980127489

Table S8. Coordinates for doubly fused NiDNapP dimer.

	X	Y	Z
C	-4.67077266559889	-3.88585327090684	-0.23685230225742
C	-3.72521895008570	-4.87726488111114	-0.01728058288907
C	-2.49618747650127	-4.44181674168837	-0.60518334019304
N	-2.67341783426330	-3.19997153439422	-1.19625733502294
C	-3.98275689204553	-2.84289848735779	-0.96620044334266
C	-3.06231197984971	1.61053414305268	-1.89635236292688
C	-4.17900119669154	0.86657072615716	-1.55514145300366
C	-3.81848413536627	-0.51101594253240	-1.66565340156779
N	-2.50416793241821	-0.62236553244567	-2.10027280745470
C	-2.04278708461597	0.65913932273122	-2.26189647102481
C	-4.59688583622482	-1.62105670790132	-1.27226229295888

C	1.18677622950059	-0.75483743096377	-5.24616530091318
C	0.78316383602272	0.43912135972209	-4.71333712194200
C	-0.09713905949938	0.13802234123720	-3.60945718163633
N	-0.23503840254666	-1.22781617968322	-3.49001983238842
C	0.58721604538674	-1.79364643854185	-4.45475599640459
C	-0.84152786178180	1.06892120257020	-2.84985247659802
C	0.47539649860809	-5.93453826079652	-2.29622870039597
C	1.02867060777486	-5.40294240000219	-3.44770022163872
C	0.49053478612412	-4.08598813388666	-3.60060195526715
N	-0.43081835164175	-3.82647100395954	-2.59502216819091
C	-0.44775855987218	-4.94085835862670	-1.80011877439075
C	-1.32143954295655	-5.21596389317743	-0.74305917846851
C	0.90381423264371	-3.14057961400446	-4.53383812527356
Ni	-1.44824947383334	-2.21817666761175	-2.32796620885046
C	-0.49815941518240	2.48900501217753	-2.75231122222961
C	-1.03289445436525	-6.39540659241600	0.07319073523885
C	0.80648595005931	2.94619661359294	-2.96162363183198
C	1.12692251346408	4.31490389228752	-2.91087842444975
C	0.15280799122605	5.25224500605543	-2.63201451446217
C	-1.17164896157823	4.83938855417841	-2.33206769564199
C	-1.49594236644000	3.44471963368505	-2.35956058962537
C	-1.58791521621273	-6.56426678110829	1.34570681744307
C	-1.35782086354123	-7.72660326962613	2.10311138715958
C	-0.55306867983679	-8.73452706690331	1.61098387527362
C	0.10368871545267	-8.58097825594930	0.36197257936528
C	-0.10874382178276	-7.38811235832454	-0.40171583765496
H	-3.88746194189543	-5.86390230022750	0.39650741062737
H	-5.13338490830425	1.23166794840970	-1.19617301044521
H	1.84122461911262	-0.92339140832856	-6.09691619050412
H	1.00556710820122	1.43532544056090	-5.07791878850720
H	1.60051703094392	2.22294177776813	-3.13878005910640
H	2.15619103187507	4.62888906845039	-3.08910137090556
H	-2.18877938804393	-5.76175925920258	1.77049788108495
H	-1.81608192017443	-7.82219867048568	3.08830229364765
H	0.39179672449938	6.31698089364922	-2.60742945269122

H	-0.38455092750186	-9.64628776135939	2.18686119619495
H	1.75799836517041	-5.85034502718278	-4.11709449248773
H	1.60806951169466	-3.45462825157374	-5.30386415236369
C	0.98226087261915	-9.57739647001173	-0.13621328420259
C	1.65322852575714	-9.39237957250964	-1.33106575244058
C	1.48766771537795	-8.20284806806607	-2.05953368221983
C	0.62734769512284	-7.19852675172281	-1.61472169163840
H	1.12515315192676	-10.48917556620906	0.44643136423647
H	2.32830580961742	-10.16338355501430	-1.70455750035093
H	2.05119172272384	-8.05294725773899	-2.98138906827623
C	-2.17664882729450	5.77925920175261	-1.98656035928949
C	-3.45107286444455	5.35891690040314	-1.65129706007473
C	-3.76187106638203	3.98966791226860	-1.62906588498180
C	-2.80783677830299	3.02835923383466	-1.96704526160068
H	-1.92253859507671	6.84072550703659	-1.98262371368438
H	-4.76017345311749	3.66745207285738	-1.33003252966914
H	-4.21596631275044	6.08991495977199	-1.38608627799515
H	-13.87868689612541	0.17138225915182	-3.37419760648808
H	-5.06408989497526	-7.22797949313846	3.97670266090262
C	-6.03572600727338	-7.49309618649450	4.39560507816498
C	-13.06210714825238	0.86349538222665	-3.16431357551742
C	-6.10292585252479	-8.33235786156655	5.51946716601411
C	-7.19337866166251	-6.97445290678324	3.81301715331198
H	-5.18344291196905	-8.72688786942073	5.95380363670721
C	-7.16942126578848	-6.05847799858289	2.69923743169971
C	-8.47144529779790	-7.31634568612802	4.35870950514320
C	-10.93966992315609	-7.84979010540271	5.60935297811054
C	-8.52969268557791	-8.13239374537666	5.53424507018445
C	-9.78787744034553	-8.40070429835785	6.13383955033428
C	-7.32600524851169	-8.64042529498125	6.08725129162570
C	-9.68744283044610	-6.83472373395276	3.76486423888951
C	-8.39546059738650	-5.55508424665424	2.13192038803074
C	-10.88870523104476	-7.06724942810617	4.44159590037693
C	-9.64017632742465	-6.05827915293502	2.52425555019737
C	-11.88933524544803	-6.78504647674005	1.56441795209951

C	-11.94515833846269	0.40758689797653	-2.46333441246482
C	-10.74500124229843	-5.90985051893250	1.65543459745851
C	-12.64522205668909	-6.35374183601666	0.50840108721106
N	-8.12628819672479	-4.69182327824102	1.10073014253504
C	-11.99116828944632	-5.19397625303105	-0.03234774858932
Ni	-9.44590026541831	-3.66570678999692	0.14158177754400
C	-6.74392120404868	-4.65593524520739	0.97366346328370
C	-6.14380597923328	-5.51344697694364	1.94551556841577
H	-11.89971255149767	-8.01314803293613	6.10064287246394
H	-9.82306400315380	-9.02180159943354	7.03069576673151
N	-10.80369269914812	-4.96880207762250	0.65068103022769
C	-12.53606584717338	-4.33778625743653	-0.97630760631911
H	-11.80355281317022	-6.60753100467792	4.07181064070750
H	-12.05368440178503	-7.65901167587989	2.18414457070852
C	-6.07368037833225	-3.78043922675975	0.09216849255943
C	-6.73788191910460	-2.66708840001659	-0.43966071832976
C	-6.02360567077346	-1.56708625819370	-1.05036018424872
H	-13.57181531210655	-6.77627739514219	0.12968443701852
N	-8.08582962158575	-2.39361838203557	-0.38311309439755
C	-13.15904718379976	2.19989212495903	-3.58610204916343
C	-6.98564374116657	-0.61105951298368	-1.34305050418149
C	-8.25129108308895	-1.12616330331262	-0.92045023479351
H	-7.38305349771650	-9.27262391349170	6.97516366092699
C	-10.87436398866541	1.31389422246441	-2.17883234257551
N	-10.74637681240972	-2.66674128157385	-0.86197136076511
C	-11.98950566923009	-3.10052017712159	-1.30221480818006
C	-11.00218795732678	2.68652361074655	-2.56765796630243
H	-13.50539293986513	-4.59777546074646	-1.40096891617484
C	-9.51234728017412	-0.53742621493337	-1.16876869801302
C	-9.69062650510773	0.87820669962269	-1.49045181355439
C	-8.76311990328527	1.84617430081145	-1.09244049664630
C	-12.15313504911447	3.10017688769313	-3.28680951391234
C	-8.90383227323913	3.19713266977995	-1.45675671731641
C	-9.98545831173037	3.60976094001727	-2.20885188890352
H	-5.07767856260527	-5.63788438938326	2.09043364030420

C	-10.64063909566475	-1.35957420276662	-1.25391951072656
H	-6.85665265574472	0.30389071467764	-1.90646158162848
H	-7.92831623372638	1.54847095479699	-0.46005417732280
C	-11.83128270921552	-0.93971748020868	-1.95529981213433
H	-8.15320210157637	3.91936795691942	-1.13321287165726
H	-14.04125489947020	2.52831955961041	-4.13701509059395
H	-10.09171755896906	4.65369511986090	-2.50921590023779
H	-12.23395523403967	4.14527983294804	-3.59096140019456
C	-12.65322983187739	-2.05150319711668	-2.01420242142408
H	-13.63330195821056	-2.14483830815734	-2.47344028307252

Table S9. Coordinates for triply fused NiDTP dimer.

	X	Y	Z
C	-2.04870598334067	-4.53572555249119	2.17335536815125
C	-1.05264384478607	-5.47955457222012	2.22390645847486
C	-0.07031917283765	-5.05920432244960	1.26357088061231
N	-0.40180277367004	-3.91406906145103	0.62555552252241
C	-1.63465372682498	-3.56261248936454	1.17349667657342
C	-1.86374974717319	0.26169701543169	-1.52668100189517
C	-2.60344833495887	-0.37368605628189	-0.56911521654978
C	-1.87105831480062	-1.54629323499281	-0.17697496077344
N	-0.68502393193592	-1.63983095007483	-0.88580468241403
C	-0.67670951632019	-0.51875147227369	-1.72455943226179
C	-2.32874631665971	-2.43663010038150	0.78771314372018
C	3.43123480554497	-1.69136748614159	-3.56243841295580
C	2.43346536601887	-0.72481364004961	-3.62049678413044
C	1.44549115444389	-1.13456608934182	-2.66317235654774
N	1.75599933573173	-2.28418972946694	-2.01337179023295
C	2.97217306542150	-2.64301445808940	-2.55005749604874
C	0.37050683559362	-0.26158988655690	-2.60311827724531
C	3.24067442343918	-6.46345527953035	0.12725356808984
C	3.98788952972585	-5.82456057231560	-0.84542646800699
C	3.22815273644991	-4.64926724789938	-1.22034576723706
N	2.05559003238983	-4.55817058056363	-0.51258498956461
C	2.05953067548582	-5.68646903579377	0.32817997848113

C	1.01028481291095	-5.93338139937286	1.20513145954435
C	3.69948981937478	-3.76315391070634	-2.19471295006866
Ni	0.67786281369384	-3.09815493078769	-0.69538322250793
C	0.67148659718387	0.80569731614614	-3.59248478551523
C	0.72035225301803	-6.99989470150255	2.19297399855257
C	1.93954664321379	0.51321422171769	-4.21163752625832
C	2.46305161827866	1.36025429241864	-5.18080874131679
C	1.75775456321315	2.51641593876960	-5.57008475507439
C	0.52635574769925	2.78930090072502	-4.96208809033145
C	-0.02017154131023	1.94595690808357	-3.97957423554760
C	-0.54681183465348	-6.71876331070835	2.81704613345220
C	-1.06034621475910	-7.56485972543181	3.78693412907762
C	-0.34097294211817	-8.71711975860414	4.17458434627673
C	0.88818671806841	-8.98047683305928	3.56266159995186
C	1.42447250622092	-8.13361742048860	2.57675486703017
H	-2.97760275046334	-4.46803468907192	2.73362905509448
H	-2.10117413065533	1.17944763391985	-2.05330740598682
H	-3.56491880820113	-0.08473775550788	-0.15348557480042
H	-3.28786027923040	-2.22486849466964	1.25935459068336
H	3.48289829988811	-7.38093773446110	0.65133380204332
H	3.42487929015650	1.13493543480482	-5.64500505415008
H	-0.02591841249312	3.68323110687343	-5.25915808385243
H	-0.98109100191656	2.20143887849190	-3.53442367130445
H	-2.02232021927906	-7.34415363656707	4.25486947134878
H	1.44717787073564	-9.86988450439918	3.85978184694242
H	2.38601858761377	-8.38135170038346	2.12892921859861
C	5.24347112071507	-6.08227596501546	-1.48557834098070
C	6.24151550048316	-7.04840782970065	-1.42692225725631
C	7.22960483257391	-6.63909990905893	-2.38413919586645
N	6.91888264803881	-5.48973724865340	-3.03456599814448
C	5.70253929676759	-5.13108156264390	-2.49822192694591
C	5.43360002441795	-1.31054333670353	-5.17551345742864
C	4.68670089050106	-1.94920614785819	-4.20275447631412
C	5.44661573006853	-3.12482710286495	-3.82789459601493
N	6.61898095547385	-3.21594607989009	-4.53588338121515

C	6.61490726520456	-2.08769796143881	-5.37680435272585
C	4.97517409051132	-4.01077796709697	-2.85376136913764
C	10.72328143856975	-3.23736370770402	-7.22137845511667
C	9.72644673703233	-2.29437510836780	-7.27250291512103
C	8.74440867893025	-2.71528946829192	-6.31210219457393
N	9.07653814566670	-3.85969909805758	-5.67353169843298
C	10.30987480256159	-4.21052011999131	-6.22104962898202
C	7.66362502908649	-1.84098434594172	-6.25392545831109
C	10.54137418656596	-8.03206754144208	-3.51730471691579
C	11.28109745397540	-7.39664442786385	-4.47511663553669
C	10.54770050367559	-6.22549937316502	-4.86870679104857
N	9.36103462812159	-6.13264331313046	-4.16073324728324
C	9.35353711158035	-7.25289384190311	-3.32110688309780
C	8.30565260916787	-7.51072814988175	-2.44297644521613
C	11.00472765442892	-5.33533575088152	-5.83413956012828
Ni	7.99715875356371	-4.67564128729077	-4.35263658277361
C	7.95453607411428	-0.77483903567722	-7.24236724776873
C	8.00665307169146	-8.57748259885646	-1.45315360409093
C	9.21948551857307	-1.05543519244871	-7.86499241898706
C	9.73360690858740	-0.20718277239749	-8.83636705181954
C	9.01644487919797	0.94265793202846	-9.22322342563618
C	7.78427902185982	1.20646825462715	-8.61043083261558
C	7.24939794662318	0.36066299680563	-7.62692946470457
C	6.73578271079772	-8.28531413957406	-0.83342281759534
C	6.21365624719914	-9.12953765122512	0.13527467556834
C	6.92266047396522	-10.28668104172972	0.52713324222884
C	8.15156414711035	-10.55835993136315	-0.07930134991930
C	8.69869535241860	-9.71459874710958	-1.06514931883748
H	5.19110010284190	-0.39315446031546	-5.69965176258744
H	11.65240341166531	-3.30448465730415	-7.78135653246809
H	10.77953000488721	-8.94901110625845	-2.98964158298212
H	12.24316758970039	-7.68492529486243	-4.88981068170125
H	11.96428138529321	-5.54653633858115	-6.30517863199449
H	10.69538028659500	-0.42668946035435	-9.30456580738502
H	7.22419192951498	2.09580739817816	-8.90681886721127

H	5.25129765645623	-8.90331284457673	0.59860629581342
H	8.70372115039116	-11.45124711956838	0.21997476325007
H	9.65995094350448	-9.97045878031089	-1.50925516010582
C	9.56335548388090	1.87207398278639	-10.27567247699001
C	2.31837952260211	3.44110369358126	-6.61974086105520
C	6.35214404122732	-11.20094909938786	1.57928878654676
C	-0.89884029930777	-9.63694609142791	5.22849230486365
H	6.28767064988097	0.60712028310575	-7.17864353038923
H	-0.22515807158933	-10.48271832638344	5.41687904452813
H	-1.87657246759305	-10.04300763151852	4.92631392215013
H	-1.05160058219105	-9.10617639720478	6.18091996564497
H	3.28703853768328	3.08213938292686	-6.99167795205416
H	2.46604457355033	4.45619468974120	-6.22082660317882
H	1.63844348723085	3.52800629676519	-7.48086683362703
H	10.53344180770566	1.52252247227335	-10.65249693953640
H	9.70266282072397	2.88868314729087	-9.87743902035293
H	8.87787592830340	1.95258390591131	-11.13310573622011
H	7.01807959207000	-12.05179937647565	1.77211223697488
H	5.37304273466150	-11.60014528376175	1.27224327144970
H	6.19938903736323	-10.66790173644042	2.53049418471987

Table S10. Coordinates for doubly fused NiDTP dimer.

	X	Y	Z
C	-3.32411100328174	-3.86550156370401	-0.82365648332939
C	-2.46747008680594	-4.87553544551528	-0.48395102000873
C	-1.15721258564296	-4.47531960228818	-0.91175134420093
N	-1.21078083937229	-3.21624170771376	-1.51570664935513
C	-2.53901841422300	-2.83475473897641	-1.44617337949422
C	-1.58405817555576	1.47842698771308	-2.96927801405706
C	-2.71461676758990	0.79167034014947	-2.59762823501659
C	-2.28716814106112	-0.57245207714811	-2.31588990882763
N	-0.91826506286537	-0.71000423223806	-2.54054434098314
C	-0.51758021667914	0.51708536300959	-2.93036569233502
C	-3.06377915274151	-1.60467272833586	-1.83513039291080
C	3.79725647066018	-0.58784395953175	-4.16610976047729

C	3.08189084291221	0.57391226573004	-4.06321440083637
C	1.79936312376869	0.23026830814404	-3.52340869903703
N	1.74572314103424	-1.14376007758977	-3.25038514005979
C	2.96098381259999	-1.64633548165261	-3.67392405867971
C	0.69426848282183	1.05387328320208	-3.35561462516236
C	2.27163370593183	-5.64917450601007	-1.35187638103333
C	3.29539939968968	-5.07109633251115	-2.10601277035656
C	2.73580848626318	-3.81150451088009	-2.60857029577986
N	1.47276814471832	-3.58795520673416	-2.11114283866829
C	1.19480243558031	-4.70273607994439	-1.38834339849652
C	0.02960310565151	-5.19507084010050	-0.81441809638822
C	3.40301534817371	-2.97448406666008	-3.50346757683375
Ni	0.26320531367407	-2.17129154921383	-2.36452761594215
C	0.41669095770728	2.48339599567270	-3.64249217161070
C	0.37167518454163	-6.55337543646125	-0.32679201162567
C	1.23106626102968	3.52597978748653	-4.06431068685913
C	0.67565512370326	4.80324513368737	-4.25759910171121
C	-0.68113479597209	5.06088052878295	-4.03932834442635
C	-1.51250280770275	4.00356353842059	-3.60843591758438
C	-0.98202357308428	2.73870242594473	-3.40975982372952
C	-0.38248736991505	-7.52340144525503	0.32118950683894
C	0.18639821156785	-8.77761111895022	0.59551288163617
C	1.49709334402652	-9.08746050359572	0.21515676269833
C	2.27053884068252	-8.10074761113552	-0.42842136593579
C	1.73802255421311	-6.84061535569247	-0.68053120053957
H	-4.39623523916246	-3.80445012812054	-0.65764047653639
H	-2.70418722378232	-5.81315690886733	0.00606990919857
H	-3.74096195354537	1.13633072263552	-2.50162905503185
H	-4.13204530728337	-1.44052356032902	-1.69753880254301
H	4.82188408519844	-0.71668643415360	-4.49583261880260
H	3.40328562658305	1.57569769344871	-4.32550059566359
H	2.29507468900466	3.37532208285617	-4.24280280327895
H	1.32262074167692	5.61836235650815	-4.58747043727650
H	-2.57448632315937	4.18743989373622	-3.43132534846702
H	-1.41203517068598	-7.32914971245253	0.61924085194078

H	-0.41456209634088	-9.53317381838716	1.10581766098429
H	3.29090289307095	-8.33577128846695	-0.73079270832258
C	5.18917968360006	-6.78429914336113	-0.43554438984324
C	6.23314529232564	-7.58233147357604	-0.05519042317033
C	7.11775779062853	-7.67952254922773	-1.17912735263434
N	6.63567001025586	-6.89842495028821	-2.23879092082202
C	5.43785761310215	-6.37607007718362	-1.78992014211969
C	5.34178962079481	-3.24351157397252	-5.26373091583575
C	4.57306510784425	-3.53887376206946	-4.13545162639831
C	5.17771239926756	-4.74847667532138	-3.56679497707395
N	6.30934866867250	-5.11762453780380	-4.25635464508738
C	6.38055909766376	-4.23182597961910	-5.28244126496877
C	4.63965729188834	-5.44232798457223	-2.48220268232981
C	9.63262837553084	-6.47263637473197	-7.70028889751403
C	8.88503240339458	-5.34199056224451	-7.88138233574990
C	8.09144271601343	-5.17065851027156	-6.69817985340004
N	8.35238943057876	-6.19930851380229	-5.78919984723981
C	9.31179261792597	-6.98889283079508	-6.39796760906787
C	7.14905329687559	-4.18011579105666	-6.43832738230140
C	9.95578281495535	-9.53182122543996	-2.53015413651555
C	10.47095607750744	-9.52650833402683	-3.80392653466878
C	9.73897089296246	-8.49960201118264	-4.53419617556971
N	8.77693108838848	-7.91139495338516	-3.71436008967584
C	8.91837797209529	-8.53811305499156	-2.52912925124878
C	8.24610799919580	-8.47715342954283	-1.31162615294353
C	9.95894887244829	-8.08362557484569	-5.82927016836125
Ni	7.51338985122608	-6.54077519184626	-4.01042357431994
C	6.60496146125146	-3.04798659499155	-7.22615157472997
C	8.92592442880731	-9.46285565690092	-0.43405725296585
C	6.95924376041970	-2.52497789794238	-8.46081941644819
C	6.19858751859654	-1.48047482989357	-9.01772067915353
C	5.07716096472371	-0.96386202507707	-8.36554304407664
C	4.72171598216247	-1.48364755002687	-7.10089936163692
C	5.47826380711712	-2.49108140940225	-6.51732350269527
C	8.72579126207022	-9.82475268203020	0.89373160400861

C	9.53890249466044	-10.81172140863362	1.47123731772926
C	10.55404550338474	-11.45205845523314	0.74783610306611
C	10.76127078074908	-11.08641907807807	-0.59704966071109
C	9.96798363680314	-10.10985571714332	-1.18464377242568
H	4.34721126603187	-6.45301236024794	0.16153617535958
H	6.38900492500288	-8.05508724150042	0.90809194192876
H	10.35519471469281	-6.92046054557075	-8.37709045935739
H	8.86571452189452	-4.68741632780973	-8.74526108126900
H	11.26499939347264	-10.12798561486761	-4.23878391712336
H	10.71286186175859	-8.59537771705412	-6.42661379860673
H	7.81570500845575	-2.91257221239845	-9.01080399581362
H	6.48376240922722	-1.07379531272974	-9.98981898339114
H	3.84445741647947	-1.08872148484158	-6.58816690606227
H	7.95267024612177	-9.35083230490260	1.49732471801855
H	9.37565658364348	-11.08850576495874	2.51491245400760
H	11.54962740067395	-11.57412397252284	-1.17410122789743
C	-1.25767124867038	6.43538083001586	-4.25365732636763
C	4.24801085720765	0.13133946077190	-8.98243057171200
C	2.07759420266251	-10.45073358303630	0.48939711757119
C	11.40782094090151	-12.50915783149678	1.39879876061346
H	12.15263690812622	-12.90919445488689	0.69841573460623
H	10.79644678352795	-13.35092738792815	1.75822363654316
H	11.94608173860253	-12.10623973471920	2.27023939873089
H	4.64470241517500	0.43099700222178	-9.96110193115645
H	4.22253211996329	1.02302752128478	-8.33734746073603
H	3.20495693274538	-0.19158278662341	-9.12256348967111
H	3.11190029784381	-10.52295501190044	0.12876141963173
H	1.49160148643054	-11.24126652474263	-0.00366526168896
H	2.07893580078369	-10.67459208279516	1.56718076965157
H	-0.48855608605054	7.14438156135049	-4.58639365135622
H	-1.70369382485972	6.82921429139669	-3.32730441440395
H	-2.05481167776540	6.41925928249092	-5.01292604623245

Table S11. Coordinates for triply fused NiDOMePP dimer.

	X	Y	Z
C	-2.01971041302165	-4.49267556304817	2.22470664290339
C	-1.02143785134285	-5.43362855281791	2.28989851360725
C	-0.04215862544885	-5.03087497925553	1.32001736407469
N	-0.37731427508696	-3.89866454844900	0.66183030012413
C	-1.60999277621970	-3.53848408706141	1.20630314138971
C	-1.85533360217087	0.23585203317263	-1.56329845762889
C	-2.59059206166055	-0.38377765994847	-0.59198866890406
C	-1.85408698286401	-1.54723372226610	-0.18068631161613
N	-0.66995442786168	-1.65102528832343	-0.89114352505199
C	-0.66700082441712	-0.54473506734459	-1.75071881987417
C	-2.30709461536124	-2.42084097814250	0.80151827449474
C	3.43683770562999	-1.74534461026601	-3.57543200171664
C	2.43625136727537	-0.78296873731526	-3.64840967827679
C	1.45230674879223	-1.17522636344093	-2.68202384507555
N	1.76718235368513	-2.31155287507593	-2.01191149683379
C	2.98314388217080	-2.67837337899309	-2.54497721505323
C	0.37635862075485	-0.30022339945754	-2.63738851251977
C	3.26943316811900	-6.44763001931153	0.20137949596381
C	4.01272382186466	-5.82428350702408	-0.78551286571292
C	3.24889731712012	-4.65923877026418	-1.17964558206865
N	2.07787883666638	-4.55803747069432	-0.47044385649477
C	2.08713134593666	-5.67097638936225	0.39100530681019
C	1.04047289511999	-5.90553378899465	1.27586449213124
C	3.71488651870638	-3.78965544594946	-2.17185325369031
Ni	0.69607309831938	-3.10393263433865	-0.67657862512311
C	0.67435012673642	0.74710780677713	-3.64675589488837
C	0.75614341113045	-6.95202125659118	2.28146001279821
C	1.93906047720051	0.44262258463773	-4.26180165287179
C	2.47630522723661	1.25875020548617	-5.25104259929008
C	1.75632695042872	2.40367395836644	-5.64875216513688
C	0.52382981564580	2.70799090316999	-5.05463380714496
C	-0.01478850039682	1.88373151484897	-4.05833901281177
C	-0.51233660880559	-6.66058029609900	2.90495502837733

C	-1.02608504493901	-7.48260288985438	3.88941991128718
C	-0.29029117989125	-8.62295015377238	4.28421771030519
C	0.94079822252193	-8.91745869879660	3.68735456228693
C	1.46009367353879	-8.07766343307216	2.68516658556674
H	-2.94694310100736	-4.41661040854480	2.78660953602116
H	-2.09651881986971	1.14304250780097	-2.10647605836148
H	-3.55163473540961	-0.08984840770304	-0.17881864719422
H	-3.26528821653223	-2.20240467673326	1.27198390458022
H	3.51561237045476	-7.35461079593379	0.74178868882372
H	3.43434303019168	1.00883060769060	-5.70145587743746
H	-0.00435294730747	3.60120530012556	-5.38734181558567
H	-0.97440610305074	2.15101284833946	-3.61754765172336
H	-1.98218377289093	-7.27944759027373	4.37211964730668
H	1.50967922719905	-9.79449543681688	3.98848818976375
H	2.42025318033142	-8.33325722328753	2.23912184572559
C	5.26746516304666	-6.08985235229619	-1.42425948589702
C	6.26983440193311	-7.05080307612771	-1.35083616064357
C	7.25331430625214	-6.65809118842314	-2.31807415837082
N	6.93651475273032	-5.52250378045337	-2.98953966112775
C	5.72058512913142	-5.15726027578860	-2.45615225249739
C	5.43360166071133	-1.38576179241158	-5.19991353789478
C	4.69100114552612	-2.01011750276621	-4.21449569174866
C	5.45536173050610	-3.17638299535420	-3.82144572022901
N	6.62620182370969	-3.27631429080929	-4.53088457212868
C	6.61661829286191	-2.16200155202110	-5.39100726361347
C	4.98837340654081	-4.04564837493743	-2.82986609963074
C	10.71857294501212	-3.33700341102305	-7.23278977922174
C	9.71954692526168	-2.39568530565901	-7.29449066036811
C	8.74358163238917	-2.80094891605676	-6.32300813176647
N	9.07984368141613	-3.93335465529209	-5.66723574364993
C	10.31171746919084	-4.29277718415939	-6.21453265711720
C	7.66107170505111	-1.92582016972732	-6.27563977522690
C	10.56347801714722	-8.06265791592918	-3.44052242948206
C	11.29700148108078	-7.44408904441430	-4.41471535383925
C	10.55916476894017	-6.28284249844776	-4.82649821373509

N	9.37550722156125	-6.17918743735942	-4.11379276718311
C	9.37523295424848	-7.28316735364515	-3.25296136601542
C	8.33217120466831	-7.52864161253914	-2.36397490653388
C	11.00961228703239	-5.40976975231145	-5.81062283164482
Ni	8.00752477948664	-4.72907644946724	-4.32703668745640
C	7.94589152688270	-0.87789573980931	-7.28049633908430
C	8.04202315121407	-8.57551949988097	-1.35507292736758
C	9.20684621045977	-1.16719743196875	-7.90449132811667
C	9.72880057675051	-0.34336328587489	-8.89461580696461
C	8.99312208779884	0.79540507916166	-9.28208995399866
C	7.76101128762817	1.08612153846648	-8.67748242924406
C	7.23968233094265	0.25449744255821	-7.68217547266587
C	6.77030346958502	-8.27446872189366	-0.73521059715631
C	6.25060526874004	-9.09453532325774	0.24962691893393
C	6.97823936156100	-10.23818381244252	0.64943403762954
C	8.20848758240435	-10.53992510074349	0.05757762285846
C	8.73587954748209	-9.70292818647362	-0.94627454466588
H	5.18756450062615	-0.47846506982530	-5.73992275983400
H	11.64494026535277	-3.41304138462083	-7.79635210157092
H	10.80692099049564	-8.96871597243442	-2.89659075847499
H	12.25762775818439	-7.73811587215483	-4.82879827511182
H	11.96721629887496	-5.62835951582674	-6.28237277020554
H	10.68614334639243	-0.58216005101350	-9.35400812066887
H	7.22330155552248	1.97561285099329	-9.00492352520438
H	5.29434352167686	-8.88669120083541	0.72861932960475
H	8.77195848455591	-11.41935973356321	0.36185771200699
H	9.69596551271117	-9.96431034811500	-1.38959300701933
H	6.28083297760080	0.50877508232362	-7.23228348213634
O	-0.87475331182601	-9.37595580123172	5.26358063356563
O	6.38427958460412	-10.98543025910767	1.62906873862610
O	9.40464871369159	1.67663380464481	-10.24260222106490
O	2.18923898444832	3.27478271558947	-6.61017626130407
C	3.43802853734709	2.99544451437206	-7.24055706516676
H	3.59253015635910	3.80086869345867	-7.96642789682709
H	3.41428591203513	2.02705998191101	-7.76728849946585

H	4.26542687245372	2.99593613168113	-6.51164728220658
C	10.65008734091228	1.41832833192941	-10.88914606218317
H	10.78535265579844	2.23136663208737	-11.61034777459809
H	10.63290119373498	0.45354174855652	-11.42213735419874
H	11.48492018861888	1.42344027680150	-10.16922835723978
C	7.07433030814113	-12.15137315093092	2.07492717088138
H	6.44115558966806	-12.59590069506938	2.85025100536670
H	8.05698903672076	-11.89463710748396	2.50354673059602
H	7.21232853556969	-12.87317401696173	1.25314948662006
C	-0.17702817881923	-10.53930248145518	5.70497177101317
H	-0.80604831909263	-10.98828784962975	6.48102258633973
H	0.80538729416202	-10.27809314583848	6.13134471106347
H	-0.03762550062749	-11.25848634446374	4.88119916940664

Table S12. Coordinates for doubly fused NiDOMePP dimer.

	X	Y	Z
C	-3.34600060763782	-3.90168282371927	-0.83333528485340
C	-2.48152132652165	-4.90053984582558	-0.47996603939912
C	-1.17198797973766	-4.48866687821503	-0.89749877816703
N	-1.23352620279999	-3.23335041553325	-1.50976259529582
C	-2.56639946525745	-2.86618799523779	-1.45461869848489
C	-1.64542264554750	1.44771222001496	-2.99448553315351
C	-2.77203789366018	0.75112757303399	-2.62802758687625
C	-2.33232384874854	-0.60586404778630	-2.33615791180157
N	-0.95937461851313	-0.72943784387470	-2.54974033153229
C	-0.56887411722252	0.49906233651717	-2.94295591056410
C	-3.10117090627688	-1.64395040917358	-1.85553391077571
C	3.76716526215785	-0.56803235677626	-4.13727437051334
C	3.03908800186068	0.58717781698986	-4.04673807318279
C	1.75634813791708	0.23393998051032	-3.51523570254116
N	1.71453997768630	-1.13961196917891	-3.23492751294347
C	2.93810361860613	-1.63186041769646	-3.64649650004851
C	0.64158785460368	1.04790015832813	-3.36180510791803
C	2.27169539517034	-5.62250108791319	-1.30063684082593
C	3.29267584421649	-5.04326937791171	-2.05803217476715

C	2.72540730286953	-3.79277591608229	-2.57110924696086
N	1.45742551376663	-3.57816237669978	-2.08080704368300
C	1.18653413946222	-4.68912616839223	-1.34969776446720
C	0.02133841690146	-5.19548303513013	-0.78327728537582
C	3.39154285170716	-2.95540024283724	-3.46650618359626
Ni	0.23559781036746	-2.17717255371852	-2.35457538354904
C	0.35232606598399	2.47006461086179	-3.65676046994132
C	0.37459415401144	-6.54537497553102	-0.28668518279873
C	1.15561560571393	3.52051198952799	-4.07671833087920
C	0.60284877751747	4.79778537028568	-4.28381003725984
C	-0.76283097397669	5.01769884373850	-4.07030512952962
C	-1.60109313770111	3.96434550767630	-3.64161537162209
C	-1.05365359980143	2.71217593723568	-3.43569722749570
C	-0.36935317577329	-7.52939069209392	0.35838431727075
C	0.20775773113682	-8.77167224330684	0.64670519131050
C	1.53105063116729	-9.04327003417037	0.27458725573115
C	2.30985967304767	-8.06068096343596	-0.36937697490893
C	1.74566032839174	-6.81585313087873	-0.62747054404287
H	-4.42019909556026	-3.85124570812723	-0.67732605448805
H	-2.71236374837096	-5.83781462096949	0.01374843206005
H	-3.80242107277931	1.08608695968774	-2.54171567582819
H	-4.17228498954818	-1.49070195622164	-1.72767931323636
H	4.79597384278330	-0.68776456545446	-4.45719919737405
H	3.35199040917052	1.58959877868242	-4.31673065528154
H	2.22218813324878	3.38016145753667	-4.24770390244028
H	1.25180527152128	5.60728342866911	-4.61129849714931
H	-2.66051312988960	4.16549644319179	-3.48199171873817
H	-1.40455883377295	-7.35183135313184	0.64698351445876
H	-0.36221477491362	-9.55042258324558	1.15284083933162
H	3.33638318774681	-8.26399229957757	-0.66218904988184
C	5.19372819430384	-6.73812703588243	-0.37764703659036
C	6.22935860194080	-7.55069830174680	-0.00181864450887
C	7.10033980489445	-7.67028958380653	-1.13364365089884
N	6.62074983259114	-6.88580224089439	-2.19320654762180
C	5.43418883614698	-6.34533821916107	-1.73759417917508

C	5.34226660502577	-3.22008166176186	-5.21704901844612
C	4.56762628890155	-3.51526560218796	-4.09150351595667
C	5.17227178440674	-4.72402955029177	-3.52085816676871
N	6.30375958863836	-5.09639129878007	-4.20951224450263
C	6.37817904902275	-4.21045453758812	-5.23501150914944
C	4.63612068841723	-5.41276133136349	-2.43240955246841
C	9.60881196278245	-6.47725189138725	-7.65954118845364
C	8.87280016965553	-5.33775342265315	-7.83596791120603
C	8.08204865816390	-5.16201362468622	-6.65234381681896
N	8.33286106861501	-6.19602107839882	-5.74682962937466
C	9.28295803266644	-6.99459094308367	-6.35978757004479
C	7.14697272772347	-4.16297229011554	-6.39238671879899
C	9.89408131860324	-9.56918960886758	-2.50799022906435
C	10.40688383739822	-9.56501869161746	-3.78335466567387
C	9.69093407399573	-8.52219693516223	-4.50507731414231
N	8.74078610553303	-7.92256675141807	-3.67838398879688
C	8.87483696212836	-8.55817060814357	-2.49765965322847
C	8.21091818641041	-8.49086619591766	-1.27434243968566
C	9.91543012977258	-8.10126909063717	-5.79762043622600
Ni	7.49404406315181	-6.53403201271258	-3.96796991413570
C	6.61178088883782	-3.03172970386384	-7.17969470107875
C	8.87371647502079	-9.49365410116411	-0.40768892509465
C	6.96701867683068	-2.51125972508409	-8.41400833581391
C	6.22336715994088	-1.46247315215276	-8.98734745408594
C	5.10795792229654	-0.95416661358247	-8.31572760247890
C	4.73314644451313	-1.45936699299197	-7.04977944971855
C	5.48354240493694	-2.46876216919821	-6.47148651912557
C	8.67141806069978	-9.86703526557905	0.91969276660458
C	9.45909315425542	-10.87116732911939	1.49033144950711
C	10.45612884263462	-11.51429044332333	0.74152197379576
C	10.68331880148949	-11.15525803378047	-0.60262760158811
C	9.89930879587288	-10.15498834270027	-1.16567567534205
H	4.36498348449717	-6.38559698199799	0.22595699267885
H	6.39332380728761	-8.01042166944011	0.96666856419173
H	10.32599605244470	-6.93040008708648	-8.33855223534447

H	8.86009826942032	-4.68052509498796	-8.69796117796032
H	11.19027101587090	-10.17634636123935	-4.22398171959327
H	10.66088648596007	-8.62052254038357	-6.39918265307967
H	7.82264217753842	-2.90346660693487	-8.96189175857096
H	6.52073779138682	-1.06892373662408	-9.95696418315546
H	3.85506279863511	-1.03979227248820	-6.56200747591855
H	7.90952398385166	-9.38497418069263	1.53094593527603
H	9.31581895925906	-11.17389773341338	2.52738595588314
H	11.45326038454754	-11.64342789885550	-1.19715832638358
O	-1.38903845285542	6.21989295351492	-4.24685127907861
O	4.30341206469000	0.04193833596429	-8.79407753316979
O	2.00018192632746	-10.28993290506897	0.57964834024043
O	11.16190804990277	-12.48187033101853	1.40092018322679
C	12.18765668907656	-13.16134419458837	0.67868249551336
C	-0.59055043144633	7.32158223345654	-4.67528059651383
C	3.34521110342914	-10.59640431508551	0.20629104458582
C	4.62735708371263	0.59397804637988	-10.06835434984657
H	12.61853847454030	-13.88285004881985	1.38101331156554
H	12.97178253074036	-12.46327935485917	0.34213661646841
H	11.77721561823783	-13.69712795212909	-0.19301369439916
H	3.51801423484297	-11.62624960731733	0.53618232260421
H	4.06366176161340	-9.92324995914573	0.70155860294243
H	3.48498190460897	-10.53058793949399	-0.88472807920133
H	3.87081482041061	1.36172773369740	-10.26262077120417
H	4.58606310289470	-0.17323575129397	-10.85903630161773
H	5.62860160849169	1.05583171722988	-10.06174957411517
H	-1.27441286840228	8.17347775803234	-4.75384687443863
H	-0.13180218301633	7.12507560364363	-5.65831499821174
H	0.20104575855899	7.55047899721054	-3.94294161320754

Table S13. Coordinates for triply fused NiDMP dimer.

	X	Y	Z
C	-1.83221911991075	-3.46014097169775	2.45266840456404
C	-1.23501365323020	-4.68392141171874	2.36173482610864
C	-0.25713744804290	-4.59444351989350	1.30758101922730
N	-0.28416891979208	-3.33520858224825	0.72692495161443

C	-1.25724044109991	-2.63724278658385	1.42193245825054
C	-1.37565569149240	0.83610718113112	-1.68875119519053
C	-1.93487460204294	0.53600659306900	-0.48043093476098
C	-1.30767159022175	-0.67406626834339	-0.01912393582783
N	-0.34176492640904	-1.09536062013888	-0.91751784693344
C	-0.36968571057804	-0.16361205629850	-1.94380403385577
C	-1.70437403828682	-1.36718911885096	1.10908279507430
C	3.60120325464486	-1.76957704238455	-3.78462461551267
C	2.64227510993886	-0.82841819741578	-4.08971365180477
C	1.59418712046178	-0.97053167009520	-3.11688146546960
N	1.87453746422481	-2.01236442860622	-2.23692518007312
C	3.09077224904230	-2.50174934094848	-2.64753177514646
C	0.50548218663615	-0.11125996946718	-3.02445144317638
C	2.78588822102743	-6.33765605818456	-0.03977104243765
C	3.70487542402443	-5.73282705594574	-0.86901098436472
C	3.14125307482308	-4.44789047986335	-1.21592009942338
N	1.93134225962706	-4.24400378148584	-0.59801283133193
C	1.70838553534692	-5.40438907289708	0.13932215278063
C	0.64492151181814	-5.61111781073222	1.00998616328868
C	3.75260650802785	-3.62184782834610	-2.15431061501767
Ni	0.79295328731727	-2.67171993415922	-0.75575762451798
C	0.32026188577457	0.94109026113819	-4.06563204226834
C	0.52033714321807	-6.92740452634402	1.70428935384331
C	-0.46819507173673	0.65976929812743	-5.19666924365275
C	-0.63151257089037	1.65307317299349	-6.17059870345885
C	-0.03357643574910	2.91081012774471	-6.04854862953078
C	0.74685670627569	3.16503176812034	-4.91336577808243
C	0.93520345034731	2.20146794200058	-3.91751881045626
C	-0.23787443052406	-7.95653602454756	1.11208618182517
C	-0.34015095160913	-9.18842790406752	1.77148489698187
C	0.28501644273726	-9.42373957540009	2.99842752675087
C	1.03175233469210	-8.38522934570381	3.56739245778261
C	1.16351216382732	-7.14093828895293	2.94281065858118
C	0.16398787944775	-10.75559669862047	3.69398045929018
C	-0.21950378881528	3.96801782093838	-7.10685326321408

H	-2.61316102107658	-3.13415994583295	3.13422823879476
H	-1.40738308940275	-5.57389577615623	2.95834676077098
H	-1.59109259611452	1.66562906960567	-2.35492811302259
H	-2.72115731101319	1.06211845645499	0.05398422402384
H	-2.47889809015512	-0.92646063425235	1.73582971624077
H	2.64133731010711	-0.08734523564158	-4.88296210507359
H	2.83232251775218	-7.31444266417373	0.43087371903997
C	-1.12105821560206	-0.68846573291697	-5.35828637251463
H	-1.24339751352560	1.43492231487158	-7.04966462821274
H	1.22409109285166	4.14187059727267	-4.79778924860759
C	1.78205012177929	2.50273583560029	-2.70851734899651
C	-0.93803061206395	-7.76596141516751	-0.21095229247166
H	-0.92735539469720	-9.98679460935649	1.30953913873694
H	1.53013713803286	-8.54828955533748	4.52729729136853
C	1.98632245085169	-6.06346018878618	3.60618670725685
C	4.97391609866343	-6.11009691666490	-1.42666198500138
C	5.77731206492459	-7.22650725775167	-1.35391920865095
C	6.93712392869439	-6.95873299405740	-2.15841200829913
N	6.84464920842201	-5.70540264761369	-2.75896353856846
C	5.64797106446347	-5.19365975270038	-2.31854571651026
C	5.63762755887095	-1.71342198289575	-5.40058771794736
C	4.87121874836865	-2.14592356701552	-4.34088281401110
C	5.59774771022715	-3.24695575740167	-3.74899394711161
N	6.78855907634382	-3.47216434180379	-4.39634150456990
C	6.82712651006520	-2.51944683872842	-5.41135165936294
C	5.04496435333637	-4.00673267373569	-2.72280572939719
C	11.30661058407047	-3.41180741276652	-6.31907058857884
C	10.30774424846001	-2.63114942317907	-6.82443467973819
C	9.10605281152836	-2.97394283991731	-6.10721630530351
N	9.35809903826835	-3.98670581265809	-5.19417713248239
C	10.70834424893518	-4.26420166381750	-5.32619103117561
C	7.90197597849641	-2.29474053773209	-6.26297568213704
C	10.44034984847745	-8.16593321426355	-2.78259398570538
C	11.40277779574062	-7.41565670365819	-3.39346611765562
C	10.75600681542280	-6.22963227102468	-3.88835386162745

N	9.41244562438032	-6.23005699069143	-3.55309975356217
C	9.21268898266245	-7.41640468367156	-2.86281074347610
C	8.03393381097572	-7.80843079554299	-2.23633972183949
C	11.38067558453075	-5.28338572677916	-4.67875107682533
Ni	8.10250366454574	-4.84941266608748	-3.97668352130826
C	7.78730696428634	-1.23750026425785	-7.30957378652538
C	7.97292658315311	-9.13639239888550	-1.55598016365174
C	7.34555643586047	-1.58511203148603	-8.59944924666208
C	7.23160229247836	-0.58008029120222	-9.56853795575943
C	7.54383891967347	0.75335349076056	-9.28795683935537
C	7.98411124218063	1.07223771815783	-7.99683773468643
C	8.11224972659232	0.09968743455469	-7.00033170058377
C	7.53726493768190	-10.26741931771143	-2.27376645246207
C	7.48102343748769	-11.50503972384284	-1.61943317716741
C	7.84147260246291	-11.64795888591690	-0.27733881982628
C	8.27033046975147	-10.50917393406438	0.41481718965681
C	8.34483726290949	-9.25493635073147	-0.19925337675325
C	7.77134237965189	-12.98679371765535	0.41190568298090
C	7.41012677212134	1.82373548740561	-10.34090979021561
H	5.62021575122902	-8.14326280254022	-0.79475009029482
H	5.43731156091235	-0.91077184499344	-6.10336369016603
H	12.35614519464712	-3.43862438491310	-6.59903371100019
H	10.35906323264218	-1.86994454252063	-7.59665762774781
H	10.53269342423741	-9.13085911728215	-2.29470431990760
H	12.45734058453401	-7.64066956790212	-3.52724006476327
H	12.44710887463422	-5.39758122121584	-4.87024940139060
C	6.99865817566149	-3.01359198224481	-8.92994680739002
H	6.88653799292710	-0.84939697851693	-10.57013272505564
H	8.23460715684501	2.10887660265640	-7.75602301246001
C	8.58846025233554	0.47297320553353	-5.62063556989898
C	7.12917911189936	-10.17745121004873	-3.72368534477575
H	7.14231832977898	-12.38214214997387	-2.17762960794162
H	8.55679878687885	-10.59877961258462	1.46663145278797
C	8.81499306876591	-8.06580071360225	0.60274034418797
H	7.41542353191913	-13.76887748960839	-0.27149161568720

H	7.08920887819426	-12.95487937122778	1.27508160229375
H	8.75832495285957	-13.29184448974843	0.79167507049782
H	-0.45459658763876	-11.45343826602597	3.11454183188971
H	-0.29176233027017	-10.64536362001979	4.68975972748876
H	1.15124434240506	-11.21944802675700	3.84095970949570
H	-0.85567423149382	3.60493098510662	-7.92464856471807
H	0.74563516441215	4.27407016497104	-7.53845752820475
H	-0.68765028583983	4.87237161381200	-6.68921637665907
H	7.05756785721779	1.40475417671189	-11.29249482876372
H	8.37300566383314	2.32359499817276	-10.52640637433321
H	6.69732956505659	2.60209611562867	-10.02862362137317
H	7.22221017043458	-9.15765538194422	-4.11327733452629
H	6.08422958171039	-10.49620746972152	-3.85571535927299
H	7.75174696102364	-10.83694671840025	-4.34675834029395
H	8.82837466589027	-7.14597909392829	0.00788610489181
H	9.83100669639900	-8.23356665075381	0.99031678105501
H	8.15970747359638	-7.89754795549209	1.47046341477816
H	2.01080634745123	-5.14092093730900	3.01589562685467
H	3.02413858810345	-6.39885591381293	3.75177564785688
H	1.58159850264602	-5.81890075021568	4.59971002986463
H	-0.77692144015940	-6.76230563953669	-0.61971062115444
H	-2.02258294156789	-7.91844250975068	-0.10411675172603
H	-0.57863194352196	-8.49574387289629	-0.95189619397281
H	6.67171569049679	-3.10758810157514	-9.97330825933873
H	6.19425636449212	-3.38931897356206	-8.27980486685275
H	7.86138833012235	-3.67885407784217	-8.77567325760048
H	8.79526725851319	1.54864618937208	-5.55449749484725
H	9.50572574335921	-0.07343285561594	-5.35400213458953
H	7.83916855214373	0.21661118549391	-4.85673888114384
H	-1.69704195673574	-0.73730159894586	-6.29114481729500
H	-1.79981261456058	-0.90701849868894	-4.52025708259713
H	-0.37191741931647	-1.49431880917133	-5.37120282459745
H	2.18064426166952	3.52419269444387	-2.75300333394908
H	2.62838401021412	1.80380592907016	-2.62962791409694
H	1.20290710327243	2.39693328544526	-1.77885903948823

Table S14. Coordinates for doubly fused NiDMP dimer.

	X	Y	Z
C	-3.20445544681923	-4.53534191276554	-0.68064931209139
C	-2.21264506233234	-5.29992848765377	-0.13644860284749
C	-0.96216487166426	-4.75639966742914	-0.60312632192114
N	-1.18852491893243	-3.63640569221548	-1.38855444603415
C	-2.56221391673288	-3.48911861736732	-1.43318242941190
C	-2.27988229859853	0.86140932569975	-3.07084841368310
C	-3.25329446386757	-0.06673744721101	-2.83681647014737
C	-2.58614836275868	-1.29457819829321	-2.48816819422867
N	-1.21300967346548	-1.13342403001435	-2.55931221981728
C	-1.01427266703055	0.18777702916150	-2.91899827056830
C	-3.22929878764667	-2.42248771818291	-2.01185861754981
C	3.46647156363213	-0.52647408609206	-3.94005296160785
C	2.62927013793745	0.54013783433287	-3.77713877647775
C	1.38354071574630	0.02335251137261	-3.27757826384349
N	1.48742055960698	-1.34487605400476	-3.06240107075958
C	2.75443480332415	-1.69466529519228	-3.49131290778746
C	0.21783419280128	0.77667303758994	-3.19065069231709
C	2.62652093889722	-5.69325807568449	-1.12960064955134
C	3.45676887840263	-5.07438555243824	-2.04449809609481
C	2.72454836743117	-3.91116817613067	-2.50922104680872
N	1.49061271069858	-3.83020557051128	-1.91087969776868
C	1.41155533892188	-4.93830419206700	-1.07638144394106
C	0.27278932084808	-5.36054148749135	-0.39411711857619
C	3.30636176215952	-2.97985834592132	-3.37288995135202
Ni	0.14037443997994	-2.49039472386637	-2.23554564364031
C	0.26434495136582	2.22835529574437	-3.53893307788883
C	0.36562295770137	-6.56197274495110	0.48780620400506
C	0.16094256440471	2.61647346694514	-4.89233042347192
C	0.20404909519021	3.97780359235875	-5.20917837708513
C	0.34604639183423	4.96223852422454	-4.22388474379516
C	0.44369972216197	4.55181836057062	-2.89131666643278
C	0.40430523313585	3.19902643093713	-2.52905617238986
C	0.16271026225153	-7.85024358535529	-0.04667765114830

C	0.26960238200925	-8.95951707584742	0.80250646169401
C	0.57059411954483	-8.82520437254897	2.16016789739355
C	0.76802973156141	-7.53544956506246	2.66692708926978
C	0.67285766799578	-6.40018377761802	1.85579488280597
C	0.67933141738024	-10.03055218839168	3.05892459228160
C	0.39270332893323	6.42467139956532	-4.58742062862106
H	-4.28016854520730	-4.63945759043031	-0.56806733623673
H	-2.29752471796598	-6.17361052019343	0.50189288354362
H	-2.38639086722037	1.90711252189793	-3.34176284590415
H	-4.33209390793225	0.06105479148013	-2.86049363902469
H	-4.31857241320484	-2.42424759085304	-1.99153361111434
H	4.49892051284476	-0.51616000982419	-4.26783585114303
H	2.81854126402943	1.58822376968468	-3.98636626779849
H	2.77681773017416	-6.62058102285228	-0.58989772290461
C	0.00128427120336	1.58450298108499	-5.97880002077932
H	0.12412132323966	4.27868191894530	-6.25702624669456
H	0.55542017776417	5.30432129426872	-2.10547947161581
C	0.51663815792810	2.81521225689750	-1.07414431106694
C	-0.16001449154837	-8.06528440538264	-1.50479799936754
H	1.00405725570771	-7.40740035943609	3.72725077119668
C	0.90352073293328	-5.03627340077245	2.45965516019755
C	5.53710016678359	-6.83627600274229	-0.63504472133450
C	6.63911308593326	-7.61147982933420	-0.41142809943922
C	7.39177011095912	-7.63410764374615	-1.63654850866920
N	6.78917444856917	-6.81322080447012	-2.58123568275208
C	5.63689858899390	-6.33872218435516	-1.98231308614133
C	5.28230512553560	-2.86832103027864	-5.05205681518978
C	4.52945686436404	-3.40303531335434	-4.02398235373535
C	5.19941660319448	-4.63490781728134	-3.65119553305863
N	6.31200919504590	-4.84847246745319	-4.42864697945922
C	6.35729486876411	-3.77736742566309	-5.31175706271799
C	4.77107806030739	-5.39784047102643	-2.56198481744542
C	9.34237308994301	-5.97827388710408	-8.11791196904199
C	8.78936618203534	-4.73382297585095	-8.01842584918193
C	8.06203086143819	-4.69749021855396	-6.77445118445696

N	8.21524258773001	-5.89552421815721	-6.09416847471670
C	9.00543259124781	-6.68456593148651	-6.90887509398034
C	7.22271102447652	-3.65490529011824	-6.39661831007008
C	9.93150266734140	-9.70371336918806	-3.48779215090796
C	10.14574017679768	-9.60803287037158	-4.83273428198666
C	9.40326107546556	-8.46374865306620	-5.29482013575118
N	8.69596825056437	-7.89483538567062	-4.24971799780422
C	9.01360124134099	-8.64986773520149	-3.13434065440342
C	8.46429613146990	-8.48949662819024	-1.86514716027331
C	9.50803848327488	-7.92841474990950	-6.56599240248411
Ni	7.49928983279423	-6.36695478730232	-4.34413954745451
C	7.15999704131003	-2.42004254572207	-7.23128634461803
C	8.95508960998160	-9.35232879462562	-0.74870748256130
C	6.30229092894944	-2.36255877157559	-8.34668045913069
C	6.26102553091549	-1.18559573884224	-9.10533924945910
C	7.04217677766918	-0.07163579571861	-8.78411061578558
C	7.88279964829468	-0.15333132172593	-7.66677530068437
C	7.95432522065858	-1.30846431310614	-6.88246074163179
C	8.31629880474272	-10.58157861278202	-0.47436481392382
C	8.78993316632821	-11.36968874889545	0.57897395511488
C	9.87863556542464	-10.97619557974779	1.36637926686923
C	10.49550833369950	-9.75766648250501	1.07343271217294
C	10.05394554958742	-8.93570857634655	0.02794275229663
C	10.36718606139148	-11.84788434939721	2.49497995974137
C	6.98425136873472	1.18579126408928	-9.61333917133524
H	4.76086695201482	-6.56724775025135	0.07110037372650
H	6.92310165849600	-8.14010869973184	0.49274461841097
H	5.09741073479446	-1.97959582340739	-5.64433181513126
H	9.95282290076414	-6.38703028245196	-8.91852763531115
H	8.83311222367159	-3.90896447170896	-8.72278922794635
H	10.33192912700479	-10.42579989981370	-2.78338767540421
H	10.77460499502650	-10.22852965524431	-5.46534058813584
H	10.11043412487482	-8.46081220083607	-7.30125948254365
C	5.43579007684398	-3.53911622550405	-8.71327175637566
H	5.59514691259905	-1.13925383481600	-9.97093538948392

H	8.50122017012148	0.70667604167394	-7.39604230145681
C	8.86135098120092	-1.36581646239339	-5.68054723631923
C	7.14298005640951	-11.06694691176211	-1.29071081339244
H	8.29381412079156	-12.32158245972053	0.78917108353642
H	11.34718795598897	-9.43184770355184	1.67689664049757
C	10.75965966898377	-7.62818584452305	-0.23394612031288
H	0.11329515665696	-9.95858358198045	0.38654992402283
H	0.50667040157433	-10.96055258603317	2.50151318322068
H	-0.05563609345599	-9.98436125067815	3.87686894130637
H	1.67531938400837	-10.09330063198679	3.52298638882201
H	11.22287163779956	-11.39186155430117	3.01002668221043
H	10.68102212071172	-12.83664562677521	2.12714589737603
H	9.57478319048095	-12.01766858139730	3.23977214088947
H	6.26422502505257	1.08692283244600	-10.43618152738899
H	7.96705996610146	1.42130108563433	-10.04942897479187
H	6.68573867923915	2.05202213371699	-9.00359437113988
H	0.30444650031330	6.56781022606938	-5.67233576299427
H	1.33644988795183	6.88677719817473	-4.26020930460276
H	-0.42422369066635	6.98238950583025	-4.10479405761220
H	10.30962921378667	-7.07955747339610	-1.06887245500378
H	11.82065363414008	-7.79683999700262	-0.47223450732814
H	10.72529564718408	-6.98061891320119	0.65508970830782
H	6.88007606180670	-10.36679228835967	-2.09083891498494
H	6.25447549880268	-11.20275683173343	-0.65599746770060
H	7.36575998332010	-12.04112533330585	-1.75113482218141
H	-0.19787638362054	-7.12282626990988	-2.06164519816358
H	-1.13288154781707	-8.56651445343172	-1.61941792754953
H	0.59525730752755	-8.70913446372750	-1.97985399307651
H	0.74623899302379	-4.23204036259481	1.73208737806110
H	1.93122642083081	-4.94753244093246	2.84332449957439
H	0.22416440453026	-4.86526711497404	3.30781073680163
H	-0.07322108571050	2.06204332117429	-6.96407310165747
H	-0.90121851143529	0.97553275565763	-5.81960244568541
H	0.85150146151165	0.88633404866686	-5.99494387624108
H	0.47827159351092	1.72906189561906	-0.93342906879398

H	-0.29998115331884	3.26180846337718	-0.48725905548648
H	1.46229414538970	3.17951614429292	-0.64538427068248
H	4.83129137829711	-3.31862625249846	-9.60218047186565
H	4.75391408700958	-3.80334271868185	-7.89079471021415
H	6.04150937661531	-4.43412621912673	-8.91882585905405
H	9.43596424227358	-0.43643906008876	-5.57762174077967
H	9.56900333385460	-2.20511851678606	-5.75222545546932
H	8.28679258231914	-1.52019364964587	-4.75471566818867

Table S15. Coordinates for triply fused NiDCOOMePP dimer.

	X	Y	Z
C	-1.67888445848896	-3.32812553374855	2.60569676497211
C	-1.09686266785950	-4.55934949614466	2.52871542638609
C	-0.18388914480262	-4.52304139254501	1.41248967075886
N	-0.22694333468474	-3.28387136842686	0.79530313831690
C	-1.15789510001967	-2.55183304208259	1.51354099310090
C	-1.47627161118895	0.74465369766982	-1.76444810717633
C	-1.99001286495007	0.48926618397539	-0.52665459456565
C	-1.29463272588759	-0.66017309256663	-0.01553340472364
N	-0.33223926282952	-1.08766780907088	-0.91376861817084
C	-0.41739512848756	-0.20977259713110	-1.98405470697475
C	-1.63153296626977	-1.30247956421463	1.16131904530630
C	3.61634937984843	-1.75617360643670	-3.74973703837379
C	2.64126173649424	-0.84259748118466	-4.08262532936591
C	1.57373544669083	-1.00519206183776	-3.13268597912412
N	1.87111719183899	-2.02003339476375	-2.22878618360473
C	3.10569226728130	-2.48567768838689	-2.61325412045493
C	0.45310415841335	-0.17849633148335	-3.07302923307482
C	2.80041210648958	-6.32697235641363	-0.02212799345095
C	3.71812619032472	-5.71857010865959	-0.84879102011058
C	3.16705072622412	-4.42328458185116	-1.16982911156804
N	1.96821278891849	-4.21378428896477	-0.53270524541575
C	1.74464013352317	-5.37658513117525	0.20136729204095
C	0.69680614440723	-5.55944212015271	1.10094638873417
C	3.77663704012683	-3.59529373930397	-2.10822240461538

Ni	0.81942278830611	-2.65061695831533	-0.71837507653376
C	0.24452655447920	0.81020116749208	-4.16203049694400
C	0.56066194811829	-6.85203572974013	1.82043123291797
C	-0.03227460807387	0.36865781989473	-5.46722039627268
C	-0.21430837198263	1.28155821507168	-6.50090917049576
C	-0.11884791329623	2.65863902357536	-6.25312607111908
C	0.16406504070367	3.10732430123751	-4.95406422376783
C	0.34363383079663	2.19050808937628	-3.92151548936245
C	-0.58301065880307	-7.64637464030241	1.63238909205489
C	-0.72242190382008	-8.85795404187953	2.30173223480817
C	0.27829649895697	-9.30076379330479	3.17879535352146
C	1.41910026311310	-8.50881990018993	3.37933350096492
C	1.55576322721811	-7.29742542909741	2.70664396164073
C	0.07757605556478	-10.60701333551363	3.86444803742804
C	-0.32150043078755	3.58917233560528	-7.39730372710679
O	1.12309309065105	-10.92475420138330	4.67746603069840
O	-0.21620782250066	4.89434479591120	-7.02137948525699
H	-2.41486484358301	-2.96950523856414	3.31987964573095
H	-1.23864580019802	-5.41583211231386	3.17904235469221
H	-1.77015290097408	1.51370077597335	-2.47027332378778
H	-2.79529693188983	1.00203549122674	-0.00810681309952
H	-2.38865266301566	-0.84377156911344	1.79633070718899
H	2.64955205652924	-0.10702291190370	-4.87986509456653
H	2.83870904878191	-7.32260580763139	0.40579152840748
H	-0.11045081978293	-0.70196882012609	-5.65939009801007
H	-0.43635464026356	0.94850147209041	-7.51475832744231
H	0.25075234455763	4.17543509005576	-4.76122646536616
H	0.58053301845007	2.53962604598201	-2.91595987635562
H	-1.35832556279040	-7.30597701328764	0.94510026165170
H	-1.60127369512511	-9.48585906706979	2.15520486204304
H	2.19190212765873	-8.84434323387596	4.06907945196534
H	2.43489846701489	-6.67426770870359	2.87340027062243
O	-0.90184795209923	-11.31927707526489	3.72845688484278
C	0.98688612610480	-12.18464599796825	5.36736339826223
H	1.89417264687507	-12.28519069987935	5.97118997875815

H	0.90507663867044	-13.00913983847593	4.64702126145757
H	0.09312204240653	-12.17899191367597	6.00494073159691
O	-0.55502713230682	3.24218335512388	-8.54194807856889
C	-0.40571352296143	5.84507628430732	-8.08967970837143
H	-0.29942060942795	6.83027168866103	-7.62490352602327
H	-1.40287455065427	5.72926939724394	-8.53418854676767
H	0.35084575907044	5.70018853867894	-8.87213490136790
C	4.97453362760739	-6.10515432056648	-1.42672773221461
C	5.75763098694165	-7.23672709884216	-1.38455642674460
C	6.91390593988724	-6.97565381670670	-2.19880989232287
N	6.84402386984789	-5.70369155127271	-2.75861749915493
C	5.65964770877528	-5.17999341957372	-2.29811196261488
C	5.67128476206683	-1.66862965448011	-5.33665177392300
C	4.90152197493738	-2.11007418777655	-4.28411849787387
C	5.63390137991062	-3.20197095841923	-3.68647943129257
N	6.83553116005000	-3.40944055169836	-4.31930263112453
C	6.87892139500346	-2.44907296436307	-5.32748397041462
C	5.07164776327381	-3.97677951106616	-2.67606794601836
C	11.39975345486290	-3.27658865949156	-6.08769425197729
C	10.41408744235697	-2.49565770416414	-6.61598062354477
C	9.18160267310629	-2.88617640999119	-5.97640276586717
N	9.40862272028616	-3.91901009974668	-5.08153846473802
C	10.76784353795742	-4.17327540084375	-5.15844386842035
C	7.97233511662472	-2.21548057558827	-6.15772500797132
C	10.41094397307695	-8.22024745790889	-2.87567838582018
C	11.39186714052354	-7.43839612068473	-3.41150592330662
C	10.76828628821255	-6.21037065917404	-3.82288235081988
N	9.42044051187150	-6.21735267296036	-3.50791869311726
C	9.19606090655514	-7.44335952756215	-2.89906034537991
C	7.99877376271360	-7.84362268537793	-2.30759716820195
C	11.41882721683004	-5.22035533401901	-4.53501301840204
Ni	8.12899285048901	-4.81252906788649	-3.91649980523734
C	7.88767288635607	-1.16448961972332	-7.20492407475313
C	7.88967500256829	-9.19283156836372	-1.69475340099777
C	7.96812651461498	-1.51421847475243	-8.56306378107930

C	7.88985326737540	-0.53824281202606	-9.55152385097103
C	7.73470235558091	0.81112767052538	-9.20263756672614
C	7.66302093501630	1.16921124228453	-7.84792746520299
C	7.73925590008115	0.18935695499314	-6.86145848376764
C	6.97623839103750	-10.12862266509930	-2.20890736897488
C	6.85227636390880	-11.38870742085586	-1.63251424692787
C	7.63720657590591	-11.73999424144818	-0.52468217480267
C	8.54732911762846	-10.80889812815838	-0.00168074664798
C	8.66982005971368	-9.54932800681521	-0.58249597735957
C	7.46021064401400	-13.10061670908695	0.05298014145487
C	7.65265412733977	1.81124565835231	-10.30267557288117
O	8.28559868182096	-13.31808933323609	1.11459994977686
O	7.48829549298086	3.07841803209640	-9.83181861836439
H	5.58617681224300	-8.15479670036349	-0.83282275511048
H	5.44910937666328	-0.88126785661470	-6.04851830973914
H	12.46254519009389	-3.27287985858954	-6.31295496714136
H	10.50092376830712	-1.70232171633669	-7.35069740149042
H	10.49087447306769	-9.22903776380075	-2.48587819049938
H	12.44261484181508	-7.67308248020972	-3.55695079051033
H	12.49121887473386	-5.32547795607544	-4.69566820943957
H	8.08176848675460	-2.56416664983860	-8.83537294286656
H	7.94081965684399	-0.80051783616132	-10.60829101373478
H	7.55414962626984	2.21733105283121	-7.57376057297058
H	7.69728562730849	0.46881015936670	-5.80817665477233
H	6.36711761534809	-9.85688618065216	-3.07174960200123
H	6.15123916131765	-12.12341460360355	-2.02861353535827
H	9.14985343864781	-11.07444074723542	0.86554356412431
H	9.36435320993806	-8.81980041371343	-0.16456072552344
O	6.67402523634729	-13.93440306476036	-0.36154393582608
C	8.15828121321993	-14.62266964780912	1.71758627719628
H	8.87708495433018	-14.63324700674734	2.54286381728928
H	8.39267840478742	-15.40774699607261	0.98681116357803
H	7.13637453426244	-14.77609256715759	2.08845940279540
O	7.71947702904453	1.54436025435884	-11.48989068171055
C	7.39164716843575	4.09208538869432	-10.85413465145324

H	7.27109069518785	5.03742616983451	-10.31593700826535
H	6.52702384011334	3.90032359661731	-11.50297081355557
H	8.30141000040191	4.10637331049167	-11.46846730179987

Table S16. Coordinates for doubly fused NiDCOOMePP dimer.

	X	Y	Z
C	-3.24797829359642	-4.55871887570245	-0.73727032918828
C	-2.26457192069462	-5.29224553429758	-0.13907171521767
C	-1.00608046220835	-4.79100907447097	-0.63589493829950
N	-1.22327721706482	-3.70730423119541	-1.47361408112019
C	-2.59551102503041	-3.55964668154267	-1.54068504049023
C	-2.32305120396583	0.74781727172542	-3.27897538113475
C	-3.28800517402613	-0.19557426324316	-3.07600933117134
C	-2.61527864165179	-1.40061061604740	-2.66858387148651
N	-1.24341125477708	-1.21288106816391	-2.67830490904247
C	-1.05168841339475	0.10618374562831	-3.04351809456228
C	-3.25670379692459	-2.52362234826011	-2.17930160942894
C	3.46641124850863	-0.58774099345811	-3.93567929701909
C	2.63229245950610	0.48215303585101	-3.77944174959929
C	1.36160382250884	-0.03547494416493	-3.34340082275313
N	1.45449097880053	-1.40679060026355	-3.14136489854809
C	2.73195360645098	-1.75707640770585	-3.53375540884180
C	0.18635518519996	0.71057814327610	-3.27299677785962
C	2.58316710175706	-5.74806358574348	-1.16316544493685
C	3.41383476501477	-5.13731230808191	-2.08285817252576
C	2.68782251221514	-3.97341455491125	-2.54993914209007
N	1.45831108729032	-3.88041155276149	-1.94370258739416
C	1.37681208995830	-4.97734288499499	-1.09757471119693
C	0.23444024819040	-5.38208634640785	-0.40112086728193
C	3.27554156544504	-3.04606302137787	-3.41326108051307
Ni	0.10723236069139	-2.55642526956541	-2.31388679851385
C	0.21845843615232	2.16768958113015	-3.56030337679792
C	0.35159414316950	-6.54268548167570	0.51699564085230
C	0.61853460995411	2.65434105956510	-4.81683338394889
C	0.63222598758635	4.02080874583268	-5.07582465721323

C	0.24484499753374	4.93355192687781	-4.08373760979299
C	-0.16271621247289	4.45577066981136	-2.82904800185749
C	-0.17732733587441	3.08687583979501	-2.57432591551303
C	-0.31665467698225	-7.75158438672771	0.25783149736631
C	-0.16941060715764	-8.83921009746860	1.11244582086522
C	0.65168268749100	-8.74474049897205	2.24556773714549
C	1.32574893126461	-7.54292184553301	2.50958945718900
C	1.17658317932395	-6.45663424512548	1.65159267175579
C	0.77422038711472	-9.94319730895515	3.12052195775291
C	0.28274244959075	6.38378212146489	-4.41737767686344
O	1.62462353228676	-9.73134489383451	4.16248254306871
O	-0.12876227739897	7.16021657733974	-3.37686251768519
H	-4.32438328318448	-4.64858947323598	-0.62056973655285
H	-2.37075865318585	-6.11073957873241	0.56445819217252
H	-2.44401210644125	1.78015898578406	-3.58965275663417
H	-4.36589719454980	-0.09114074035298	-3.16305285200389
H	-4.34597497135937	-2.53886927282446	-2.19006602803734
H	4.51172501554547	-0.57794967088560	-4.22062946082105
H	2.85272052523131	1.53109511480167	-3.94452397766862
H	2.73415723282949	-6.68201301322360	-0.63584169927139
H	0.90298039083211	1.94579603690939	-5.59520725550128
H	0.93347071110820	4.40696396173358	-6.04964126491360
H	-0.46217544023408	5.16183183461452	-2.05618617118029
H	-0.48872538005566	2.71499401749641	-1.59750961630356
H	-0.93509955131955	-7.83740752931248	-0.63602736555142
H	-0.67536149910776	-9.78413206613109	0.91403068911937
H	1.96220068443321	-7.46527722111035	3.38961994873835
H	1.69697182220776	-5.52044988513162	1.85725368632730
O	0.19125449127290	-10.99785360533286	2.93831133242853
C	1.78710445584538	-10.86039425100478	5.04654332856480
H	2.49729223300595	-10.53124477514830	5.81138318215185
H	2.18016970376967	-11.72494375571752	4.49597337568841
H	0.82528694168175	-11.13413532353074	5.49966721620136
O	0.63792761152568	6.84070167794934	-5.49013972872599
C	-0.11359834550746	8.57837179296029	-3.64149074361455

H	-0.46985330356176	9.05014541480155	-2.72042751855704
H	-0.77552080183937	8.81890314189785	-4.48371002973116
H	0.90392536619314	8.91335523055886	-3.88208826409442
C	5.50087361297750	-6.86239773500326	-0.64199818031101
C	6.60417687466725	-7.63201647980548	-0.40581301450051
C	7.33767598756943	-7.70682778217432	-1.64239196673215
N	6.73070600401568	-6.90396579469575	-2.59982051207840
C	5.58819146013871	-6.40673727527608	-2.00336431549405
C	5.27559418523791	-2.92405438609649	-5.05941168950313
C	4.50528678957123	-3.46859854705829	-4.04983116833182
C	5.16843596568326	-4.70136238674130	-3.67606599586363
N	6.29672161335418	-4.90532252888812	-4.43317805976710
C	6.36489318705077	-3.82379982561096	-5.29976681228396
C	4.72732910437575	-5.46796283174851	-2.59476061834135
C	9.33535090913486	-6.05732884748912	-8.10663015366898
C	8.83765866758841	-4.79171914596712	-7.98865028082002
C	8.06839237357997	-4.75529865788838	-6.76856390846307
N	8.16312733943476	-5.97235974547236	-6.11092877771352
C	8.93910343821070	-6.77541705391847	-6.92458469756945
C	7.25698580866039	-3.69443453103254	-6.36851848212817
C	9.80561596424276	-9.83709086049488	-3.53075898497029
C	9.98953345689308	-9.75444104007439	-4.88066240293176
C	9.28199518669577	-8.58387844512843	-5.32820562362928
N	8.61994921615237	-7.98827221266598	-4.26804704633735
C	8.94002317315503	-8.74419604643011	-3.15659951171967
C	8.41240451838548	-8.56377096083621	-1.87559163310206
C	9.39225785317697	-8.04272780106394	-6.59624085869888
Ni	7.44861122525129	-6.44613026105786	-4.35938686693677
C	7.24101462443741	-2.42588494013813	-7.13895910062919
C	8.95192496603722	-9.39181835694491	-0.76697291753036
C	6.83882168788774	-2.38689906000954	-8.48504421525569
C	6.80106621339566	-1.18143439943433	-9.17834911865829
C	7.16258036914177	0.01458880381971	-8.54129703443525
C	7.56174348301998	-0.01478086081426	-7.19654888036172
C	7.59725528740075	-1.22275569122808	-6.50557117582771

C	8.15604258761337	-10.34626032815297	-0.11029237358769
C	8.68033689135279	-11.11689920036154	0.92222117881333
C	10.01463716053701	-10.95218632512534	1.32206117372772
C	10.81858777642425	-10.00802951200680	0.66558342053105
C	10.29082046171125	-9.24011648380830	-0.36900997218971
C	10.51883569886637	-11.80028759997949	2.43663424593817
C	7.09738085486931	1.27415769826288	-9.33251077354907
O	11.82768964542778	-11.55106206934130	2.71879367599964
O	7.44602182698404	2.36235944219438	-8.59224107163756
H	4.73753940359631	-6.56320892474452	0.06646897925630
H	6.89903086166921	-8.10940478650247	0.52223426024781
H	5.08336034565846	-2.03695727225629	-5.65051395606227
H	9.95465653759705	-6.46878514606356	-8.89880874551982
H	8.96091749710039	-3.95501936653018	-8.66749677803675
H	10.18930146931841	-10.58263421757940	-2.84236771487423
H	10.57459824363440	-10.40485304488580	-5.52488331210658
H	9.97211803776665	-8.58892786471355	-7.33943383002208
H	6.53180645149829	-3.31051511923556	-8.97646459074708
H	6.48036032578460	-1.14031908740118	-10.21934551883974
H	7.84608349176905	0.91138130414076	-6.69958725925451
H	7.91080502170645	-1.24761610203764	-5.46125927138405
H	7.12378588220169	-10.48980699231992	-0.43079969485757
H	8.07212397189165	-11.86409414329551	1.43207423240781
H	11.85445292484053	-9.87630519366434	0.97407989185426
H	10.91314004451723	-8.50119882025884	-0.87517656442631
O	9.85236921487980	-12.62420694483385	3.03893942150843
C	12.37236530955286	-12.34237147251005	3.79521913757428
H	13.41419579902677	-12.02189052944474	3.89379647421974
H	12.31497350598078	-13.41160651592510	3.55270358351852
H	11.81849007559788	-12.15856578194815	4.72516227294201
O	6.77144804067846	1.34100155188292	-10.50498004777991
C	7.40009307081708	3.61585868641677	-9.30587283795169
H	7.70192587715362	4.37588742825821	-8.57855865870109
H	6.38424504847232	3.81068495870612	-9.67369402049171
H	8.09047358205939	3.59696161695593	-10.15926456026323

Table S17. Coordinates for triply fused NiDCNPP dimer.

	X	Y	Z
C	-1.62887283754604	-3.29429207622033	2.64954145091094
C	-1.04702038365868	-4.52562647567287	2.57696877348333
C	-0.14106604435711	-4.49641770792752	1.45432556763214
N	-0.18889314068640	-3.26189333481981	0.82898016008303
C	-1.11531596874416	-2.52541390594223	1.54860002730637
C	-1.45753970116179	0.74789419642909	-1.74864549436812
C	-1.95899666020192	0.50357255069729	-0.50391834195733
C	-1.26120042319901	-0.64379185434629	0.00892132286440
N	-0.30835377034027	-1.08013903303429	-0.89515213959008
C	-0.40231545302268	-0.21034686597093	-1.97092499076115
C	-1.59013280855327	-1.27772982362827	1.19250595649891
C	3.61943687225117	-1.76653115947200	-3.75585504786286
C	2.63837810600959	-0.86027139758725	-4.09166333794898
C	1.57844890596453	-1.01666565712315	-3.13206873631963
N	1.88528245892864	-2.02147744260467	-2.22073368918190
C	3.11868161978043	-2.48680090906111	-2.60888109289533
C	0.45739786928725	-0.19091419792905	-3.06836375792771
C	2.84065586179885	-6.30634515016390	0.01747436024304
C	3.75171319038897	-5.70278956006735	-0.82031844274055
C	3.19524045050058	-4.41139745394280	-1.14843549139238
N	1.99969022785175	-4.19996453674757	-0.50611849726910
C	1.78361914563600	-5.35661525435713	0.23896299497477
C	0.73991594009208	-5.53310887326636	1.14455600810955
C	3.79642905996578	-3.58924935372456	-2.09730116932870
Ni	0.84504870323698	-2.64066207884092	-0.69776811159642
C	0.23568164324606	0.78734694158505	-4.16425817682956
C	0.60700581023612	-6.81972922205132	1.87503080763728
C	-0.06854767349418	0.33323309404553	-5.45826540755062
C	-0.27102098640839	1.23126365548117	-6.50113538418685
C	-0.16569632477578	2.61391693515585	-6.26447440286659
C	0.14757251472585	3.07810566218104	-4.97373603242692
C	0.34507776060281	2.16949659062921	-3.93925594981217
C	-0.53683223259890	-7.61481093037880	1.69413117899233

C	-0.68613328711813	-8.81729302960346	2.37702403404928
C	0.31608138184727	-9.24680957005600	3.26599062822765
C	1.46291250702450	-8.45482825159818	3.45832370523021
C	1.60071604409071	-7.25534461609002	2.76758257790425
C	0.16572553219162	-10.47817461777806	3.97433681234703
C	-0.37437308998902	3.54370096417274	-7.32875242004984
N	0.03878090239866	-11.48235164839197	4.55257465242169
N	-0.54603240685677	4.30499756531234	-8.19468762173534
H	-2.35922915138632	-2.93085821246382	3.36692430176931
H	-1.18394616475609	-5.37568205918414	3.23668629315353
H	-1.75870371013919	1.51244004126147	-2.45632542899429
H	-2.75745026884708	1.02263764068550	0.01874275934735
H	-2.34134830506304	-0.81381443926985	1.83060012915554
H	2.64010902919277	-0.13208552266944	-4.89580095658460
H	2.88507877321671	-7.29850290721180	0.45306319029626
H	-0.15418249756765	-0.73873131976182	-5.63812829274981
H	-0.51597949267100	0.87418823069482	-7.50097069479707
H	0.24060484741082	4.14912390326175	-4.79622755326304
H	0.60238474866359	2.52878230493304	-2.94263574217930
H	-1.31070554970885	-7.28219322414177	1.00184687992834
H	-1.57184569554415	-9.43402745186608	2.22766165522085
H	2.23185475907837	-8.78390316883071	4.15667246737319
H	2.48067695918502	-6.63240485765413	2.92897404332871
C	5.00534184617160	-6.09085470906586	-1.40367711331546
C	5.79010058069115	-7.22152550257153	-1.36217162841339
C	6.94132255519936	-6.96188474915059	-2.18432382854272
N	6.86660063433802	-5.69238709481925	-2.74779436859548
C	5.68377515650435	-5.16868040692126	-2.28363215272932
C	5.66932133470682	-1.68165357320049	-5.35059139321220
C	4.90394477374342	-2.11911254625228	-4.29288385244755
C	5.64368505221005	-3.20219573288506	-3.68821222285974
N	6.84467604513663	-3.40819452668948	-4.32232605921821
C	6.88151425518201	-2.45526624430043	-5.33721586396979
C	5.08978790032211	-3.97063391132547	-2.66836597391623
C	11.41224840838534	-3.25182894681723	-6.07767305129411

C	10.42258967227279	-2.48176438872046	-6.61388392032276
C	9.19016901470987	-2.87993701362046	-5.97807611322884
N	9.42183040158676	-3.90657534758682	-5.07782781588905
C	10.78340437659173	-4.15022530439188	-5.14790577560370
C	7.97547021875835	-2.22103653010102	-6.16670432092871
C	10.44122434741274	-8.19880037093272	-2.86839709162341
C	11.41996572352584	-7.41182539055183	-3.40026121352795
C	10.79196169586994	-6.18574639022622	-3.81090897534924
N	9.44357929429884	-6.19899330563625	-3.49886784811791
C	9.22327748697794	-7.42608974479980	-2.89144104368132
C	8.02737675373825	-7.82798524660997	-2.29834545684887
C	11.43933114430930	-5.19183736998467	-4.52053956233751
Ni	8.14581313747309	-4.80241415583129	-3.91247424832891
C	7.88850902520330	-1.17772572255258	-7.22114768497102
C	7.92195500915602	-9.17929102736869	-1.69012004895427
C	7.97898184153714	-1.53749428447414	-8.57562825109881
C	7.91065461897769	-0.57342926944503	-9.57615364282881
C	7.75421578579660	0.78187969661846	-9.23316314800249
C	7.66802441692475	1.15257223750473	-7.87871775316478
C	7.73503608428748	0.17824574984346	-6.88816294534742
C	7.01157445189242	-10.11439128975075	-2.20971619244205
C	6.89099312478563	-11.38161094718603	-1.64884557199068
C	7.68490825436909	-11.73614199101436	-0.54297610017260
C	8.59312088901302	-10.80326717886891	-0.00974455330915
C	8.70538570567073	-9.54027501610376	-0.58151970446241
C	7.56885315358743	-13.03629882353641	0.03741150354728
C	7.68922218363268	1.77744729112413	-10.25562969951150
N	7.47583668307307	-14.09751213166761	0.51072272503139
H	5.62271285390789	-8.13687456757000	-0.80455929731079
H	5.44025664313694	-0.90062388633748	-6.06737740591348
H	12.47599761650592	-3.23910590126415	-6.29762547906743
H	10.50909012376088	-1.68994074020569	-7.35026117721541
H	10.52705311860585	-9.20846062523088	-2.48210394260995
H	12.47190698778227	-7.64220373011758	-3.54329895400262
H	12.51296670068669	-5.28989201619060	-4.67679145254685

H	8.09652047672005	-2.58883063719985	-8.83977524793538
H	7.97361991564807	-0.85814160566509	-10.62599221113393
H	7.55807261972774	2.20420400852788	-7.61558403948545
H	7.68473056838028	0.46641954332413	-5.83789713563660
H	6.39981134110411	-9.83909225292970	-3.06925161692137
H	6.18976388049960	-12.10626535808056	-2.06157192149157
H	9.19785626008942	-11.07595521396313	0.85466787467047
H	9.39770158259394	-8.81217903341173	-0.15821273042615
N	7.63854867641862	2.58903210111104	-11.09084363431635

Table S18. Coordinates for doubly fused NiDCNPP dimer.

	X	Y	Z
C	-3.28236721218646	-4.54948013994510	-0.82232277418489
C	-2.30918528055512	-5.29420167947431	-0.22155393988517
C	-1.04288602469832	-4.79637896366757	-0.70244996880589
N	-1.24549763473834	-3.70502632202775	-1.53316699782538
C	-2.61606193102192	-3.54774342327561	-1.61111316105545
C	-2.29762101477631	0.77445082944716	-3.30231888232463
C	-3.27127700226541	-0.16314481312285	-3.11644907181440
C	-2.61084534641932	-1.37801958640800	-2.71780217339468
N	-1.23749373264273	-1.20136283790915	-2.71620366489225
C	-1.03277519296647	0.11961097226593	-3.06597370664975
C	-3.26462233351269	-2.50100958538793	-2.24523635415689
C	3.48307156134685	-0.60706809947989	-3.94672332386202
C	2.65839903285408	0.46926536681524	-3.78464515327324
C	1.38150844311453	-0.04036781296911	-3.35674155810891
N	1.46121350294176	-1.41372780721506	-3.16531058664275
C	2.73689408039376	-1.77261315738525	-3.55549840669958
C	0.21205337071833	0.71448185766204	-3.28319402743921
C	2.54833891001931	-5.76977650651657	-1.19898949153470
C	3.38799101808218	-5.16175856256532	-2.11243725765779
C	2.67184104045201	-3.99218675154179	-2.58043390025653
N	1.43948954256437	-3.89329701922806	-1.98146310843164
C	1.34530285758664	-4.99269836294004	-1.14033053708574
C	0.19252552263630	-5.39442765902472	-0.45967751105040

C	3.26955378222851	-3.06635955025168	-3.43854557078182
Ni	0.10017246468042	-2.55790937296983	-2.35405182424909
C	0.25725450148128	2.17428950587502	-3.55420685909548
C	0.29191395932148	-6.56326230182990	0.44999834587858
C	0.66351483621596	2.67113884599365	-4.80408851210355
C	0.68819300576256	4.03898257668903	-5.05474598194024
C	0.30172772486961	4.94291918638064	-4.04822371346439
C	-0.11188140992449	4.45532334423059	-2.79508490325777
C	-0.13426849933466	3.08492185584310	-2.55846269471311
C	-0.37017254612352	-7.76891755889246	0.16405675501721
C	-0.24484193751853	-8.86871547397135	1.00624397609333
C	0.55386106956466	-8.78073271160522	2.16114009846244
C	1.22300352666721	-7.57856109997506	2.45533419918062
C	1.09140876137006	-6.48728292866767	1.60336477194473
C	0.69189488743882	-9.90842221234956	3.02692017139119
C	0.32460959855401	6.34893476658314	-4.29974550168592
H	-4.36022355981720	-4.63313537783162	-0.71551268396508
H	-2.42896348372576	-6.11622196839850	0.47572884208179
H	-2.41026730796901	1.81074288624665	-3.60281453019374
H	-4.34754224979085	-0.04864588027220	-3.20974209457658
H	-4.35378405260770	-2.50811915366821	-2.26511558219061
H	4.52997263828722	-0.60422780764227	-4.22600815785074
H	2.89155179436978	1.51724909862868	-3.93823018493093
H	2.69217965460356	-6.70618906452189	-0.67378449977146
H	0.94554258131911	1.97006590044511	-5.58972268084273
H	0.99633815438919	4.41849072513973	-6.02852461701246
H	-0.40799044085609	5.15751795098183	-2.01636385594007
H	-0.45033725416015	2.70572834703462	-1.58630083109605
H	-0.97064154391163	-7.84532870688658	-0.74253438192174
H	-0.75130121194734	-9.80505566423399	0.77380370450021
H	1.83756731652951	-7.51098870914331	3.35254259407064
H	1.60619987262358	-5.55344947227702	1.83104633807565
N	0.80839890896760	-10.82852638519806	3.73318189131688
N	0.34345553452086	7.49582286134665	-4.50795359705782
C	5.46413661335981	-6.89790343508959	-0.66589643453315

C	6.56526108830635	-7.66964532291097	-0.42636225265843
C	7.29980370185554	-7.74930653089238	-1.66219288714634
N	6.69589952296578	-6.94782899394775	-2.62215919247330
C	5.55472162465689	-6.44489653078554	-2.02796775323641
C	5.27146413189256	-2.95905979913637	-5.08330047398402
C	4.49700443697099	-3.49745930265322	-4.07355271424764
C	5.14914424409267	-4.73629717422988	-3.70087376807237
N	6.27485299049200	-4.94991656993541	-4.45883527086328
C	6.35125037411227	-3.86993389481727	-5.32622914123586
C	4.70089980623370	-5.50061954742711	-2.62071734359374
C	9.28958332537932	-6.14104602400672	-8.14043688489696
C	8.80110261563950	-4.87167873308441	-8.02618583776033
C	8.03860685331239	-4.82395240339268	-6.80193113949572
N	8.12787711415014	-6.03820145916148	-6.13922636190499
C	8.89452076941634	-6.85042233995918	-6.95264094873763
C	7.23891897172527	-3.75508315401181	-6.40023155300737
C	9.76289330752058	-9.89302995841324	-3.54368219281113
C	9.94198145122238	-9.81988118529389	-4.89451279513712
C	9.23538750645698	-8.65058087401100	-5.34717296256846
N	8.57824716690221	-8.04651830424551	-4.28879406051456
C	8.90003819896476	-8.79603187015214	-3.17382128799798
C	8.37411874644608	-8.60729753777992	-1.89333150566825
C	9.34268758250918	-8.11812368455333	-6.61907277502406
Ni	7.41549945570026	-6.49957777894611	-4.38378442846414
C	7.22836583926322	-2.49171591150598	-7.17918144100310
C	8.91976812870371	-9.42714765154893	-0.78169325501017
C	6.80552373431317	-2.45947597615114	-8.51856720501877
C	6.77001163685778	-1.26304680886194	-9.22713352338873
C	7.16022754369235	-0.06499264163911	-8.60122038806765
C	7.58297706337429	-0.08603478949457	-7.25943324185808
C	7.61140329238251	-1.28858208997327	-6.56173589571664
C	8.13164507403718	-10.38251097729600	-0.11824165940554
C	8.65983339932989	-11.15190353852256	0.91334328391136
C	10.00055980777928	-10.97717020583746	1.30232132142139
C	10.79976266703538	-10.02739376984470	0.64006824260619

C	10.25998238534279	-9.26664653387068	-0.39125604107753
C	7.11965012450368	1.16765962113510	-9.32174442468587
H	4.70210344992937	-6.59331525850297	0.04168475606997
H	6.85792886902908	-8.14284566190647	0.50457995209047
H	5.08462213461070	-2.06949145878394	-5.67259998685103
H	9.90264140696429	-6.56019357262660	-8.93333793693475
H	8.92981042834544	-4.03975675033000	-8.70993862929442
H	10.14793453748347	-10.63609713627197	-2.85336651620938
H	10.52325912250520	-10.47605794202350	-5.53617323000560
H	9.91683124535154	-8.67150375231005	-7.36128854292743
H	6.48013716215798	-3.38315938577635	-8.99731922521268
H	6.43042633797574	-1.24230247194704	-10.26226848813458
H	7.88930874226066	0.84216344413902	-6.77787122259297
H	7.94177979151170	-1.30768758836058	-5.52281368195441
H	7.09839841416988	-10.53211738823134	-0.43189169651563
H	8.04612672516687	-11.89713477227587	1.41854228744073
H	11.83713011273188	-9.89217219920153	0.94465152629967
H	10.87676200598291	-8.52664892863282	-0.90195063248571
N	7.08331479653743	2.17400547071579	-9.90907148104341
C	10.55167558707096	-11.76306125541108	2.36030819498600
N	11.00712158030991	-12.40214609695198	3.22226787426214

References:

[1] Osuka, A.; Shimidzu, H. meso, meso-Linked Porphyrin Arrays, *Angew. Chemie Int. Ed. English* **1997**, *36*, 135–137.

[2] Tsuda, A.; Nakamura, Y.; Osuka, A. Synthesis of meso-β doubly linked porphyrin tapes, *Chem. Commun.* **2003**, 1096–1097.

[3] Bansal, D.; Ghahramanzadehasl, H.; Cardenas-Morcoso, D.; Desport, J.; Frache, G.; Bengasi, G.; Boscher, N. D. Directly-Fused Ni(II)Porphyrin Conjugated Polymers with Blocked meso-Positions: Impact on Electrocatalytic Properties. *Chem. Eur. J.* **2024**, 30, e202400665.

[4] Tsuda, A.; Osuka, A. Fully Conjugated Porphyrin Tapes with Electronic Absorption Bands That Reach into Infrared. *Science*, **2001**, 293, 79–82.

[5] Feng, C.-M.; Zhu, Y.-Z.; Zhang, S.-C.; Zang, Y.; Zheng, J.-Y. Synthesis of directly fused porphyrin dimers through Fe(OTf)3-mediated oxidative coupling. *Org. Biomol. Chem.*, **2015**, *13*, 2566–2569.

[6] Brennan, B. J.; Arero, J.; Liddell, P. A.; Moore, T. A.; Moore, A. L.; Gust, D. Selective oxidative synthesis of meso-beta fused porphyrin dimers. *J. Porphyrins Phthalocyanines*, **2013**, *17*, 247–251.

[7] Brennan, B. J.; Kenney, M. J.; Liddell, P. A.; Cherry, B. R.; Li, J.; Moore, A. L.; Moore, T. A.; Gust, D. Oxidative coupling of porphyrins using copper(ii) salts. *Chem. Commun.*, **2011**, *47*, 10034–10036.

[8] Nakamura, Y.; Aratani, N.; Tsuda, A.; Osuka, A.; Furukawa, K.; Kato, T. Oxidative direct coupling of metalloporphyrins. *J. Porphyrins Phthalocyanines*, **2003**, *7*, 264–269.

[9] Tsuda, A.; Nakano, A.; Furuta, H.; Yamochi, H.; Osuka, A. Doubly meso-β-Linked Diporphyrins from Oxidation of 5,10,15-Triaryl-Substituted Ni^{II}– and Pd^{II} – Porphyrins. *Angew. Chemie Int. Ed.*, **2000**, *39*, 558–561.

[10] Aratani, N.; Osuka, A. Directly linked porphyrin arrays, *Chem. Rec.*, **2003**, *3*, 225–234.

[11] Lock, J. P.; Im, S. G.; Gleason, K. K. Oxidative chemical vapor deposition of electrically conducting poly (3, 4-ethylenedioxythiophene) films. *Macromolecules*, **2006**, 39, 5326.

[12] Kovacik, P.; Del Hierro, G.; Livernois, W.; Gleason, K. K. Scale-up of oCVD: large-area conductive polymer thin films for next-generation electronics, *Mater. Horiz.* **2015**, 2, 221.

[13] Goktas, H.; Wang, X.; Boscher, N. D.; Torosian, S.; Gleason, K. K. Functionalizable and electrically conductive thin films formed by oxidative chemical vapor deposition (oCVD) from mixtures of 3-thiopheneethanol (3TE) and ethylene dioxythiophene (EDOT). *J. Mater. Chem. C*, **2016**, 4, 3403.

[14] Ikeda, T.; Aratani, N.; Osuka, A. Synthesis of Extremely π-Extended Porphyrin Tapes from Hybrid meso-meso Linked Porphyrin Arrays: An Approach Towards the Conjugation Length. *Chem. Asian J.* **2009**, 4, 1248–1256.

[15] Yoon, D. H.; Lee, S. B.; Yoo, K. H.; Kim, J.; Lim, J. K.; Aratani, N.; Tsuda, A.; Osuka, A.; Kim, D. Electrical conduction through linear porphyrin arrays. *J. Am. Chem. Soc.* **2003**, 125, 11062–11064.

[16] Bengasi, G.; Desport, J. S.; Baba, K.; Fernandes, J. P. C.; De Castro, O.; Heinze, K.; Boscher, N. D. Molecular flattening effect to enhance the conductivity of fused porphyrin tape thin films. *RSC Adv.*, **2020**, 10, 7048-7057.

[17] Ganesh Moorthy, S.; King, B.; Kumar, A.; Lesniewska, E.; Lessard, B. H.; Bouvet, M., Molecular Engineering of Silicon Phthalocyanine to Improve the Charge Transport and Ammonia Sensing Properties of Organic Heterojunction Gas Sensors. *Advanced Sensor Research* **2023,** *2* (3), 2200030.

[18] Mateos, M.; Meunier-Prest, R.; Heintz, O.; Herbst, F.; Suisse, J.-M.; Bouvet, M. Comprehensive Study of Poly(2,3,5,6-Tetrafluoroaniline): From Electrosynthesis to Heterojunctions and Ammonia Sensing. *ACS Appl. Mater. Interfaces* **2018**, *10* (23), 19974–19986.

[19]. Mateos, M.; Meunier-Prest, R.; Suisse, J.-M.; Bouvet, M. Modulation of the Organic Heterojunction Behavior, From Electrografting to Enhanced Sensing Properties. *Sens. Actuators: B. Chem.* **2019**, *299*, 126968.

[20]. Bengasi, G.; Meunier-Prest, R.; Baba, K.; Kumar, A.; Pellegrino, A. L.; Boscher, N. D.; Bouvet, M. Molecular Engineering of Porphyrin-Tapes/Phthalocyanine Heterojunctions for a Highly Sensitive Ammonia Sensor. *Adv. Electron. Mater.* **2020**, *6* (12), 2000812.
